# Perturbation analysis of a multi-morphogen Turing reaction-diffusion stripe patterning system reveals key regulatory interactions

**DOI:** 10.1242/dev.190553

**Published:** 2020-10-29

**Authors:** Andrew D. Economou, Nicholas A. M. Monk, Jeremy B. A. Green

**Affiliations:** 1 Department of Craniofacial Development & Stem Cell Biology, King's College London, London, SE1 9RT, UK; 2 School of Mathematics and Statistics, University of Sheffield, Sheffield, S3 7RH, UK

**Keywords:** Morphogens, Palate, Patterning, Reaction-diffusion, Rugae

## Abstract

Periodic patterning is widespread in development and can be modelled by reaction-diffusion (RD) processes. However, minimal two-component RD descriptions are vastly simpler than the multi-molecular events that actually occur and are often hard to relate to real interactions measured experimentally. Addressing these issues, we investigated the periodic striped patterning of the rugae (transverse ridges) in the mammalian oral palate, focusing on multiple previously implicated pathways: FGF, Hh, Wnt and BMP. For each, we experimentally identified spatial patterns of activity and distinct responses of the system to inhibition. Through numerical and analytical approaches, we were able to constrain substantially the number of network structures consistent with the data. Determination of the dynamics of pattern appearance further revealed its initiation by ‘activators’ FGF and Wnt, and ‘inhibitor’ Hh, whereas BMP and mesenchyme-specific-FGF signalling were incorporated once stripes were formed. This further limited the number of possible networks. Experimental constraint thus limited the number of possible minimal networks to 154, just 0.004% of the number of possible diffusion-driven instability networks. Together, these studies articulate the principles of multi-morphogen RD patterning and demonstrate the utility of perturbation analysis for constraining RD systems.

This article has an associated ‘The people behind the papers’ interview.

## Abstract (precis)

**Highlighted Article:** Going beyond classic two-component Turing Reaction-Diffusion, this study uses inhibitors, modelling and developmental dynamics to define the FGF-Hh-Wnt-BMP network driving striped rugal patterning in the oral palate.

## INTRODUCTION

The generation of anatomy by self-organisation remains one of the most important subjects in the study of biology. It has acquired new importance as a guiding feature of regenerative medicine and the modelling of disease processes by the creation of self-organising organoids from stem cells (Werner et al., 2017). Setting aside mechanical self-organisation, a central idea of how self-organisation occurs chemically is that of reaction-diffusion (RD). In Turing's initial formulation, an initially stable system of two or more interacting morphogens can be destabilised (i.e. generate non-uniform distributions) into a periodic pattern through diffusion (Turing, 1952). This is referred to as a diffusion-driven instability (DDI). Gierer and Meinhardt later refined Turing's ideas and, among several refinements, introduced the formulation of the RD system as a system consisting of a slow-diffusing activator and fast-diffusing inhibitor (Meinhardt, 2012). Since the 1970s, numerous examples of RD behaviour have been described and analysed, and more recently the methods of molecular biology and biochemistry have identified a number of morphogen pairs, which are generally protein growth factors or growth-factor-binding proteins, that fit Turing's and Meinhardt's minimal description (e.g. Economou et al., 2012; Jung et al., 1998; Michon et al., 2008; Mou et al., 2006; Sick et al., 2006).

One aspect of self-organisation that has been set aside hitherto is that, as we now know, large proportions of the genome and proteome are devoted to regulation. Consequently, minimal systems must be expanded if they are to capture this complexity where it is functionally relevant. The extension of RD systems to include multiple morphogens and even non-diffusible components (Celliere et al., 2012; Klika et al., 2012; Marcon et al., 2016; Raspopovic et al., 2014) opens up a Pandora's box of possible descriptions of self-patterning systems. It raises the issue of what level of description is usefully interpretable and, importantly, experimentally tractable, such that empirical data and theory can be compared. We suggest that conceptually and pharmacologically, a usefully intelligible and accessible level of description is the signalling pathway: typically, this puts a family of protein growth factor ligands (e.g. the FGFs) together with their common receptors, transducers and target genes into a single unit. This provides a potentially happy balance between, on the one hand, the abstraction of a pure two-component morphogen system, the ‘morphogens’ of which bear little relationship to the single specific diffusible chemical species envisaged by Turing or even specific growth factors, and, on the other hand, the overwhelming complexity of an ‘omic molecular network, the intelligibility of which would inevitably require dimensional reduction in any case.

Taking this level as our motivating principle [while acknowledging that it is a first approximation (see Discussion)], we contemplate how far one can get in defining a model (i.e. a network topology) of key interactions that can capture the behaviour of the system, when considering multiple signalling pathways. We analyse the periodic pattern that generates the transverse ridges on the roof of the mouse palate, the rugae, a striped pattern that we previously showed to exhibit RD system behaviour (Economou et al., 2012). Using chemical inhibitors and pathway target expression analysis together with analytical and numerical simulation strategies, we were able to identify topological motifs that are predictive of the behaviour of the system, regardless of the number of components considered in the network. Through further consideration of the dynamics of pattern formation, we have constrained the model possibilities to a small number of consistent topologies. In doing so, we identify a useful theory-experiment dialogue that generates specific hypotheses amenable to practical progress in understanding the dynamic behaviours of this example of a self-organising RD system.

## RESULTS

### Chemical inhibitors implicate FGF, hedgehog, Wnt and BMP pathways in periodic ruga patterning

We previously showed that the periodic stripes of expression of the sonic hedgehog (*Shh*) gene in the mouse mid-gestation palate depend on the Hh pathway itself as an ‘inhibitor’ and the FGF pathway as an ‘activator’ (Economou et al., 2012). We were deliberately agnostic about the specific FGF ligand-receptor pair that was crucial because multiple FGFs and FGF receptors are expressed in the palate (Porntaveetus et al., 2010). Both FGF and SHH are, by a number of experimental criteria, secreted diffusible morphogens (Bökel and Brand, 2013; Dessaud et al., 2007). As we acknowledged, there were already published data implicating Wnt and potentially BMP as additional morphogens (Lin et al., 2011; Welsh and O'Brien, 2009). To go beyond the simple two-component description of the patterning network, we sought first to determine the requirement for each of these four morphogen pathways using an established explant system, as before. We exposed embryonic (E) day 13.5 palatal explants for 24 h to cyclopamine (a hedgehog pathway inhibitor), SU-5402 (a FGF pathway inhibitor), IWP-2 (a Wnt pathway inhibitor) and dorsomorphin (a BMP pathway inhibitor), and tested the effect of these pathway-specific inhibitors on the pattern. Efficacy of these treatments was confirmed by probing for expression of direct transcriptional targets of each pathway: *Spry1*, *Ptch1*, *Lef1* and *Id1*, respectively (Economou et al., 2012; Fig. S1).

**Fig. 1.**
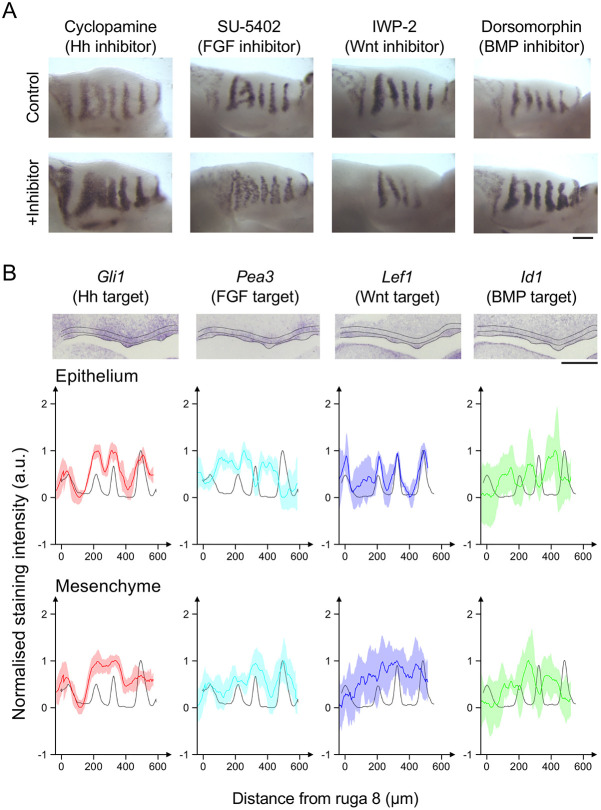
**Target expression and responses to inhibitors reveal involvement of Hh, FGF, Wnt and BMP pathways in periodic rugae patterning.** (A) *Shh in situ* hybridisations on E13.5 palatal shelf explants cultured for 24 h in the specified small molecule inhibitor contralateral shelves as vehicle controls. Anterior to the right, medial up. (B) *In situ* hybridisation of sagittal sections of E13.5 palatal shelf for specified genes. Dotted lines illustrate the extent of the palatal epithelium and the underlying mesenchyme used for quantifications. Anterior to the right. The intensity profile averaged across the palatal shelf shown for each specimen from which the illustrated *in situ* is taken for the gene of interest (coloured trace) and *Shh* (grey trace) for the epithelium and mesenchyme. Shaded areas represent 1 s.d. around the gene of interest (for clarity of presentation the variation around *Shh* trace is not shown). For each marker, the number of specimens showing the observed pattern (essentially the number of specimens from which the kymographs in Fig. 6 were made) are: *Shh*, 114; *Gli1*, 42; *Pea3*, 41; *Lef1*, 41; and *Id1*, 34. a.u., arbitrary units. Scale bars: 200 µm.

We used the stripes of *Shh* expression as our readout of the pattern (although in principle, any of the components could be used as a readout). Fig. 1A shows that inhibition of each of the four pathways has an effect on the *Shh* expression stripes. Through careful quantification (Figs S2, S3), we could demonstrate that although some inhibitors changed the number of *Shh* stripes, none of the inhibitors changed the position of the stripes (and therefore the wavelength of the pattern) relative to controls (Fig. S3A,B,E). Rather, inhibition changed the width and *Shh* staining intensity of stripes (Fig. S3C-E). Specifically, dorsomorphin intensified and broadened the stripes similar to what we previously reported for cyclopamine, whereas IWP-2 narrowed and weakened them. FGF inhibition by SU-5402 also weakened the stripes, as previously reported (Economou et al., 2012), but careful inspection over a number of trials also revealed consistent broadening as well as weakening (Fig. 1A, Fig. S4). In some cases, posterior and anterior stripes differed in the magnitude of their response. On cyclopamine or SU-5402 treatment, posterior rugae appeared to widen more than anterior rugae, in some cases to the point of fusing with their neighbours (Fig. S3B,C), whereas on IWP-2 treatment, anterior rugae decrease in staining intensity more than posterior rugae, and in some cases to below the detection threshold leading to the apparent loss of stripes (Fig. S3B,D). However, although the magnitude of response could vary along the palate, the direction of response was the same across all *Shh* stripes (Fig. S3E). These results confirm that all four pathways contribute to the formation of the ruga pattern, and their inhibition changes the width and intensity of stripes but not the wavelength of the pattern.

We focused our attention on the anterior stripes (black points in Fig. S3C,D), which were already established before the addition of the inhibitor (as opposed to the posterior stripes, which were in a region of new stripe formation) and which we previously demonstrated were maintained by an RD mechanism through their ability to bifurcate on the removal of a neighbouring stripe (Economou et al., 2012). Although these stripes showed a weaker response to the inhibition of Hh and FGF signalling, and a stronger response to the inhibition of Wnt signalling than the posterior stripes, we speculate that all stripes involve the same RD mechanism and that these differences in magnitude relate to the difference between perturbing an already established steady-state pattern versus perturbing a non-steady-state region in which an unpatterned state is undergoing destabilisation.

### Transcriptional pathway targets are expressed periodically and in different spatial phases

Although each of the pathway ligands are casually referred to in the literature as a morphogen, in the RD sense any of these could in fact be a uniform permissive component of the system rather than part of the periodicity-generating network. To be an RD-type patterning morphogen, the activity of its pathway must also be periodic. To determine where each of these pathways is active, we analysed well-established direct transcriptional targets of their respective transduction pathways (Fig. 1B, Fig. S5). Using pathway targets as the operational measure of pathway activity avoids the complications of multiple ligands, receptors and transduction components as discussed above. We found that each of the pathways had periodic outputs. Where available, additional target markers were tested and gave the same periodic pattern (Fig. S6). As expected, Hh target expression was in the same spatial phase as *Shh* in both epithelium and mesenchyme. Surprisingly, FGF signalling was in-phase with the *Shh* stripes in the mesenchyme but out-of-phase in the epithelium. This reconciles our previous description of FGF activity as being in-phase with the *Shh* stripes (Economou et al., 2012) with a previous report describing it as out-of-phase (Porntaveetus et al., 2010). It also means that FGF signalling must be functioning as effectively two different pathways (defining a pathway as its transcriptional end-point). We refer to these pathways as mesenchymal FGF (mFGF) and epithelial FGF (eFGF). Based on expression patterns, these are most likely to be FGF10/FGFR1 and FGF3/FGFR2 ligand/receptor pairs, respectively (Porntaveetus et al., 2010). Wnt target expression was in-phase in the epithelium and undetectable in the mesenchyme, whereas BMP signalling was out-of-phase in both layers (Fig. 1B). In summary, Hh, mFGF and Wnt (pathways) are in-phase with the rugae, and eFGF and BMP (pathways) are out-of-phase. The above findings show that each of the pathways is periodic and are therefore, by definition, part of a periodic pattern-generating network.

### Multiple two-component RD networks are consistent with the phase and perturbation data

We then investigated how four or potentially five components can be wired together in a regulatory network that can generate the observed spatial pattern. One potentially simplifying approach is to consider whether the topology of this system (i.e. the network, technically a directed graph, in which chemical components are linked by activation or inhibition arrows) could effectively be described as a classical two-component RD system, which would consist of two ‘master morphogens’, with the other components serving as intermediates [so-called ‘mediators’ (Cho et al., 2011)] between them. This would be the case if the exact topology of a two-component system between two master morphogens was preserved in a larger system. Effectively, interactions between master morphogens in the two-component system would be expanded by passing through a series of mediators in which the net sign of the interactions is the same (Fig. S7A). Any pair of morphogens could potentially support a two-component system [if ‘in-phase’ by a classical activator-inhibitor (AI), or if ‘out-of-phase’ by a substrate-depletion (SD) configuration].

We therefore turned to perturbation analysis as a way of constraining possible network topologies. Using simple linear equations, we modelled the effects of perturbing either component in both classical two-component AI and SD systems. We investigated how a series of waves, generated by a two-component RD network from noisy initial conditions, would respond to the inhibition of each component. Preliminary investigations (Fig. S8) indicated that although the pattern of waves could be lost under strong inhibition, the series of waves could be preserved (including the wave number) if inhibition strength was reduced (Fig. S8D,E). However, upon inhibition, either the amplitude or the absolute level of the waves was shifted up or down. It should be noted that in some instances, the waves appeared bounded. For example, if a wave shifted up, only the troughs of the wave moved up, whereas the peaks did not move noticeably, and vice versa if the wave shifted down. (This appeared to be the case for the auto-activating component of a two-component RD system, where to form a stable series of waves the upper and lower production rates were bounded.) In these bounded cases in particular, the upward or downward shift in wave position was associated with a stripe widening or narrowing, respectively (Fig. S8F,G). The maintenance of wave number along with changes in width and intensity was reminiscent of the changes in *Shh* stripes seen in our inhibitor-treated explant cultures.

**Fig. 2.**
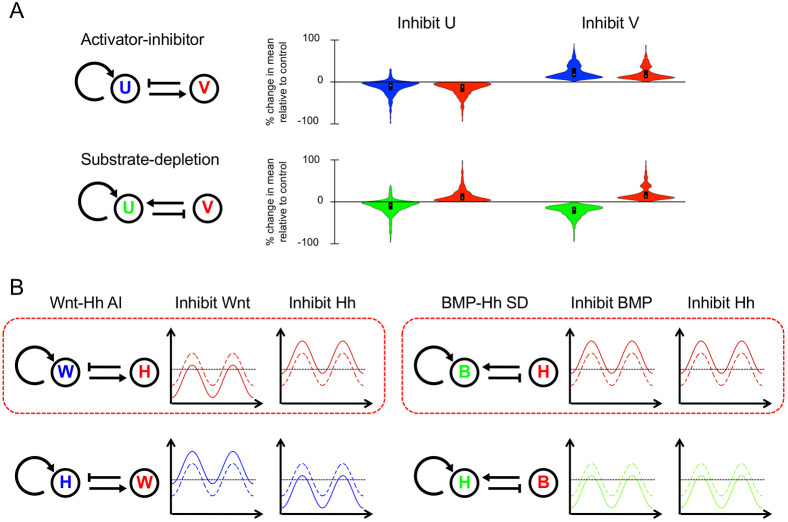
**Numerical simulation of RD patterning inhibition for two-component systems.** (A) Violin plots showing percentage change in the mean level of components U and V in illustrated AI and SD RD networks upon inhibition of the response to morphogens U and V in RD simulations (plots for inhibition of production are shown in Fig. S9). (B) Networks showing the two possible configurations of Wnt-Hh AI systems and BMP-Hh SD systems, with components coloured according to the equivalent component in A. Associated with each network is a schematic of the response of Hh upon inhibition of each component in the network, based on the predominant response in the simulations. Solid lines indicate levels after inhibition, with dashed lines representing uninhibited states. Horizontal dotted lines represent an arbitrary detection threshold. The two topologies that have responses to inhibition that replicate the experimental observations (see Fig. 1A) are highlighted with a red box. B, BMP; H, Hh; W, Wnt.

As the behaviour of the waves upon inhibition could be described as upward or downward shifts, we could capture it as shifts in the mean level of each component. Therefore, we generated 1000 random parameter sets consistent with the conditions for DDI for both AI and SD networks, and only considering parameter sets that could produce a stable series of waves, we determined the change in the mean component level upon inhibition (Fig. 2A, Fig. S9), ensuring that wave number was maintained after inhibition (Table S1). We confirmed that, for almost all parameter sets, the change in mean component level appeared to be the result of a shift in wave amplitude or absolute level, as illustrated in Fig. S8F,G (see also Table S1). We then compared the results with the effects on established spatial patterns of experimentally inhibiting Wnt, BMP or Hh itself, considering first all four possible two-component systems containing the Hh pathway (our readout) and excluding FGF [where two nodes are inhibited simultaneously requiring a different analysis (see below)]. We found that for both Wnt-Hh and BMP-Hh systems, one of the two possible two-component models was consistent with the data (Fig. 2B). However, there exists no three-component Wnt-BMP-Hh network which can be interpreted as both a master Wnt-Hh AI topology with BMP mediating a single interaction, and a master BMP-Hh SD topology with Wnt mediating a single interaction (Fig. 2, Fig. S7B,C). This showed that our system (probably like most real systems) cannot be accurately described as falling into either one of the two classical AI or SD classes.

### Analysis and numerical modelling identifies well-connected network topologies capable of non-oscillating diffusion-driven instability

The above results showed that modelling this system requires consideration of higher order (i.e. greater than two-component) networks. Therefore, we next investigated whether any three-component networks could be found that were consistent with the spatial pattern and perturbation data. There are 3^9^=19,683 possible three-component network topologies, as each component can interact with the two others and itself (nine types of interaction) and each interaction can be positive, negative or zero (Fig. S10). Considering that all components have some degradation rate, and therefore have some level of negative self-interaction, we only considered the distinction between topologies with positive self-interactions and those without them. This reduces the total number of possible topologies to 2^3^×3^6^=5832. [These different topologies incidentally place different constraints on diffusion, as discussed by Marcon et al. (2016).]

To go further, we resorted to numerical methods because, unlike for the two-component system, there is not a well-established relationship between network topology and spatial patterning for three-component networks (Scholes et al., 2019). Three-component RD systems were considered by White and Gilligan in the context of hosts, parasites and hyperparasites (White and Gilligan, 1998), in which they described criteria that determine which such systems generate DDI, as well as criteria to distinguish temporally stable from oscillating systems. More recently, related analyses, including graph-based approaches, have been applied to developmental periodic patterning (Marcon et al., 2016) and general RD systems (Diego et al., 2018; Scholes et al., 2019). We systematically screened parameter ranges around known values published for biological RD systems (Nakamasu et al., 2009) and applied the White and Gilligan (1998) criteria for DDI in a three-component model. Out of 242,121,642 parameter sets (9 interactions×7 values for each×6 different choices for which morphogens have high or low diffusion values) searched we found a subset of 653,574 parameter sets that gave DDI. The signs of the parameters define specific topologies. As the rugae form as stable stripes of gene expression and tissue thickening (Economou et al., 2013, 2012), our first observational constraint was that the stripes we see are non-oscillating. We recovered 1492 topologies giving non-oscillating DDI (see Materials and Methods for details). A second constraint was that inhibition of any of the components would perturb the pattern. This implied that every node (morphogen) in the network outputs as well as inputs, making the network ‘strongly connected’ (Marcon et al., 2016). We recovered 1396 strongly connected network topologies. Among these, the four possible phase relationships (all-in-phase or each one of the three out-of-phase with the other two) could each be generated by 498 different topologies (with some topologies capable of generating more than one possible phase relationship depending on parameter values).

**Fig. 3.**
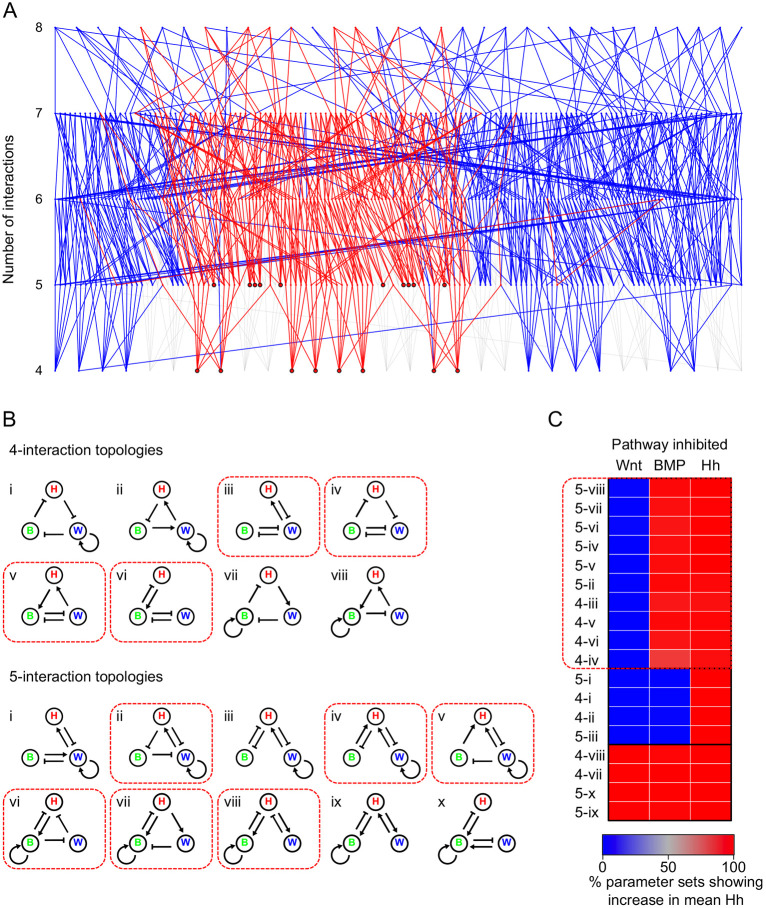
**‘Stalactite plot’ and numerical simulation identifying subsets of three-component RD systems and their behaviours under inhibition.** (A) Topology atlas for the phase group of the three-component network identified in the parameter search, which is consistent with the spatial pattern of Wnt, BMP and Hh. Topologies that can be recovered with fast-diffusing Hh and slow-diffusing Wnt and BMP are in blue. Out of the 45 strongly connected topologies that are found at the bottom of the stalactites, the 18 that are also consistent with the diffusion constraints are outlined in black. For completeness, not strongly-connected topologies are shown in grey along with their relationship to the strongly connected topologies. (B) Graphs showing the 18 Wnt-BMP-Hh networks divided into four-interaction and five-interaction networks. Within each group, networks are numbered according to their position from left to right in A. (C) Heat map showing the percentage of parameter sets in which the level of Hh increases in response to the inhibition of each component in the network in RD simulations. Topologies are grouped according to hierarchical clustering (see Fig. S10). The ten topologies that have a response that is consistent with the experimental data are highlighted with a red box, as are the network diagrams in B. B, BMP; H, Hh; W, Wnt.

To investigate the 498 topologies in a given phase group, we generated ‘stalactite’ plots according to Cotterell and Sharpe (2010). In this representation, each topology is shown as a point linked to all other topologies that differ from it by the gain/loss of one edge, with each row corresponding to the total number of edges. Topologies at the base of each stalactite, of which there were 45 for each phase group, represent those that are distinct (non-overlapping) and possess only non-redundant interactions (Fig. 3A). It should be noted that these topologies are slightly different to the minimal topologies defined by Diego et al. (2018) as we assume that all biological components have some degradation rate, and therefore do not include these interactions.

### Spatial phase and perturbation outcomes further constrain the number of possible real networks

We could now begin to consider real morphogens as nodes in the identified topologies, to investigate which topologies could correspond, first, to the observed spatial phase relationships and, second, to likely diffusivities. We considered three-node networks consisting of Hh, Wnt and BMP. We knew the phase relationship of these from direct observation of the targets shown in Fig. 1: Hh and Wnt activities are in-phase with the rugae and BMP activity is out-of-phase. This constraint identified a specific phase group of 45 topologies as relevant to our system. As for diffusivities, to simplify the analysis we first set Hh as the fast-diffusing pathway compared with the other two, consistent with its role in our two-component analyses (Fig. 2B). (We show below that this provisional assumption is ultimately not required for the selection of networks consistent with the experiment.) These phase and diffusivity constraints reduced the number of working topologies down to just 18 (Fig. 3A,B).

With these 18 topologies, we now compared their predicted behaviour under perturbation with the experimental results using numerical simulations similar to those described above (see Materials and Methods for details). Inhibiting each component for 1000 randomly chosen DDI parameterisations of each topology revealed three groups of characteristic responses to inhibitions (Fig. 3C, Fig. S11, Table S2). Ten out of the 18 networks showed Hh pathway responses consistent with the experimentally observed changes in *Shh* expression (boxed in Fig. 3C).

### Identification of feedback loop signs enables prediction of network behaviour under perturbation

From several thousand conceivable networks, the above analytical, numerical and experimental methods identified just ten three-component minimally connected topologies that captured the experimentally observed periodicity, phase and perturbation responses of three of the five components identified as active in this system. We will refer to these as ‘valid’ networks. How might this help us discover what networks would be valid that include all five components, and potentially ‘*n*’ additional components? It has been suggested that general rules for biological circuit behaviour can be inferred from analysis of the signs of the embedded feedback loops (Tyson and Novak, 2010). We therefore examined whether this approach might be applied to our RD networks. We observed that our valid three-component networks contained the same feedback loops as the two perturbation-consistent two-component networks (Fig. 2B). In the four-interaction networks, Wnt and BMP are in positive feedback loops by mutual inhibition, whereas Hh is always only in a negative feedback loop (Wnt and BMP can also be found in the negative feedback loop), and in the five-interaction networks, Wnt and BMP are still in a positive feedback loop through (direct or indirect) mutual inhibition, whereas the exact interactions, and therefore the feedback loops of either two-component system, are embedded in the network (Fig. 3B, Fig. S12 indicate the loops). In other words, the behaviour of the network, including under perturbation, is embedded in the product of the signs of the ‘arrows’, i.e. the signs of the reaction term coefficients. Indeed, Marcon et al. (2016) and Diego et al. (2018) recently demonstrated that the formation of patterns in RD systems is dependent on these destabilising positive feedback and stabilising negative feedback loops. To investigate whether the response to perturbation is also determined by these loops, we analysed the relationship between reaction terms (i.e. the RD equations without the diffusion terms) and the behaviour of components in response to perturbation. We showed that this has a consistent mathematical form (asymptotic curves), which enables the sign of the perturbation response (as opposed to its precise magnitude) to be predicted as a relatively simple set of conditions fulfilling or not fulfilling certain inequalities (see Appendix S1, section 2.1). Through comparison with simulations of the full RD system (see Appendix S1, section 2.2) we found that for all but very small perturbations (in which diffusion can dominate) or very large perturbations (in which RD breaks down altogether), that it is indeed possible to predict the behaviour of an RD-competent network from its reaction terms alone. Analytical considerations reinforce this conclusion (see Appendix S1, section 2.2).

**Fig. 4.**
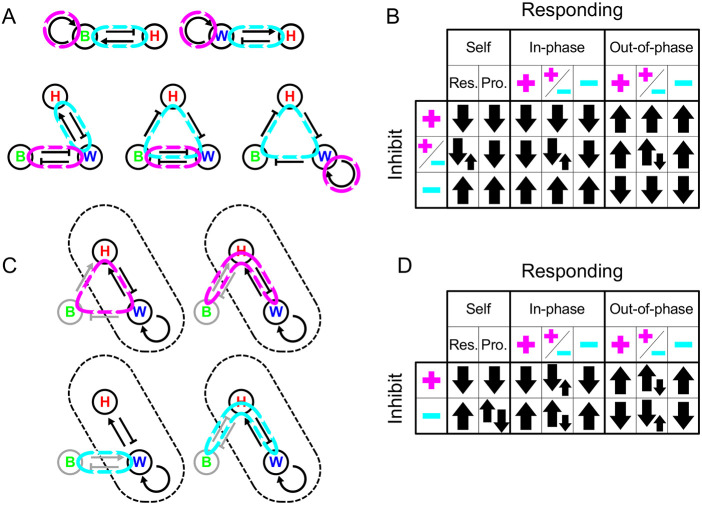
**Identification of feedback loops and resulting behaviours under inhibition for three-component RD systems.** (A) Illustrative two- and three-component networks showing minimal feedback requirements for RD of one or more components forming a single positive feedback loop (highlighted in magenta), with a negative feedback loop being formed through at least one additional component (highlighted in cyan). (B) Summary constraint table showing the response of components in such a minimal network to the inhibition of a component found in either the positive feedback loop alone (magenta ‘plus’ sign), negative feedback loop alone (cyan ‘minus’ sign) or both (‘plus’ and ‘minus’), depending on which loop they are in and the phase relative to the inhibited component (‘in-phase’ or ‘out-of-phase’). For the response of a component to its own inhibition (‘self’), the inhibition of response (‘res.’) and production (‘pro.’) are shown. Upward pointing arrows indicate an increase in the level of a component, whereas downward arrows indicate a decrease. Two arrows of equal size show when the system is unconstrained. Where opposing large and small arrows are shown, the system behaves according to the large arrows, apart from under certain topologies (see Appendix S1, section 4) in which the reverse is seen for certain components. Although components are not constrained, different components showing the unconstrained response are coupled to one another. (C) Illustrative examples showing how additional components can be integrated into a minimal RD system outside of the ‘core’ RD network by forming external loops. External components and interactions are shown in grey, either forming positive or negative feedback loops with the core positive feedback loop (highlighted in magenta and cyan, respectively). Core RD network outlined with dashes. (D) Summary constraint table showing the response of components in such a network to the inhibition of a component that provides either additional positive feedback (magenta ‘plus’ sign) or additional negative feedback (cyan ‘minus’ sign) to the core positive feedback loop. The table shows how this depends on which loop in the core network they are in [response (Res.); production (Pro.); +, ± or −] and the phase relative to the inhibited component. Symbols as in B. For a subset of topologies in which a component is in both loops see Appendix S1, section 4. B, BMP; H, Hh; W, Wnt.

To further explore the relationship between the network topology and the response to perturbation, we expressed the terms in the inequalities as functions of the combination of signs of the reaction terms (i.e. feedback loops) in *n*-component systems, thus predicting the effects on perturbation of any given RD system component, depending on its participation in positive and/or negative feedback loops (see Appendix S1, section 3). We were able to use this approach to analyse how the response of components to inhibition in the system changes upon stepwise addition of new nodes (components) (see Appendix S1, section 4). In brief, and consistent with the results of an analysis into the topological requirements for RD by Diego et al. (2018), an RD system requires that for an *n*-node system, there needs to be a positive feedback loop through a maximum of *n* – *m* nodes and a negative feedback loop through the remaining *m* nodes (provided *n*>*m*≥1), and at least one node must be in both loops. Examples of loops in two-node (classical RD) and three-node systems are shown in Fig. 4A. With these conditions in mind, one can predict outcomes of perturbations of each type of node included in these loops (Fig. 4B). However, the above conditions allow for networks in which these loops go through only a subset of nodes. The predictions of outcomes of perturbations in Fig. 4B therefore need not apply. Further analysis (described in Appendix S1, section 4) generated predictions of what happens to components represented by nodes outside these RD-defining ‘core’ loops (Fig. 4C,D). We found the effects of inhibition of these ‘non-core’ components to be mostly similar, but not identical, to those of inhibition of core components. [This means that sometimes alternative ‘cores’ in a given network will produce different perturbation responses (see Discussion below).]

Incidentally, this feedback loop decomposition can also help explain the seemingly counterintuitive nature of pattern formation in some topologies. For example, topology iv in Fig. 3B can produce a periodic pattern despite only consisting of negative interactions. In this topology, positive feedback loop of the RD is produced by the mutual inhibition between Wnt and BMP, whereas the negative feedback loop comes from the cycle of three inhibitory interactions between Wnt, BMP and Hh.

We applied these rules for perturbation responses and effects of node addition to the 45 Wnt-BMP-Hh networks discussed above that fulfilled the requirements of diffusion-driven instability and the observed phase relationships. Systematically identifying all possible loop combinations (Fig. S13) enabled the prediction of the responses of each component to any inhibition using the constraint tables in Fig. 4. This showed that the observed perturbation responses corresponded to the same ten topologies as in Fig. 3 but in this case without the prior assumption that Hh was the fast-diffusing component.

**Fig. 5.**
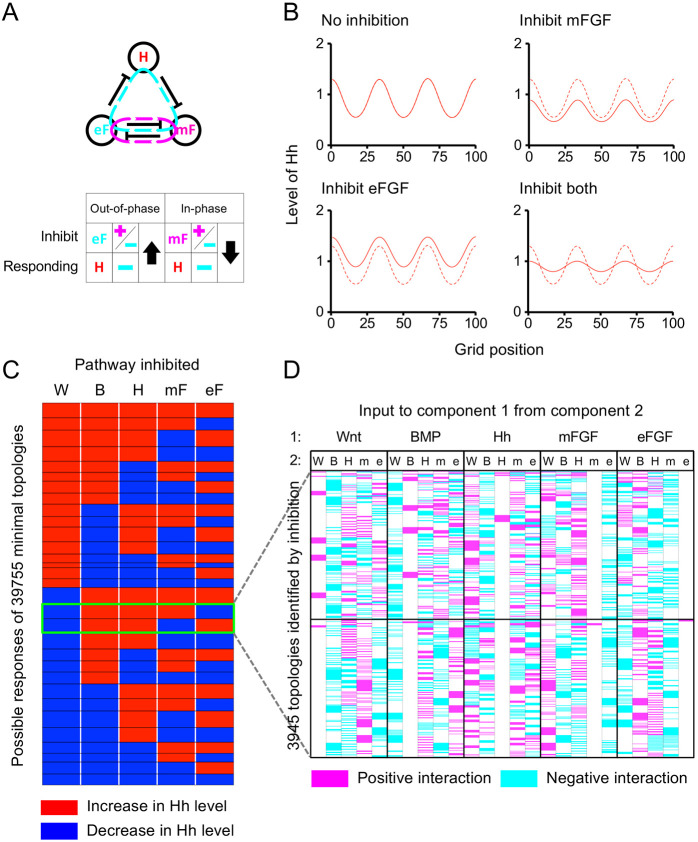
**Integration of two out-of-phase FGF morphogens explains FGF-inhibition effects and allows the prediction of behaviours under inhibition of five-component RD systems.** (A) Example of a network where the inhibition of epithelial FGF (eF) and mesenchymal FGF (mF) would be predicted to have opposing effects on the levels of Hh (H) according to analysis of reaction terms, alongside an illustration of how this is determined through the constraints imposed by the different feedback loops in the system [positive feedback loop alone (magenta ‘plus’ sign), negative feedback loop alone (cyan ‘minus’ sign) or both (‘plus’ and ‘minus’), depending on which loop they are in and the phase relative to the inhibited component (‘in-phase’ or ‘out-of-phase’)]. Positive and negative feedback loops are shown in magenta and cyan, respectively. (B) Simulation of single and combined inhibition of the two FGF components (dashed lines indicate uninhibited state). Simulations carried out as detailed in Materials and Methods. *u*_1_ is mF, *u*_2_ is eF and *u*_3_ is H, with *a_12_*=−0.019, *a_13_*=−0.034, *a_21_*=−0.019, *a_32_*=−0.022, *b_1_*=0.064, *b_2_*=0.037, *b_3_*=0.068, *c_1_*=0.004, *c_2_*=0.011, *c_3_*=0.039, *fmax_1_*=0.008, *fmax_2_*=0.022, *fmax_3_*=0.072, *D_1_*=1.03, *D_2_*=1.71 and *D_3_*=7.63. Where not specified a_ij_=0. Initial conditions drawn from a random distribution, as described in Materials and Methods. (C) Map showing all possible responses of Hh to inhibition of each of the five components for all 39,755 predicted minimal topologies (red, increase in Hh; blue, decrease in Hh). Topologies arranged into 31 different groups of responses, outlined in black. Two sets of responses that constitute 3945 topologies showing the observed responses (Wnt down, BMP up, Hh up, and mFGF and eFGF opposing responses) highlighted in the green box. (D) Map showing the interactions making up the 3945 topologies identified by perturbation analysis (positive interactions in magenta, negative in cyan, no interaction in white). Horizontal black line separates topologies giving different patterns of responses of Hh in response to mFGF and eFGF inhibition (upper group of topologies correspond to the upper group of highlighted topologies in C). B, BMP; H, Hh; W, Wnt.

The effect of inhibition of FGF can now be reconsidered. As the effect on *Shh* expression was neither net increase nor net decrease, we considered topologies in which eFGF and mFGF acted in opposition. Simulation with a parameterisation of the very simple network of this kind depicted in Fig. 5A showed that a blended increase and decrease leading to a flatter waveform was indeed obtained (Fig. 5B). Thus, an RD system with two FGFs, each having opposite effects on *Shh*, can account for our experimental observations.

We considered all possible five-component topologies with loops required for DDI with the sign of links constrained by their phase relationship (see Appendix S1, section 4). Specifically, we first identified all possible sets of core topologies (i.e. all subnetworks of two to five components that have the minimal number of interactions sufficient for RD). For each core, all possible combinations for wiring-in the remaining components were recovered (see Materials and Methods for details). This yielded 39,755 unique minimal topologies. Prediction of their responses to inhibition revealed that, of these, 3945 were consistent with our experimental perturbation results. Fig. 5C,D depicts these topologies in terms of the interactions (arrows) between the five components. Although some interactions are different in different topologies, some are the same or absent for all topologies [e.g. the input of mFGF to eFGF indicated in the column second from the right are inhibitory (cyan) or absent (white) in all topologies].

### Developmental dynamics of stripe appearance reveal an eFGF-Wnt-Hh ‘core’ network with mFGF/BMP incorporation after stripe establishment

**Fig. 6.**
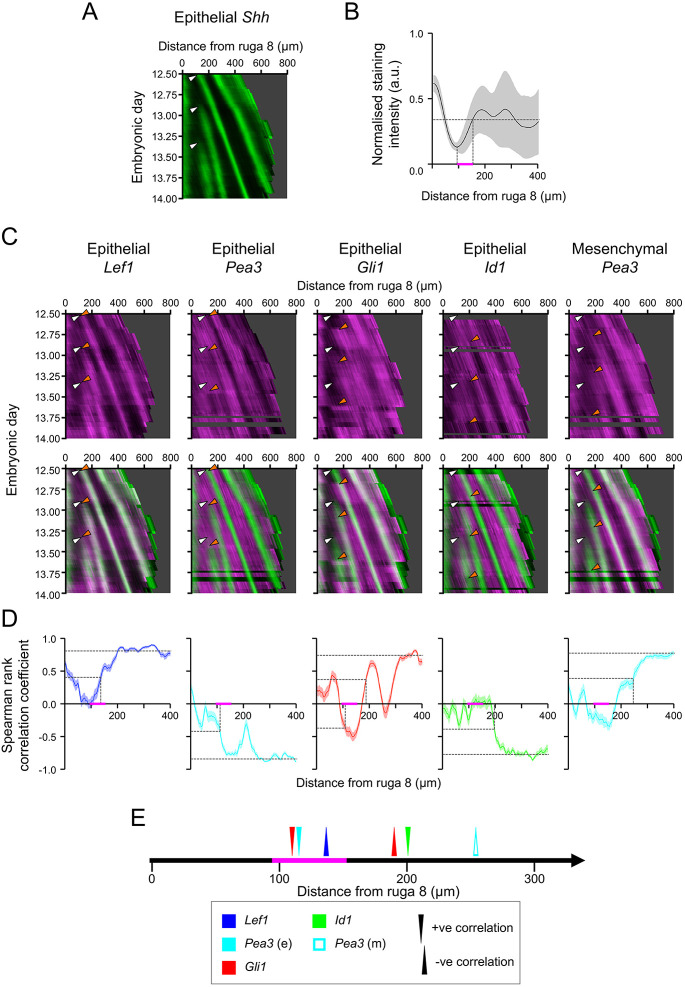
**Periodic gene expression kymographs reveal an early Wnt-eFGF-Hh initiating ‘core’ system with mFGF and BMP integrated later.** (A) Kymograph showing the pattern of expression of *Shh* through time. White arrowheads indicate the approximate onset of *Shh* expression for each ruga. (B) Plot of mean normalised intensity of *Shh* staining for each AP position relative to ruga 8. The magenta bar denotes the period of onset of *Shh* expression, bounded by the minimum in staining intensity and the position at which the staining intensity plateaus (vertical dashed lines). Horizontal dashed line shows level at which *Shh* intensity plateaus (as determined by the mean intensity of the anterior third of the palate). Shaded area represents 1 s.d. (C) Kymographs showing the pattern of expression of indicated target genes through time (magenta) and their expression relative to *Shh* (green) for rugae 3, 4 and 5. White arrowheads as in A, and orange arrowheads indicate approximate positions of the change in the expression pattern of each target gene associated with each ruga. Mesenchymal *Gli1* and *Id1* expression resemble the epithelial patterns (see Fig. S16) (horizontal dark bands in the red channel are stages for which too few specimens were obtained to allow interpolation). (D) Plot of the Spearman’s rank correlation coefficient for the intensity of *Shh* staining and the marked target gene across all time points for each position relative to ruga 8, indicating when, relative to the onset of *Shh* expression, the spatial pattern for each target gene emerges. Horizontal dashed lines represent maximal correlation coefficient, calculated over the anterior third of the palate (Fig. S15D) and half this value. Vertical dashed lines represent AP position where this level is first reached. Lower dashed lines for *Gli1* show where the half maximal level is reached for the opposite correlation (i.e. out-of-phase rather than in-phase). Shaded area represents 95% confidence interval from bootstrapping (see Materials and Methods). The magenta bar represents the period of onset of *Shh* expression, as determined in B. (E) Sequence whereby different targets come into (upward pointing arrowheads) or out-of (downward pointing arrowheads) phase with *Shh* relative to the distance from ruga 8, based on the correlation analysis in D. The magenta bar represents the period of onset of *Shh* expression, as determined in B. a.u., arbitrary units.

What further experimental data could constrain potential network topologies? We turned to temporal behaviour of the rugal system. We conducted a large number of *in situ* hybridisations on adjacent sections from a long time-series of specimens, spatially registering multiple specimens to the stripes of *Shh* expression. Because the pattern arises through monotonic linear growth (Economou et al., 2012), we could temporally order and space our specimens (see Fig. S14 and Materials and Methods for details on converting palatal lengths to time). This allowed us to determine the kinetics of the onset of stripe formation for each of the pathway markers relative to *Shh* expression onset (Fig. 6A-E). Moreover, as tissue growth occurs immediately anterior to ruga 8, it follows that older tissue lies more anterior in the palate. Therefore, by using anteroposterior (AP) position as a proxy for time and correlating the different transcriptional target genes with *Shh* at increasing anterior positions, we could follow the sequence of signalling events in ruga formation by determining where (and therefore when) the different transcriptional targets come into (or out-of) phase with *Shh* (Fig. S15).

The loss of FGF target *Pea3* (also known as *Etv4*) in the epithelium and expression of the Wnt target *Lef1* are the first instances of markers establishing their spatial patterns, and are simultaneous with the onset of *Shh* transcription (Fig. 6C-E). On the other hand, the increase of Hh target *Gli1*, downregulation of BMP target *Id1* and the increase of FGF target *Pea3* in the mesenchyme lag the onset of *Shh* expression. However, the correlation analysis also shows that the onset of *Shh* expression (and therefore the downregulation of *Pea3* and the upregulation of *Lef1*) is concomitant with a transient negative correlation and, therefore, reduced expression of *Gli1*. This transient period of reduced Hh signalling at the onset of stripe formation in a region of tissue growth is consistent with a role for Hh in providing negative feedback.

**Fig. 7.**
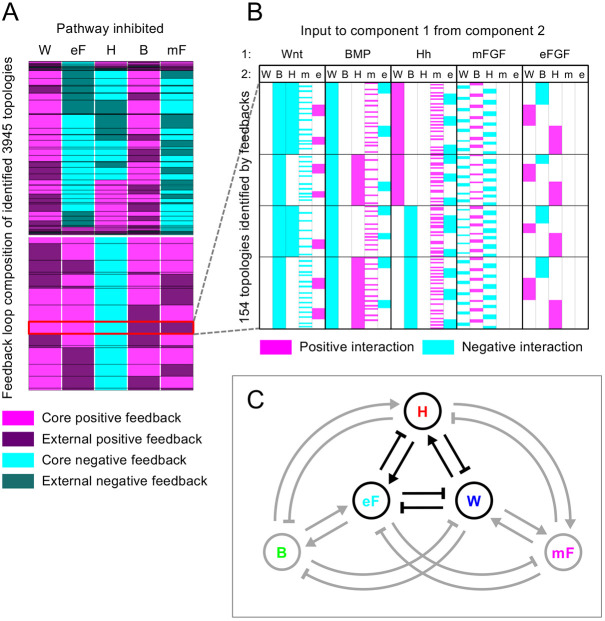
**Possible feedback loops and network topologies constrained by the experiment.** (A) Map showing feedback loop structure of 3945 topologies by perturbation analysis (see Fig. 5C,D). Components involved in positive feedback are in magenta and those involved in negative feedback are in cyan, with components external to the core in dark. Horizontal white line separates topologies giving different patterns of responses of Hh in response to mFGF and eFGF inhibition (upper group of topologies correspond to upper group of highlighted topologies in Fig. 5C,D). Groups of topologies showing different patterns of feedback loops are outlined in black. A set of 154 topologies showing constraints on topology as determined by kinetic analysis (Wnt and eFGF as only core positive feedback components and Hh as core negative feedback component) boxed in red. (B) Map showing the interactions making up the 154 topologies identified by feedback loop analysis (positive interactions in magenta, negative in cyan, and no interaction in white). Horizontal black lines separate four groups of topologies with different interactions between the three core components Wnt, eFGF and Hh. (C) The 154 topologies identified in A and shown in B, all show the same signs of interactions where present. Summary network diagram showing the signs of the interactions found among these topologies. B, BMP; H, Hh; W, Wnt.

What do the dynamic data mean for our networks? As eFGF and Wnt responses are the first movers and move simultaneously, they must form a core positive feedback loop together with Hh providing the negative feedback loop necessary for RD. As BMP and mFGF responses trail Hh, they cannot be part of the core positive feedback loop that destabilises the system. Applying these constraints to the 3945 topologies that were consistent with the experimentally observed phase relations and responses to perturbation gave 154 topologies consistent with the observed dynamics (Fig. 7A,B). This number constitutes 0.004% of the total 39,755 DDI minimal topologies. All of these are subsets of the same set of regulatory interactions (Fig. 7C), comprising one of four possible Wnt-eFGF-Hh cores, with BMP and mFGF providing additional interactions.

## DISCUSSION

We have shown that five classical morphogen pathways periodically pattern the rugae and have identified a relatively small number of potential RD network topologies, and an even smaller number of consistent regulatory interactions between nodes within such networks. The modelling, combined with the experimentation thus far, suggests, although of course does not prove, potentially direct molecular interactions between specific pathway-induced transcription factors and target enhancer sites in the genes encoding the other morphogens.

This study highlights more generally the ways in which experimental data can be used to challenge RD models in a mammalian tissue context. In particular, we have shown that the effects of relatively small acute perturbations to spatial patterns that have already formed are highly constraining on plausible network topologies. This suggests that similar inhibitor studies can be a useful complement to ‘knockout’ genetics in understanding these dynamical systems. We also gained constraining information from the dynamic (temporal) evolution of the system during embryonic development. This was facilitated by the sequential appearance of the stripes, which is a peculiarity of this rugal palate system. In many periodic patterns, such as, for example, the appearance of cartilage ring-patterning stripes in the developing trachea (Sala et al., 2011), stripes appear simultaneously and so identifying leading and lagging genes would require more quantitative measurements.

It is striking that increasing component number (even at an arbitrarily chosen level) reduces constraint very rapidly. Notably, at the three-component level there is a small part of parameter space for which networks give behaviours that do not conform to the minimal topologies from which they are derived. How this grows and whether it could be important at higher orders is not clear. However, the ‘black boxing’ of whole pathways into single nodes provides a way of rationally reducing the system complexity in a way that makes biological sense. It remains an open question as to how to integrate this way of thinking with omic datasets. There is a significant body of work on analysis and simplification of complex regulatory networks and some thought has been given to RD processes within these (Mincheva and Roussel, 2012). However, practical application of these methods with experimental input is still in its infancy. Meanwhile, caution must be applied. For example, in the limb, modelling and experiments have shown that BMP2 and its transduction, manifested as phosphorylated Smad proteins, are spatially out-of-phase (Raspopovic et al., 2014). This highlights the fact that defining the activity of a pathway as expression of its direct transcriptional targets is a choice rather than a necessity.

Apart from the computational aspects of our work, this study raises biological questions. Why, for example, does the patterning of the palate use five pathways when, in principle, two would do? More specifically in our system, why is the rugal pattern initiated with three morphogens but then incorporate an additional two? One possibility is that this provides a particular type of robustness to perturbations: yes, there are many targets whose mutation can affect the pattern, but there is also significant redundancy such that the modifications to the pattern are mostly relatively subtle. Another possibility is that each pathway provides an additional tuning of the pattern or setting up of signalling for downstream events such as differentiation. A completely opposite explanation is that all of the regulatory interactions exist in cells and are used elsewhere for multiple other purposes (for RD or not, and in pairs or not), and that the apparent ‘overkill’ in terms of numbers of pathways involved is merely because there is no evolutionary pressure to eliminate or suppress their role in the palate. More detailed analysis of the robustness properties of these networks and of the conservation of the regulatory interactions is needed to address these questions.

## MATERIALS AND METHODS

### Generation of embryos and explants

Wild-type CD1 mice (*Mus musculus*) were obtained and used according to protocols approved by the Institutional Animal Ethics Committee under UK Home Office Project and Personal Animal Licences. Embryos were harvested, staged, fixed and stained for whole-mount *in situ* hybridization using established methods. Palate explants from E13.5 embryos were made using 0.1 mm tungsten needles and cultured at 37°C in 5% CO_2_ atmosphere for 24 h using the Trowell technique (Alfaqeeh and Tucker, 2013) in serum-free advanced Dulbecco's modified eagle media/F12 (GibcoBRL), 20 U/ml penicillin-streptomycin (GibcoBRL), 50 mM transferrin (Sigma-Aldrich) and 150 µg/ml ascorbic acid (Sigma-Aldrich). Chemical inhibitors were added at the beginning of the 24 h culture period at the following final concentrations: SU-5402 (Calbiochem) at 40 µM; cyclopamine (Sigma-Aldrich) at 20 µM; IWP-2 (Cambridge Bioscience/Cell Guidance Systems) at 50 µM; and dorsomorphin (Cayman Chemical) at 50 µM. Control palates were contralateral explants from the same embryo incubated with vehicle only. Experiments were repeated at least four times for each condition.

### *In situ* hybridization

For sectioned *in situ* hybridization, fixed specimens were embedded in wax and serially sectioned (7 µm), with successive sections mounted on four different slides to allow different probes to be used on nearby/adjacent sections. Whole-mount and sectioned *in situ* hybridisations were conducted according to standard methods (Economou et al., 2012). Probes were gifts from colleagues obtained initially from authors of published references as follows: *Shh* (Echelard et al., 1993); *Lef1* (Gat et al., 1998); *Id1* (Rice et al., 2000); *Spry2* (Tefft et al., 1999); *Ptch1* (Goodrich et al., 1999); *Gli1* (Hui et al., 1994); *Etv4* (*Pea3*); *Etv5* (*Erm1*) (Chotteau-Lelièvre et al., 1997); and *Axin2* (Lustig et al., 2002). Whole-stained explants, placed in a minimum volume of PBS in wells cut into 1% agarose, were digitally imaged using a stereo dissecting microscope.

### Identifying rugae in explant culture

To determine the number of rugal stripes of *Shh* expression, as well as their position, width and staining intensity, a 150 µm wide strip was drawn along the AP length of the palate through the region of the rugae. The mean greyscale level along the mediolateral axis at each AP position was determined, with rugae appearing as peaks in *Shh* intensity (note that as stronger intensity staining has a lower greyscale value, peaks in the array fall at greyscale minima). The AP boundaries of each ruga were taken at half the height from a rugal peak to the adjacent troughs. In some instances, rugae were closely spaced, leading to the fusion of rugal *Shh* peaks (as identified by the intensity of a trough being greater than one third the height of the peak to the next trough). For such fused blocks of rugae, boundaries were determined by interpolating between the outer well-identified boundaries and thresholding relative to this line. The bounds of ruga 1 were not calculated as the edge of the palate made necessary intensity measurements at the anterior unreliable. The position of each ruga was determined as the midpoint between the AP boundaries (apart from ruga 1 and 8, which were manually identified on each image), and ruga width as the difference between these boundaries. Ruga intensity was measured as the minimum greyscale value within the bounds of a ruga.

### Quantifying the effect of inhibition

Rugae in inhibitor-treated explants were aligned to their contralateral controls by minimising the sum of the squared differences between the positions of ruga 1 and ruga 8. AP position was measured relative to the control ruga 8, and each ruga was paired with the closest ruga in the contralateral shelf. In some cases, a ruga could not be paired; for example, if the closest ruga in the control lay closer to another ruga in the treated. In such cases, if the position of the ruga aligned to an interrugal region in the contralateral, it was determined that the ruga had been lost upon inhibitor treatment (or the equivalent ruga was not inserted in the contralateral). In these cases the width was recorded as 0 µm and the intensity measured at the equivalent position in the interrugal region. Alternatively, if multiple rugae in the control aligned with the same ruga in an inhibitor-treated specimen, it was determined that multiple rugae had fused. In these cases, the width of the fused-region ruga was divided in proportion with the control rugae, and the intensity was taken within the bounds of these regions. To identify any differences associated with the addition of new rugae, posterior rugae were identified as ruga 8 and the next anterior ruga.

To determine how distributions of these different measurements shifted upon inhibitor treatment, the variation in the absence of inhibitor was determined. Position, width and intensity were measured for contralateral pairs of untreated explant, and the absolute displacement from having equal values between the two was calculated. The proportion of rugae that fell above or below one median displacement from equality was calculated for each measurement under each treatment.

### Construction of target gene expression profiles

The mouse embryonic head was sectioned at 7 µm intervals in the sagittal aspect. For a given marker, *in situ* hybridization was performed on every fourth or fifth section, for the entire mediolateral extent of the rugae. Sections were imaged using a Zeiss Axioskop upright microscope with a 20× objective under both brightfield and phase contrast. To record the variation of relative expression along the AP axis of the palatal epithelium and underlying mesenchyme, the apical and basal extents of the epithelium were first manually traced on the phase contrast images (upon which unstained epithelium could be clearly seen) using ImageJ. We wrote a macro in ImageJ (see under profiles and kymographs at www.gitlab.com/adeconomou/ruga-patterning-quantifications-and-simulations) to return the relative staining intensity (as recorded in the bright-field images converted to 8-bit greyscale) within the traces along the epithelium (averaged across its thickness) and in the underlying 25 µm of mesenchyme. The basal-to-apical thickness of the epithelium was also captured. The position of ruga 3 within the trace was recorded manually. For a given palatal shelf, intensity profiles were made for all sections stained for a particular marker (sections damaged during the sectioning and staining process were excluded). Because the rugae were approximately parallel, it was sufficient to align the intensity profiles to the position of ruga 3 to obtain an average intensity profile along the AP axis of a palatal shelf (Fig. S5). Average intensity profiles were normalised, with the maximal signal intensity (darkest pixel value) taken as 1, and the minimal signal intensity (lightest pixel value) taken as 0.

### Construction of kymographs

As the palatal epithelium extends along its AP axis through localised growth immediately anterior to ruga 8, the AP palatal distance between ruga 3 and ruga 8 was used to measure embryonic age (i.e. time). Embryo weights were recorded (in 25 mg bins) and first a high-resolution calibration curve was constructed relating embryonic weights to time-of-harvest for 412 embryos across 38 litters, as described by Peterka et al. (2002). Weights and rugae 3-to-8 distances of *in situ*-hybridised embryos could then be related to embryonic age through a second calibration curve (Fig. S14). Processing to make the kymographs was carried out using R Core Team (2013) (see code at www.gitlab.com/adeconomou/ruga-patterning-quantifications-and-simulations.git) as follows. To produce a smooth kymograph, normalised intensity profiles were positioned by time and aligned in the AP direction at ruga 3. A moving average (using a 0.2 day window) was calculated to smooth the intensity plot in the time axis. Where there were fewer than two traces in the window, no value was plotted. Finally, the kymograph was replotted as distance relative to ruga 8 (see sample dataset at www.gitlab.com/adeconomou/ruga-patterning-quantifications-and-simulations). For correlation analysis, Spearman's rank correlation coefficient was calculated between the normalised intensity of *Shh* and each target across 600 evenly spaced time points from E12.5 to E14.0, for all AP positions. The onset of periodic expression was taken as the position at which the correlation coefficient reached half of its final level (measured across the anterior one third of the palate). For all AP positions, 95% confidence intervals were calculated from 1000 bootstrap replicates.

### Numerical simulations

RD systems were simulated in R using a piecewise linear model. (A detailed description of relevant code is given in Appendix S1 and the code itself is freely available from GitLab at www.gitlab.com/adeconomou/ruga-patterning-quantifications-and-simulations.git.)

The model was of the form:
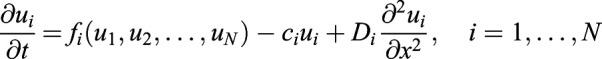

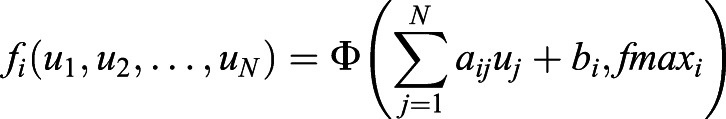
with
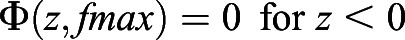

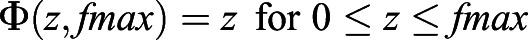

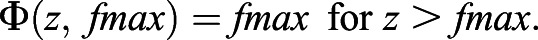


The *N* variables *u_i_*(*x*, *t*), *i*=1,…,*N*, represent the concentrations of each component of the RD system as functions of time and a single spatial variable *x* (distance along the AP axis of the palate). The non-negative parameters *c*_*i*_ and *D*_*i*_ represent the degradation rate and diffusion coefficients of the components, respectively.

The function *f_i_*(*u*_1_,…,*u_N_*), *i*=1,…,*N*, specifies the production rate of component *i*, which takes a non-negative value between 0 and *fmax_i_*. We take this to be a piecewise linear function Φ of the weighted sum of all regulatory inputs, where the parameter *a*_*ij*_ represents the weight of the direct regulatory input from component *j* to component *i* (i.e. the sensitivity of the production rate of *i* to changes in the concentration of component *j*).

The qualitative form (topology) of an RD system is specified by the nature of the interactions between its components (positive, negative or no interaction). For any given topology, interaction parameters (*a_ij_* where *i*≠*j*) were either set to 0 (i.e. no interaction) or assigned a value at random from a uniform distribution between −1 and 1, with the sign determined by the nature (positive or negative) of the interaction. For self-interactions (*a_ij_* where *i*=*j*), the composite parameter *a_ii_*−*c_i_* was drawn from this uniform distribution, taking a negative weighting when there is no autoregulation (*a_ii_*=0). When a component auto-activates, the parameter takes a positive value, with the weight of −*c_i_* also being drawn from the distribution. Diffusion coefficients (*D*_*i*_) were drawn as the reciprocal of values from a uniform distribution between 1 and 10,000, meaning that at the lower end of the distribution, small differences would have a large effect on the diffusion range.

To assess whether a particular parameterization of the RD system would support the formation of spatial patterns through DDI, and what phase-type of pattern the parameterization could produce, the parameters were compared with the criteria for a DDI generating stable spatially periodic non-oscillating patterns described initially by White and Gilligan (1998) with some additional elaboration described in Appendix S1, section 1.

To ensure that spatially periodic solutions of the model do not take negative values, they must form around a positive spatially-uniform steady state (all *u_i_*>0); to ensure this, appropriate production constants *b*_*i*_ are required (Raspopovic et al., 2014). Therefore, for parameter sets that support DDI, constant regulatory input terms (*b*_*i*_) were calculated so that for each component, the spatially-uniform steady state concentration was fixed at 1. In order for the amplitude of patterns generated from linear RD to remain bounded, the production functions *f_i_*(*u*_1_,…,*u_N_*) must be bounded (Shoji et al., 2003). We set the lower bound to be zero (production cannot be negative) and the upper bounds to be *fmax*_*i*_. To ensure that the maximum production rates *fmax*_*i*_ were greater than the production rate at steady state (so that the production rates at steady state are linear functions of their inputs), the *fmax*_*i*_ were set randomly between 1.5× and 3× the magnitude of *c*_*i*_.

RD simulations were run using a finite difference scheme with zero flux boundary conditions on a discrete one-dimensional grid of 100 positions. Parameter sets were scaled to ensure non-discretised spatially periodic solutions would be formed on the spatial domain used by comparing the wavelength and growth rate (as detailed in Appendix S1, section 1) with values known to fit within the simulation space and time to generate a scaling factor, and then using that factor to run an initial simulation from which spatial patterns could be measured and the parameters scaled more precisely.

### Perturbation analysis

Scaled parameter sets were simulated as described above, with all grid positions initially drawn at random from a uniform distribution between −0.01 and 0.01 around the equilibrium point of the reaction term matrix of the RD system. For parameterisations in which a stable spatial pattern of 2 to 4.5 complete waves (to avoid discretisation) was established in the RD system, components were inhibited in one of two different ways. Inhibition of the receptor of a given morphogen (as seen for the inhibitors cyclopamine, SU-5402 and dorsomorphin, for example) was implemented as an equal proportional reduction of all interaction coefficients representing the response to that morphogen [so if the response to component *u*_*j*_ was inhibited, the values of *a*_*ij*_ were reduced by a factor (1–*α*) for all values of *i*]. Inhibition of the production of a component (e.g. IWP-2) was achieved by proportionately reducing the production term of that component by a factor (1–*α*) (see Appendix S1, section 2 for details). For all parameter sets, by successively reducing the strength of the inhibition parameter *α* by a factor of 0.25 from complete inhibition (*α*=1), we determined empirically for each pathway the maximum perturbation *α*_*max*_ that still allowed patterning but did not perturb the number of whole wavelengths or fail to achieve a stable amplitude. The test statistic for perturbation effects was the mean level for each component relative to an unperturbed run. To confirm that this readout accurately reflected an upward or downward shift in the wave, as was seen in the experimental system, the shift in the troughs and peaks of the waves, respectively, were also recorded: if an increase in mean was associated with an increase in the position of the wave troughs, or a decrease in the position of the wave peaks, it was determined that the wave had shifted upwards or downwards, respectively (Fig. S8F,G).

### Topology search

For the three-component RD system, parameter space was systematically sampled to identify parameter sets and topologies capable of giving DDI. Each of the nine reaction parameters *a*_*ij*_ were varied linearly through seven values spread evenly around 0 to give a total of 40,353,607 parameter sets. Diffusion parameters were initially set as two fast and one slow or vice versa, although this requirement [based on the longstanding but recently overturned idea (Marcon et al., 2016) that this difference is essential] turned out not to be essential (see Results). DDI criteria for stable periodic non-oscillating patterns, as mentioned above and described in detail in Appendix S1, section 2, were applied.

## Supplementary Material

Supplementary information

Reviewer comments
