# Supplementary material for: Perturbation analysis of a multi-morphogen Turing reaction-diffusion stripe patterning system reveals key regulatory interactions
Source: Development. 2020 Oct 29;147(20):dev190553. doi: 10.1242/dev.190553 (PMC7648603; doi:10.1242/dev.190553)
Supplement: Supplementary information [file develop-147-190553-s1.pdf]

Supplementary Figures and Tables for Economou et al. " Perturbation analysis of a multi-morphogen Turing Reaction-Diffusion stripe patterning system reveals key regulatory interactions"

This document contains

- Supplementary Figures S1-S16 (pages 1-10)
- Supplementary Tables 1 and 2 (page 11)
- Supplementary Notes 1-4 (pages 12-45)

Figure S1: Efficacy controls for inhibition of Wnt and BMP signalling In situ hybridisations on E13.5 palatal shelf explants for Wnt target gene Lef1 and BMP target gene Id1, cultured for 24 hrs in the small molecule inhibitor IWP-2 and dorsomorphin respectively. Contralateral shelves shown as vehicle controls. Well known domains of target gene expression in palatal region are lost upon inhibitor treatment; see loss of strong Lef1 expression in tooth upon IWP-2 treatment, and loss of strong Id1 staining at medial palate edge (see arrows). Darker regions in inhibitor treated specimens represent background staining in areas of thickened tissue. Anterior to the right, medial up. Scalebar = 200 µm.

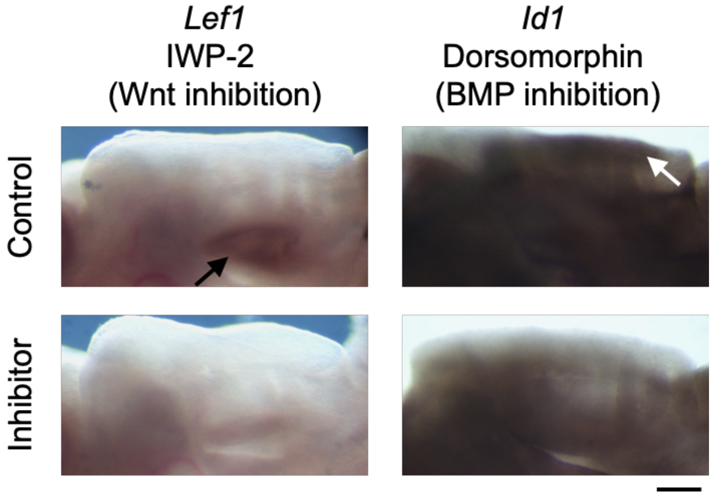

Figure S2: Identifying rugae from *Shh* in situ hybridisation of whole mount explant cultures

a) *Shh* in situ hybridisations on E13.5 palatal shelf explants cultured for 24 hrs. Red rectangle shows 150 µm band along which *Shh* staining intensity was measured. Anterior to right, medial up. Scalebar = 200 µm.

b) Plot of mean *Shh* staining intensity for each AP position along the red rectangle in a), against the AP position relative to the posterior of the image. Blue dots and coarse dashed lines denote positions of ruga 8 and ruga 1, identified manually on specimen in a). Green and red dots denote half heights and one third heights respectively for each peak, as illustrated by fine dashed lines on anterior of ruga 2. Arrows denote the next position in the array with the same intensity as marked by the red dots, with red arrows identifying potentially fused rugae.

c) Plot of mean *Shh* staining intensity as in b) with blue bars denoting the positions of the rugae, with anterior and posterior boundaries determined by the blue dashed lines. Black dashed lines denote position of rugae, taken as the manually identified positions for ruga 8 and ruga 1, and the ruga midpoints for the remaining rugae.

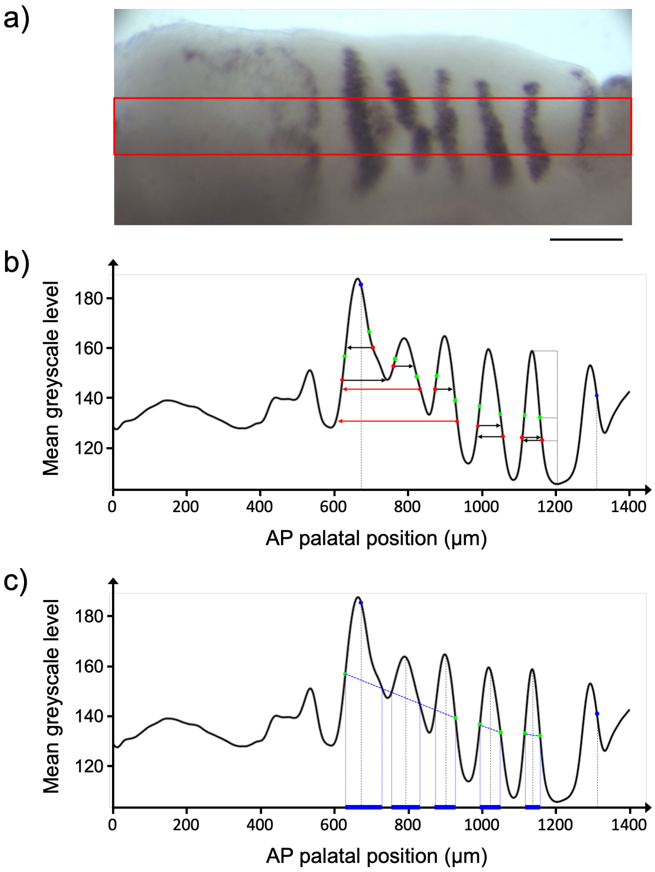

Development • Supplementary information

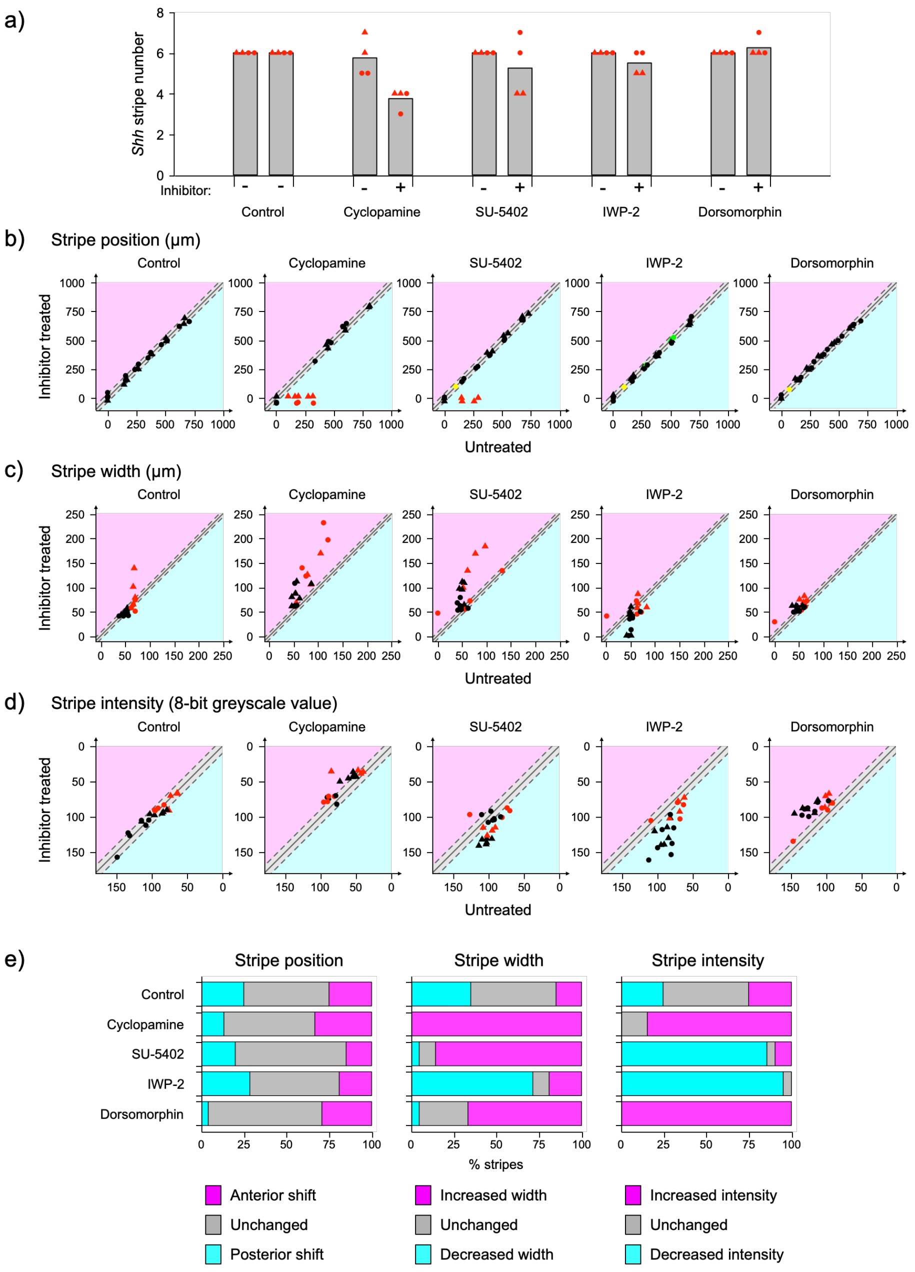

Figure S3: Effects of pathway inhibition on the pattern of the rugae

a) Mean number of rugae as marked by clear peaks of *Shh* expression in E13.5 palatal shelves cultured for 24 hrs with the specified inhibitor (+) or vehicle (-).

b-d) Plots of *Shh* stripe position b), width c) and stripe intensity d) for all ruga from explants for specified inhibitor treatment, plotted against values for the equivalent ruga in stage matched untreated (vehicle) control (see Methods for details of quantifications). Plots from paired untreated explants are also shown to show levels of variability in the absence of inhibitor. Solid grey lines denote equal values, dashed lines represent one median displacement around the line of equal values for untreated explants (see Methods for details). Magenta regions represent larger, and cyan regions smaller, values in inhibitor treated explants. For stripe position b), red points relate to rugae determined to have fused with another ruga in the inhibitor treated explants, with their position in plotted against the position of that ruga in the inhibitor treated explant. Yellow and green points relate to rugae in the inhibitor treated and untreated explants respectively, where the equivalent ruga could not be identified in the stage matched explant (respectively the untreated or inhibitor treated). For measures of stripe width c) and intensity d) points in red denote posterior rugae (see Methods).

e) Percentage of rugae for each treatment lying within one median displacement from the line of equal values (grey), or above (magenta) and below (cyan), for each of the three measures in b-d).

All plots depict quantifications from four pairs of explants. Drug treatments were carried out at the doses specified in the Methods, plus an additional dose, with points pertaining to lower doses shown as circles and higher dose as triangles in all plots. Doses used are: cyclopamine 20 μM and 80 μM, SU-5402 40 μM and 80 μM, IWP-2 10 μM and 50 μM, dorsomorphin 10 μM and 50 μM.

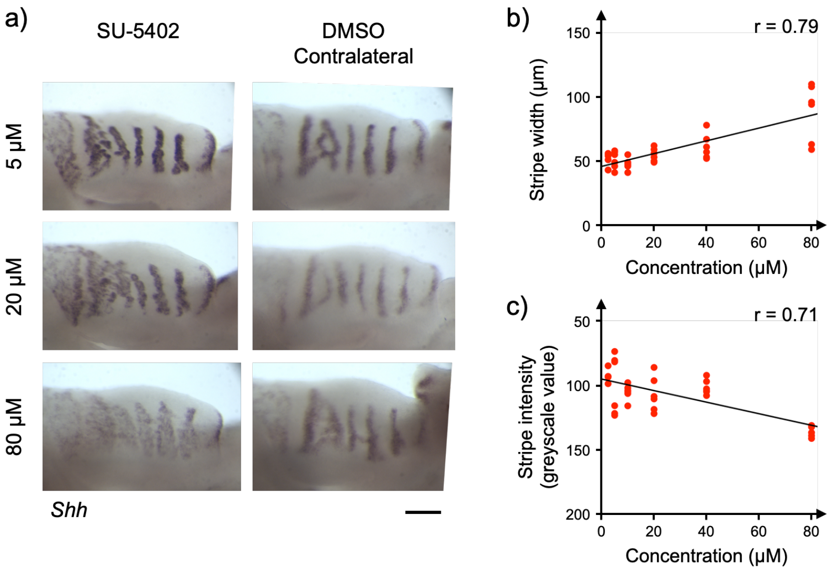

Figure S4: Titration of FGF inhibitor SU-5402

a) *Shh* expression in palatal shelves explanted at E13.5 and cultured with the FGF receptor inhibitor SU-5402 for 24 hrs at the indicated doses, alongside the contralateral shelves which were cultured in DMSO.
Scalebar = 200 µm.
b) Plot showing positive correlation between concentration of SU-5402 and *Shh* stripe width.
c) Plot showing positive correlation between concentration of SU-5402 and *Shh* stripe greyscale intensity. Note that as more intense staining has a lower greyscale value, greyscale axis is plotted in reverse to show a decrease in staining intensity with increased concentration of inhibitor.
Points in b) and c) depict ruga 4 to 2 from two embryos per concentration. Other rugae are not plotted as measurements were not calculated for ruga 1 (see methods), while more posterior rugae appear very variable even in control treatments (see Results). Pearson's product-moment correlation coefficient (r) is given for each plot in b) and c).

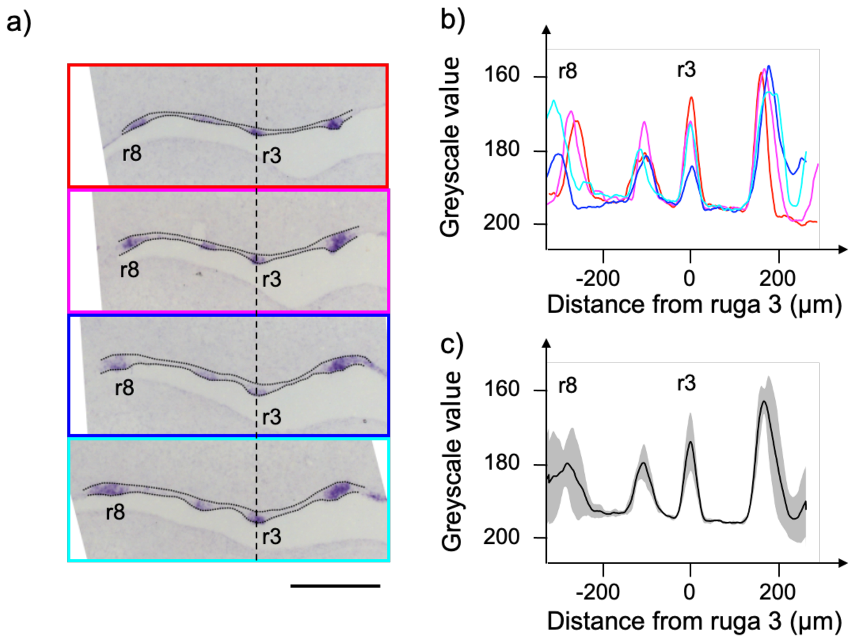

Figure S5: Quantification of striped rugal expression

a) In situ hybridisation showing Shh expression in sagittal sections taken at 35 µm increments across a E13.5 palatal shelf, ordered from medial most (red outline) to lateral most (cyan outline). The positions of ruga 3 and ruga 8 are indicated. Dotted lines illustrate the extent of the palatal epithelium used for quantifications. Anterior to right.
Scalebar = 200 µm.
b) Intensity profiles for each section aligned by the position of ruga 3. Peaks in Shh intensity at ruga 3 and ruga 8 are indicated. Profiles coloured as in a).
c) Mean intensity profile from the four sections in a) and b). Shaded area represents 1 +/-sd.

Figure S6: Additional pathway marker quantification
In situ hybridisation of sagittal section through E13.5 palatal shelf for additional markers of pathway activity. Dotted lines illustrate the extent of the palatal epithelium and the underlying mesenchyme used for quantifications. Anterior to right. The intensity profile averaged across the palatal shelf shown for each specimen from which illustrated in situ is taken for gene of interest (coloured trace) and *Shh* (grey trace) for the epithelium and mesenchyme. Shaded areas represent 1 sd around gene of interest (for clarity of presentation, variation around *Shh* trace is not shown). For each marker, the number of specimens showing the observed pattern are: *Ptc1* 41, *Erm* 38, *Spry2* 36 and *Axin2* 43. Scalebars = 200 μm.

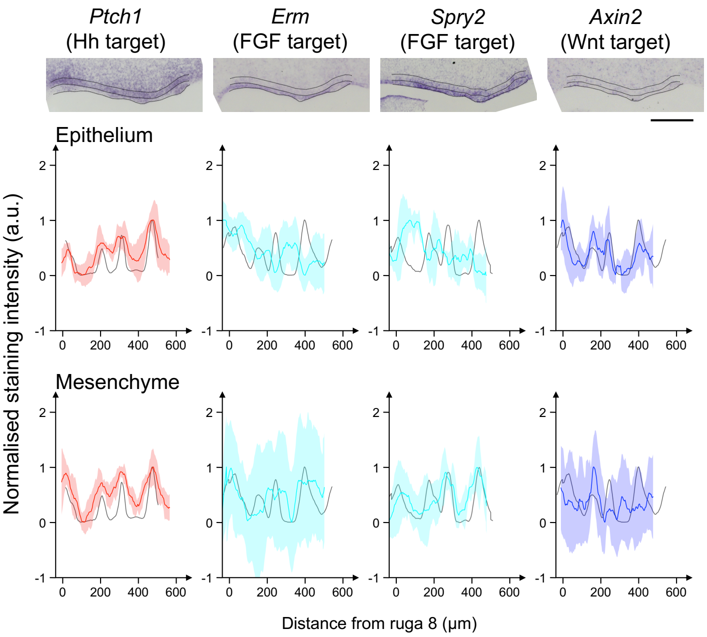

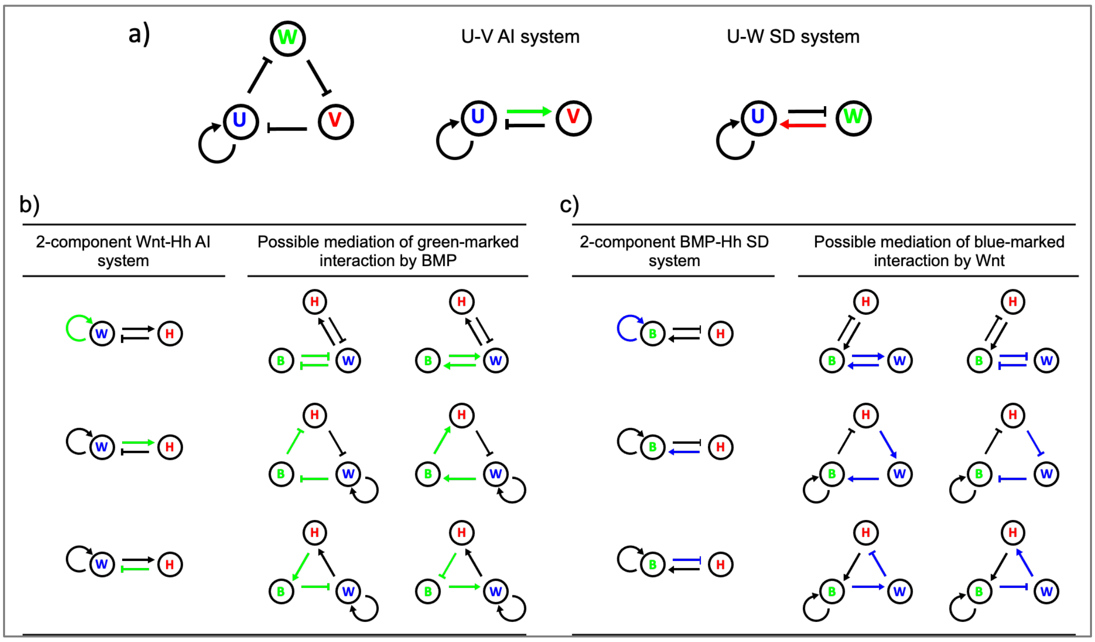

Figure S7: Adding a component as a mediator of an interaction
a) Network topology comprising three components U, V and W. This topology can be interpreted as a two-component activator-inhibitor system between 'master morphogens' U and V, where the positive interaction from U to V is 'mediated' by a double inhibition (ie a positive interaction) through W (see green arrow in U-V AI system). Alternatively, this same three-component topology can be interpreted in a similar manner as a substrate -depletion system with 'master morphogens' U and W, where V 'mediates' the positive interaction from W to U (see red arrow in U-W SD system).
b) All possible ways that an interaction in a two-component Wnt-Hh AI network (marked in green) can be 'mediated' by BMP in the adjacent three-component Wnt-BMP-Hh RD networks. Interaction in the three-component RD networks maintain the net sign of the marker interactions in the two-component networks; the phase of BMP relative to Wnt and Hh is not considered.
c) Same as in b) for Wnt mediating interaction in a two-component BMP-Hh SD network (with interactions 'mediated' by Wnt in blue).

Development • Supplementary information

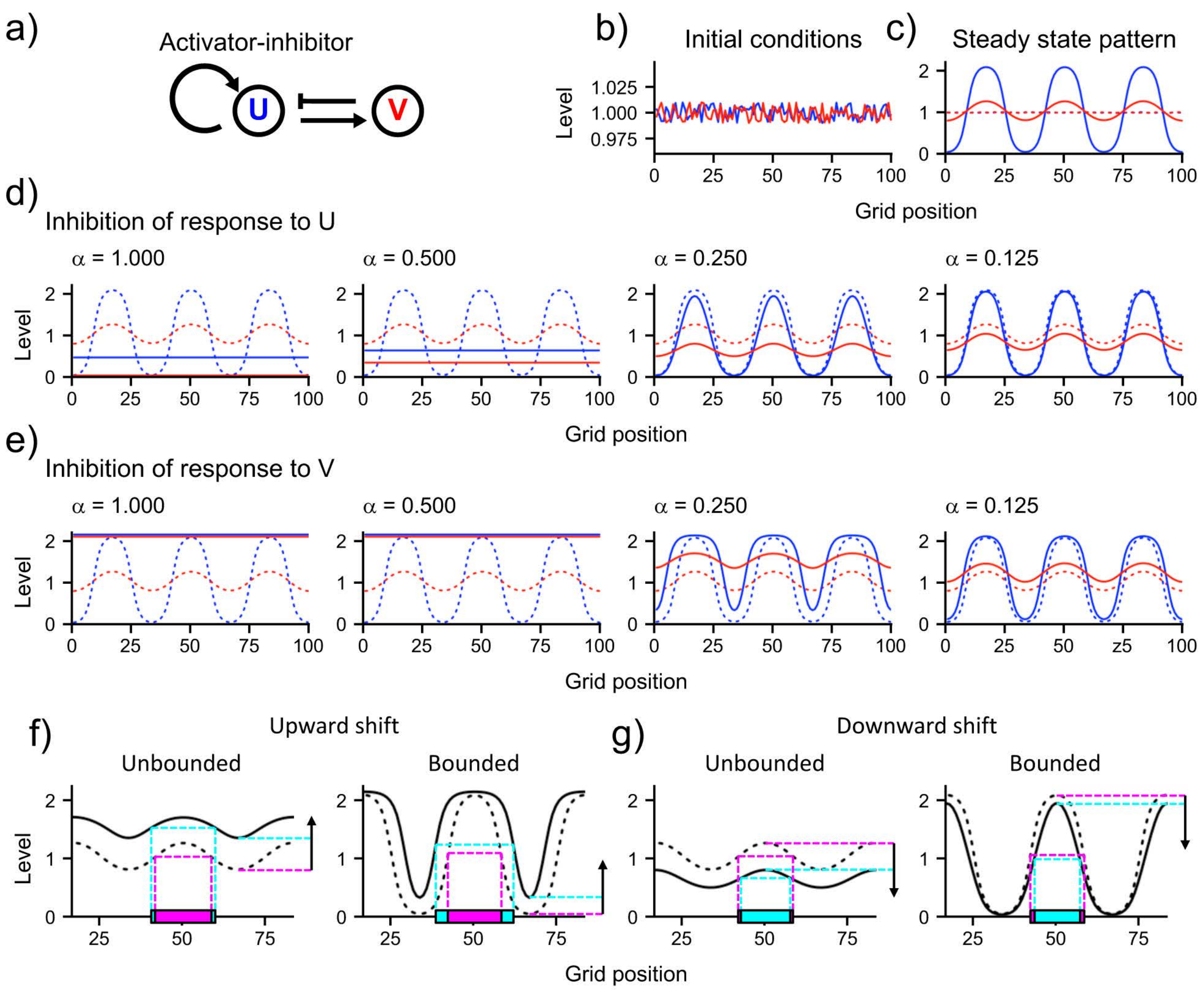

Figure S8: Illustrative simulation of two-component reaction-diffusion system show response of waves to inhibition of components

a) Two-component activator-inhibitor topology used for illustrative simulations. Simulations carried out as detailed in methods with u_1 is component U and u_2 is component V, with a_{11} = 0.017, a_{12} = -0.013, a_{21} = 0.016, a_{22} = 0.000, b_1 = 0.005, b_2 = 0.001, c_1 = 0.009, c_2 = 0.016, $fmax_1$ = 0.019, $fmax_2$ = 0.035, D_1 = 0.63 and D_2 = 24.17.

b) Illustration of initial conditions of U and V as described in Methods.

c) Steady state pattern of waves of U and V after simulation from the initial conditions in b). Initial conditions also shown as dashed lines (note difference in scale between b) and c).

d-e) Effect on steady state waves in c) of subsequent inhibition of response to U and V respectively for decreasing strengths of inhibition (values of α – see Methods). For comparison dashed lines show waves of U and V in the absence of inhibition. For b-e) component U is shown in blue, and component V in red.

f-g) Illustrative examples of the effects of inhibitions on waves, with solid waves showing shape after inhibition and dashed waves the uninhibited shape. Waves can shift up (as in f), either in an unbounded or bounded manner. In the latter case only troughs noticeably shift upwards with little effect on the position of peaks (see arrows to the right of plots showing difference in positions of inhibited (cyan) and uninhibited (magenta) troughs. Alternatively, waves can shift down (as in g), either in an unbounded or bounded manner. In the latter case only peaks noticeably shift downwards with little effect on the position of troughs (see arrows to the right of plots showing difference in positions of inhibited (cyan) and uninhibited (magenta) peak. The bounded condition in particular, can show a change in wave width, with an increase when the wave shifts up (see f) and a decrease when it shifts down (see g) as determined by the width of a wave taken at half the height from trough to peak (see dashed cyan (for inhibited) and magenta (for uninhibited) lines and bars).

Development • Supplementary information

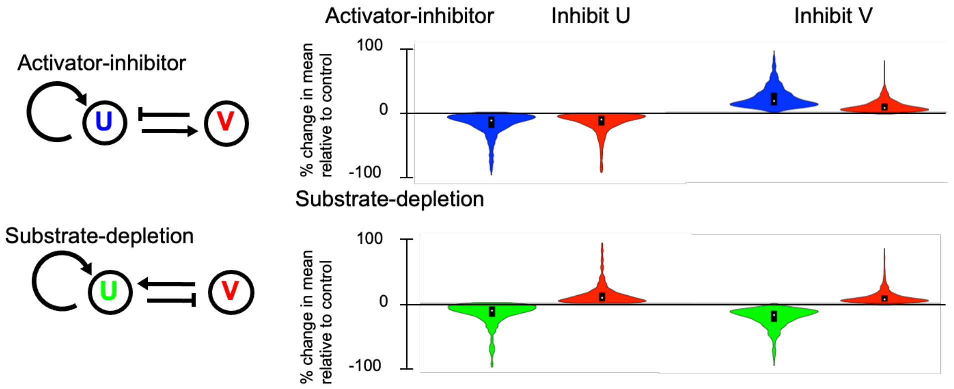

Figure S9: Inhibition of production produces the same outcomes as inhibition of response
Violin plots showing percentage change in the mean level of components U and V in illustrated activator-inhibitor (AI) and substrate-depletion (SD) RD networks, on inhibition of the production of component U and V in RD simulations.

a)

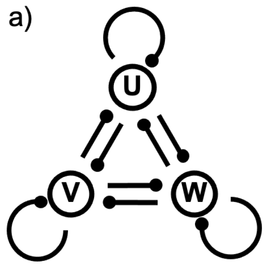

b)

All possible topologies:	**19683**
Degradation by default:	**5832**
Stable DDI topologies:	**1492**
Strongly connected:	**1396**
For each phase relationship:	**498**
Minimal topologies (per phase):	**45**

Figure S10: Three-component topologies and exclusion by successive constraints
a) Three-component network topology showing the nine possible interactions captured in the reaction matrix.
b) Enumeration of topologies recovered from parameter search under different constraints as detailed in text.

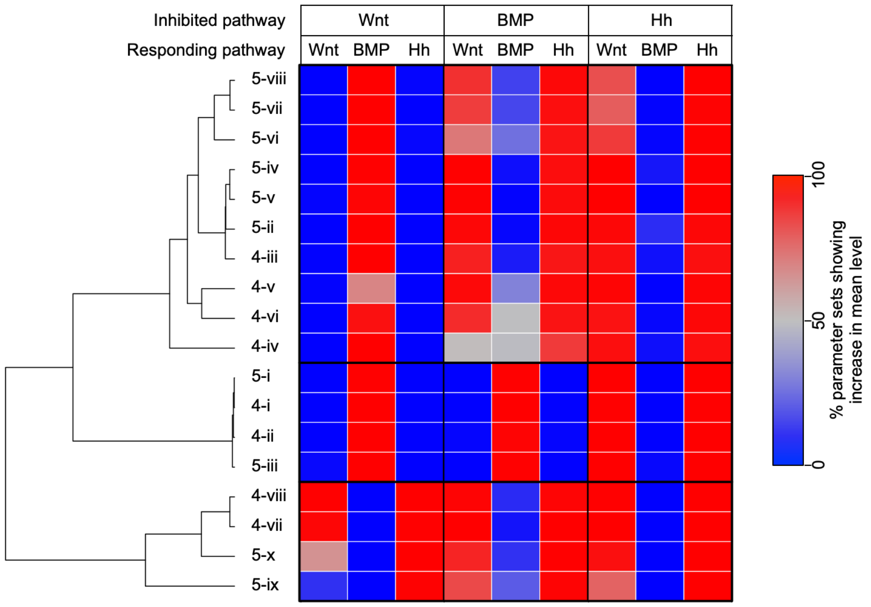

Figure S11: Responses of three-component networks to perturbation
Full heat map showing the percentage of parameter sets where the level of each component increases in response to the inhibition of each component in the network in RD simulations. Topology names are as in figure 3. Hierarchical clustering shows that the behaviours of the topologies fall into the main categories for which all three components show similar behaviour.

Development • Supplementary information

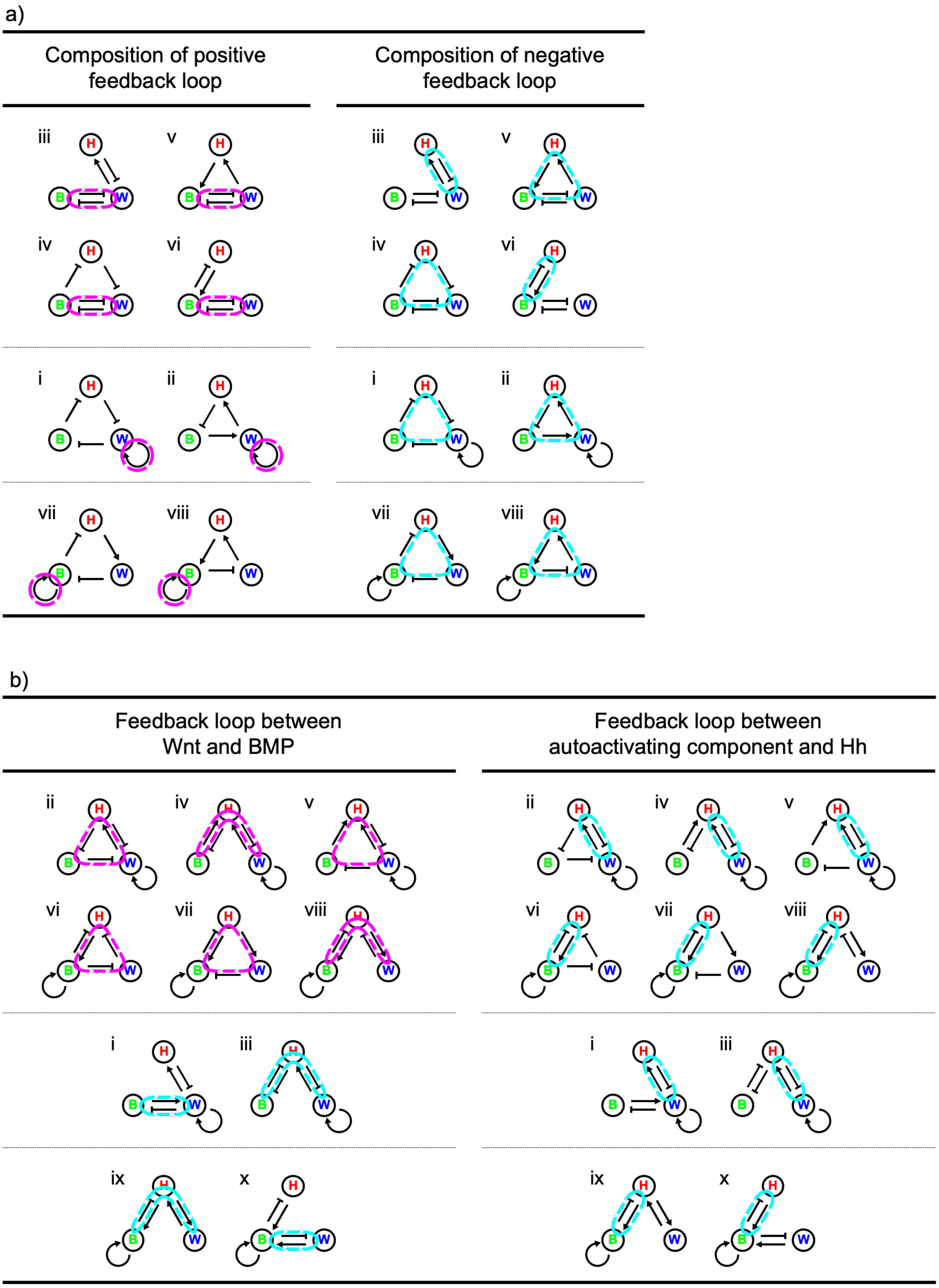

Figure S12: Feedback loops in three-component networks

a) Schematics showing the eight minimal 4-interaction RD networks recovered from the parameter search, with either the single positive feedback loop marked in magenta (left) or the single negative feedback loop marked in cyan (right). Topologies are group as according the the hierarchical clustering in figure S7.

b) Schematics showing the ten minimal 5-interaction topologies networks recovered from the parameter search. On left, the net feedback between Wnt and BMP is marked, with magenta for a positive feedback, or cyan for a negative feedback. (It should be noted that for some topologies (such as iv, vii, iii and ix) the feedback is indirect with both the interaction from Wnt to BMP and BMP to Wnt pass through Hh. For example, in iv, while there is no single positive feedback loop, the net interaction between Wnt and BMP is a mutual inhibition and therefore a net positive feedback.) On the right, the presence of a negative feedback between Hh and the autoactivating component (Wnt or BMP) is marked in cyan. Topologies are group as according the the hierarchical clustering in figure S7.

Wnt in blue, BMP in green and Hh in red.

Development • Supplementary information

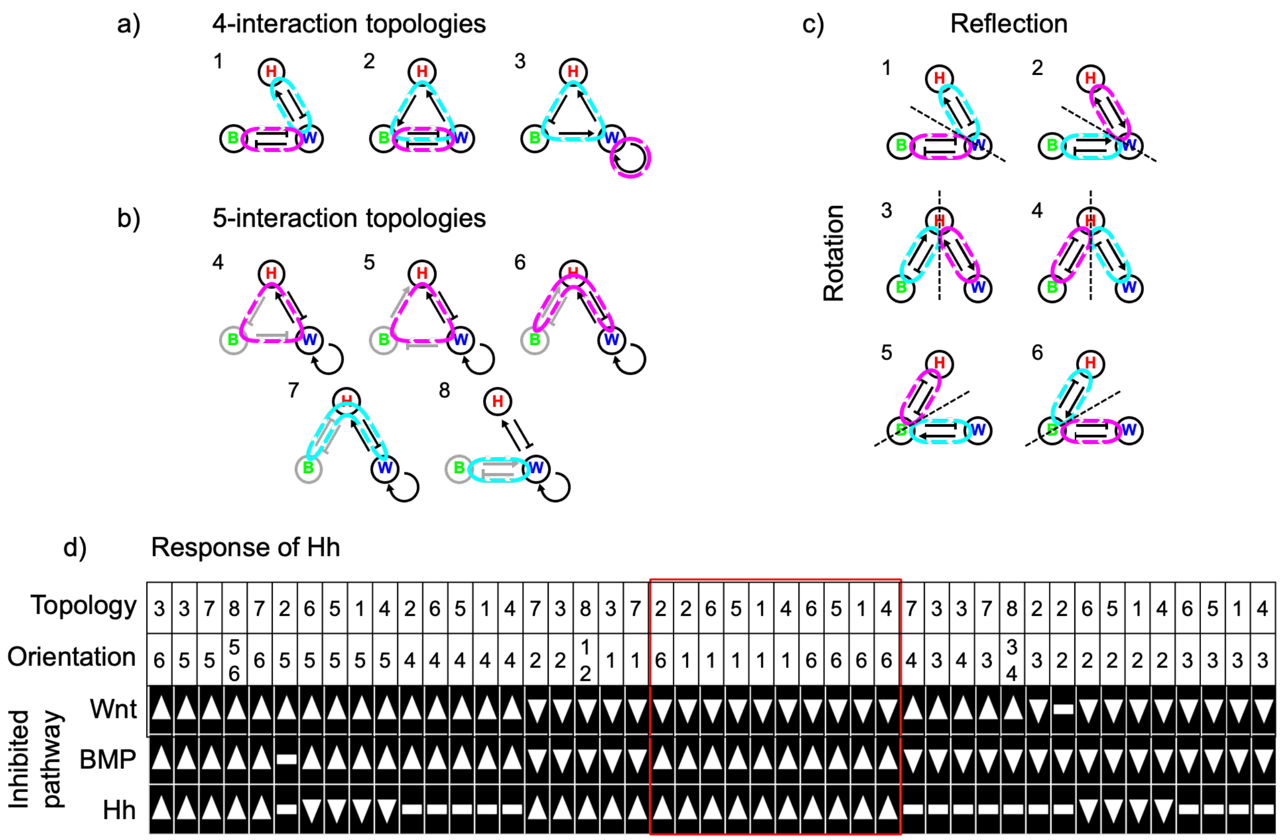

Figure S13: Using topology to constrain the response to perturbation of minimal three-component networks
a) Illustrative examples of minimal 4-interaction three-component RD networks showing the three different ways a single positive and negative feedback loop can be combined. The positive feedback loop can pass through one (topology 3) or two (topologies 1 and 2) components, and the negative feedback can pass through two (topology 1) or three (topologies 2 and 3) components. Positive feedback loops in magenta, negative feedback loops in cyan.
b) Illustrative examples of 5-interaction three-component RD networks showing the five different ways that an external component can be wired into a two-component RD core, such that the new topology is not an elaboration of one of the minimal architectures in a). Core components and interactions in black, with external in grey. The nature of the net feedback between the external component (BMP in the illustrative examples) and the core positive feedback component (Wnt in the illustrative examples) is shown as a magenta loop for positive feedback and a cyan loop for negative feedback.
c) Examples of three-component RD networks showing the six different orientations that a set of 3-component RD loops can take, using the 4-interaction minimal architecture in a) 1 as an illustrative example. The feedback loops can be rotated into three different orientations (1, 3 and 5), as shown by the positions of the two feedback loops relative to the dashed black line. In each orientation, the architectures can be reflected around the dashed black line to give a further three orientations (2,4 and 6).
d) Response of Hh to inhibition of Wnt, BMP and Hh for all 45 possible minimal three-component Wnt-BMP-Hh topologies based on reaction term analysis. Topologies are specified by the combination of a topology (1 to 8 from a) and b)) and an orientation (1 to 6 from c)). Topology architecture 8 is symmetrical upon reflection and therefore two the topology under two possible orientations is the same. The direction of response of Hh shown by arrowhead, with up arrow indicating increase and down arrow decrease. Where the response is unconstrained a white bar is shown. Coupling between components (see supplementary note 4 and figure 4) is not shown. The ten topologies for which the predicted responses are consistent with experimental observations are marked in red. These are the same topologies as recovered by the RD analysis (see figure 3).

Development • Supplementary information

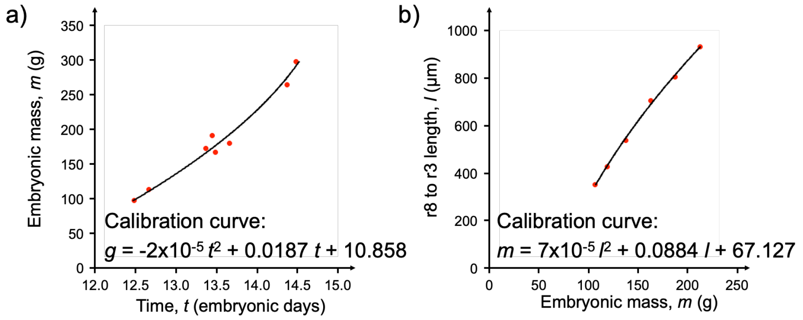

Figure S14: Calibration curves for converting palatal length into time

a) Plot of time litters of mice were collected vs. the mean embryo mass for that time (n = 3 litters per time point).

b) Plot of embryonic mass (taken as the midpoint of the bin) against average ruga 3 to ruga 8 length the the weight bin.

For a) and b), equations of best fit quadratics are given which allowing the conversion of palatal lengths to time in embryonic days.

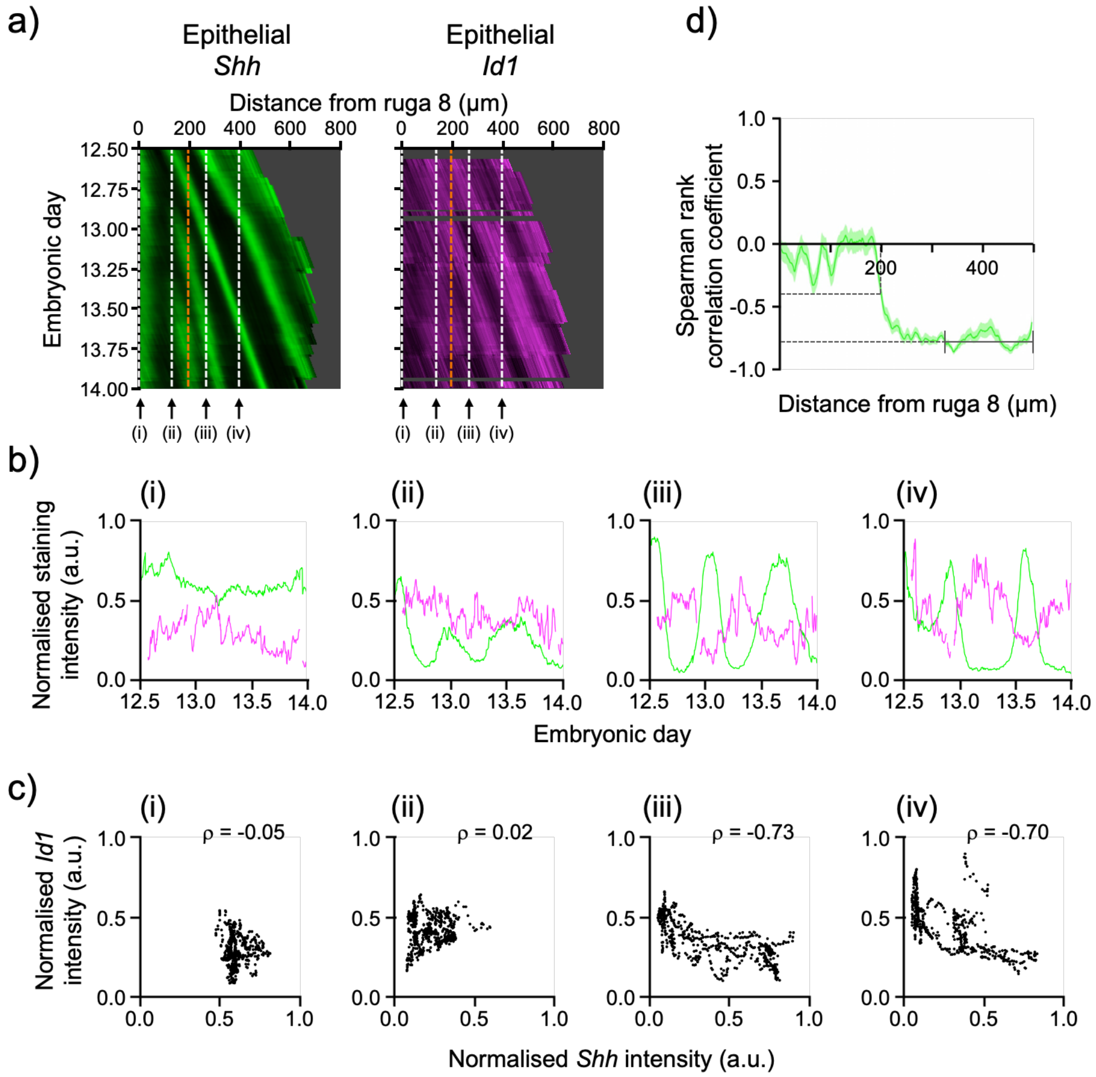

Figure S15: Illustrative example of how the appearance of periodic target gene expression relative to the onset of rugal *Shh* expression is identified

a) Kymographs of epithelial Shh (green) and epithelial Id1 (magenta) as in figure 6a.

b) Plots of normalised staining intensity for *Shh* (green) and Id1 (magenta) against time in embryonic days at four different distances from ruga 8 (i-iv), as marked by arrows and white dashed lines in a).

c) Plots of normalised staining intensity for *Shh* against Id1 for 600 equal time steps from 12.5 to 14.0 embryonic days at four different distances from ruga 8 (i-iv), as in b). The Spearman rank correlation coefficient (ρ) is given for each distance.

d) Plot of Spearman rank correlation coefficient (ρ) against distance from ruga 8 for Id1. Shaded area represents 95 % confidence interval from bootstrapping (see Methods). Solid line represents mean correlation coefficient calculate over anterior third of palatal tissue. Dashed lines demonstrate how the position relative to ruga 8 where half this value is reached is used to determine the onset of out-of-phase Id1 expression, marked by orange dashed lines in a).

Development • Supplementary information

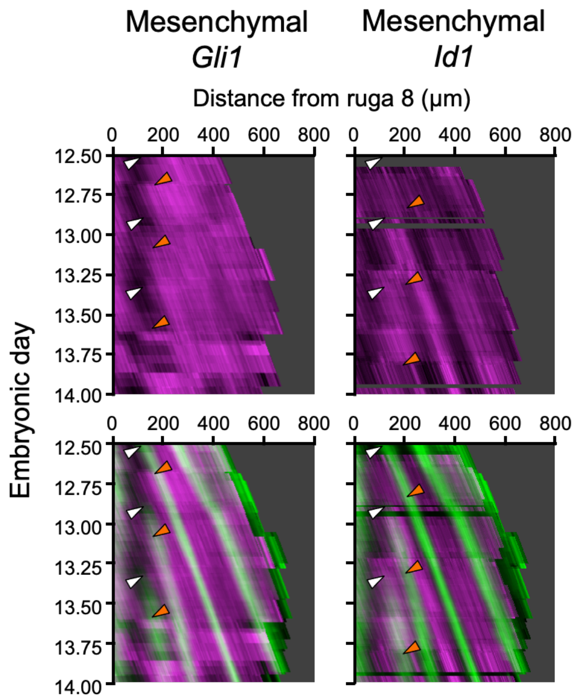

Figure S16: Kymographs showing patterns of mesenchymal expression through time for *Gli1* and *Id1*
Kymographs showing the pattern of expression of mesenchymal *Gli1* and *Id1* through time (magenta) and their expression relative to *Shh* (green) for rugae 3, 4 and 5. White arrowheads indicate the approximate onset of *Shh* expression for each ruga, and orange arrowheads indicate approximate positions of the change in the expression pattern of each target gene associated with each ruga.

Development • Supplementary information

Supplementary Tables:

Topology	Steady state (%)	Maintain wave number (%)	Shift in wave position (%)			
			Inhibit U		Inhibit V	
			U	V	U	V
AI (R)	95.2	100.0	99.8	100.0	100.0	100.0
SD (R)	89.6	100.0	99.6	100.0	99.9	100.0
AI (P)	95.2	100.0	99.9	100.0	99.4	100.0
SD (P)	89.5	100.0	99.8	100.0	100.0	100.0

Table S1: Behaviour of simulations for two-component RD topologies.
Table showing for each topology what percentage of 1000 randomly generated parameter sets consistent with the criteria for DDI reached a steady state after 1000 time steps, and what percentage of these maintained their wave number after inhibition (there is no change or loss of periodicity). For parameter sets maintaining wave number, the proportion of waves showing a shift in position (either upwards or downwards – see Methods) is given for both components of the inhibition of each component. For the two two-component network topologies activator-inhibitor (AI) and substrate-depletion (SD) both the inhibition of the response to each component (R) and the inhibition of their production (P) were considered.

Topology	Steady state (%)	Maintain wave number (%)	Shift in wave position (%)								
			Inhibit W			Inhibit B			Inhibit H		
			W	B	H	W	B	H	W	B	H
4-i	85.5	99.8	99.6	99.8	100.0	99.8	100.0	99.9	100.0	100.0	100.0
4-ii	93.2	99.7	99.9	100.0	100.0	99.9	100.0	100.0	99.9	100.0	99.9
4-iii	80.1	99.1	99.7	99.9	99.9	100.0	99.7	100.0	94.0	99.5	99.9
4-iv	89.5	98.0	99.9	99.9	100.0	99.8	99.5	100.0	90.5	99.7	100.0
4-v	92.5	99.9	100.0	85.9	100.0	100.0	82.9	100.0	98.6	95.4	99.8
4-vi	83.9	99.9	99.8	85.9	100.0	99.8	86.6	100.0	98.0	94.9	99.9
4-vii	82.6	100.0	99.4	99.9	100.0	100.0	99.8	100.0	100.0	100.0	100.0
4-viii	82.6	100.0	94.9	99.9	100.0	99.5	99.8	100.0	99.3	99.9	100.0
5-i	92.7	99.9	100.0	100.0	100.0	99.6	99.8	100.0	99.9	100.0	99.9
5-ii	90.5	99.9	99.7	100.0	100.0	99.7	99.9	100.0	99.8	99.8	99.9
5-iii	94.3	100.0	99.8	100.0	100.0	99.8	100.0	100.0	99.7	100.0	100.0
5-iv	92.7	100.0	100.0	100.0	100.0	99.7	99.4	99.4	99.7	99.9	100.0
5-v	94.6	100.0	99.8	99.9	100.0	99.6	99.8	99.0	99.5	99.8	100.0
5-vi	86.4	99.9	100.0	99.3	99.0	99.4	99.7	100.0	93.6	99.5	99.9
5-vii	73.1	99.9	100.0	99.6	100.0	100.0	99.6	100.0	99.7	99.7	99.7
5-viii	92.0	100.0	98.7	99.5	99.9	99.6	99.5	99.8	97.3	99.6	99.7
5-ix	80.6	100.0	100.0	99.0	99.9	100.0	99.8	100.0	99.8	99.6	100.0
5-x	87.0	99.7	99.5	99.0	99.4	99.7	99.6	100.0	99.7	99.7	100.0

Table S2: Behaviour of simulations for three-component RD topologies.
Table showing for each topology (as defined in figure 3b) the behavior of RD simulation of the three components Wnt (W), BMP (B) and Hh (H), as detailed in supplementary table S1. As stated in Methods, for Wnt inhibition is at the level of production, while for BMP and Hh inhibition is at the level of response.

Appendix S1. Supplementary Notes for Economou et al. " Perturbation analysis of a multi-morphogen Turing Reaction-Diffusion stripe patterning system reveals key regulatory interactions"

1: Review of Conditions for DDI in 2- 3- and *N*-component systems

The conditions for a diffusion driven instability (DDI) have been extensively analysed. For completeness, we present general conditions for a DDI, as well as specific criteria for assessing whether specific parameterisations of two- and three-component RD systems will give a DDI.

1.1: General conditions for a DDI

We considered a general reaction-diffusion system of the form

$$\frac{\partial \boldsymbol{u}}{\partial t} = \boldsymbol{f}(\boldsymbol{u}) + \boldsymbol{D}\nabla^2\boldsymbol{u},$$

where $\boldsymbol{u}$ is a vector of N reactant concentrations and $\boldsymbol{D}$ is a diagonal N×N matrix of diffusion coefficients where $N \geq 2$, such that

$$\boldsymbol{u} = \begin{pmatrix} u_1 \\ u_2 \\ \vdots \\ u_N \end{pmatrix} \text{ and } \boldsymbol{D} = \begin{pmatrix} D_1 & 0 & \cdots & 0 \\ 0 & D_2 & \cdots & 0 \\ \vdots & \vdots & \ddots & \vdots \\ 0 & 0 & \cdots & D_N \end{pmatrix}.$$

Following the well-established linear stability approach to deriving conditions for DDI, as outlined Murray (2003) (see also White and Gilligan 1998, Marcon et al. 2016) (N.B. References below), we derive the stability matrix $\boldsymbol{S}$, for which

$$\boldsymbol{S} = \boldsymbol{J} - \boldsymbol{D}q^2,$$

where q is the wave number of a spatially-periodic perturbation, and $\boldsymbol{J}$ is the Jacobian matrix of the reaction system, where the elements J_{ij} are the first partial derivatives of the components of $\boldsymbol{f}$, evaluated at a spatially-uniform steady state, such that

$$\boldsymbol{J} = \begin{pmatrix} J_{11} & J_{12} & \cdots & J_{1N} \\ J_{21} & J_{22} & \cdots & J_{2N} \\ \vdots & \vdots & \ddots & \vdots \\ J_{N1} & J_{N2} & \cdots & J_{NN} \end{pmatrix}, \quad J_{ij} = \frac{\partial f_i(u_1, u_2, \ldots, u_N)}{\partial u_j}.$$

Development • Supplementary information

Solving the system

$$\det[\lambda \boldsymbol{I} - \boldsymbol{S}] = 0$$

gives the dispersion relation for the eigenvalues λ of $\boldsymbol{S}$:

$$\lambda^N + a_1(q^2)\lambda^{N-1} + a_2(q^2)\lambda^{N-2} + \cdots + a_N(q^2) = 0.$$

For diffusion driven instability, the system must be stable without diffusion and unstable with diffusion. Thus, when q=0, all solutions λ of the dispersion relation must have negative real part, and there must exist positive values of q^2 for which there is at least one solution λ with positive real part.

We will now consider the specific cases of two- and three-component RD systems.

1.2: Two-component system

For a two-component system of the form above, the dispersion relationship takes the form

$$\lambda^2 + a_1(q^2)\lambda + a_2(q^2) = 0,$$

with coefficients

$$a_1(q^2) = -(J_{11} + J_{22}) + q^2(D_1 + D_2),$$

$$a_2(q^2) = (J_{11}J_{22} - J_{12}J_{21}) - q^2(J_{11}D_2 + J_{22}D_1).$$

Following the approach of Murray (2003) gives the conditions for a DDI. Stability in the absence of diffusion requires

$$J_{11} + J_{22} < 0,$$

Development • Supplementary information

and

$J_{11}J_{22} - J_{12}J_{21} > 0,$

and instability with diffusion requires

$$J_{11}D_2 + J_{22}D_1 > 2\sqrt{D_1 D_2 (J_{11}J_{22} - J_{12}J_{21})} > 0.$$

As is well established (see Murray 2003), if J_{11}>0 and J_{22}<0, the two components will be in-phase when J_{12}<0 and J_{21}>0, and out-of-phase when J_{12}>0 and J_{21}<0.

1.3: Three-component system

For a three-component system of the form above, the dispersal relationship takes the form

$$\lambda^3 + a_1(q^2)\lambda^2 + a_2(q^2)\lambda + a_3(q^2) = 0,$$

with coefficients

$$a_1(q^2) = -(J_{11} + J_{22} + J_{33}) + q^2(D_1 + D_2 + D_3),$$

$$a_2(q^2) = (J_{11}J_{22} - J_{12}J_{21} + J_{11}J_{33} - J_{13}J_{31} + J_{22}J_{33} - J_{23}J_{32}) - q^2(J_1D_2 + J_1D_3 + J_2D_1 + J_2D_3 + J_3D_1 + J_3D_2) + q^4(D_1D_2 + D_1D_3 + D_2D_3),$$

$$a_3(q^2) = -(J_{11}J_{22}J_{33} + J_{12}J_{23}J_{31} + J_{13}J_{32}J_{21} - J_{11}J_{23}J_{32} - J_{22}J_{13}J_{31} - J_{33}J_{12}J_{21}) + q^2((J_{11}J_{22} - J_{12}J_{21})D_3 + (J_{11}J_{33} - J_{13}J_{31})D_2 + (J_{22}J_{33} - J_{23}J_{32})D_1) - q^4(J_{11}D_2D_3 + J_{22}D_1D_3 + J_{33}D_1D_2) + q^6 D_1D_2D_3.$$

Following the approach of White and Gilligan (1998), we considered the Routh-Hurwitz criteria. Stability without diffusion requires

$$a_1(0) > 0 \ \wedge \ a_3(0) > 0 \ \wedge \ a_1(0)a_2(0) - a_3(0) > 0$$

and instability with diffusion requires

$$a_1(q^2) < 0 \ \vee \ a_3(q^2) < 0 \ \vee \ a_1(q^2)a_2(q^2) - a_3(q^2) < 0.$$

Development • Supplementary information

As detailed by White and Gilligan (1998), the conditions for instability can only be satisfied if

$$a_3(q^2) < 0$$

or

$$a_1(q^2)a_2(q^2) - a_3(q^2) < 0.$$

Both of the functions in the inequalities are cubic functions of the form

$$y(q^2) = a(q^2)^3 + b(q^2)^2 + c(q^2) + d,$$

where a, b, c and d are constant coefficients. A DDI will occur if there exists a minimum turning point q^2_{TP}, for which $q^2_{TP} > 0$, and $y(q^2_{TP}) < 0$ (see White and Gilligan (1998) for details).

RD systems with more than two components can destabilise either as monotonically growing spatially periodic patterns (as for two component systems) or temporally oscillating patterns. When the system is destabilized with real positive λ, the spatial patterns grow monotonically, while if λ has non-zero imaginary parts, they will oscillate temporally. White and Gilligan (1998) provide a detailed analysis of conditions for monotonically growing and oscillating patterns. From their analysis, a parameterization will be produce monotonically growing spatial patterns for all q^2 if

$$a_3(q^2) < 0,$$

$$a_1(q^2)a_2(q^2) - a_3(q^2) > 0,$$

and

$$a_1(q^2)^2 - a_2(q^2) > 0.$$

The function in the final condition is quadratic in q^2, with a minimum at q^2_{MIN}, such that $a_1(q^2_{MIN})^2 - a_2(q^2_{MIN}) > 0$.

Development • Supplementary information

For each parameterization, a phase relationship was calculated as the eigenvector of the largest positive eigenvalue of the matrix

$$\boldsymbol{S} = \begin{pmatrix} J_{11} - D_1 q^2 & J_{12} & J_{13} \\ J_{21} & J_{22} - D_2 q^2 & J_{23} \\ J_{31} & J_{32} & J_{33} - D_3 q^2 \end{pmatrix},$$

calculated at the turning point q_{TP}^2 for which $a_3(q^2) < 0$. While this is not necessarily the phase pattern with which the pattern with the fastest growing wavelength will grow, it does give a phase pattern that is consistent with the network topology defined by the reaction matrix.

1.4: N-component system

For a general reaction diffusion system, Marcon et al. (2016) demonstrated that coefficients of the dispersion relationship, $a_k(q^2)$ for k =1,...,N, are of the form

$$a_k(q^2) = \sum_{\gamma_k \subseteq S_k^N} \left\{ (-1)^k \det[\boldsymbol{J}(\gamma_k)] + \sum_{m=1}^{k-1} q^{2(k-m)} \sum_{\gamma_m \subset \gamma_k} (-1)^m \det[\boldsymbol{J}(\gamma_m)] \det[\boldsymbol{D}(\tilde{\gamma}_m)] + q^{2k} \det[\boldsymbol{D}(\gamma_k)] \right\}, \quad (1.1)$$

where γ_k denotes a sequence of k distinct integers $\{i_1, \dots, i_k\}$, where $1 \leq i_1 < i_2 < \cdots < i_k \leq N$ and S_k^N is the set of all possible sequences of k elements in $\{1, \dots, N\}$, $\boldsymbol{J}(\gamma_k)$ denotes the $k \times k$ submatrix made up of coefficients with the column and row indices γ_k, and γ_m denotes a sequence of $m < k$ distinct integers, where $\gamma_m \subset \gamma_k$, and $\tilde{\gamma}_m$ is a complementary sequence of integers such that $\gamma_m \cap \tilde{\gamma}_m = 0$ and $\gamma_m \cup \tilde{\gamma}_m = \gamma_k$. As outlined in Marcon et al. (2016), there will be a single real and positive eigenvalue (and monotonically growing patterns will grow) if $a_k(q^2) > 0$ for $k < N$, and $a_N(q^2) < 0$.

Development • Supplementary information

2: Perturbation predictions from RD reaction terms

Our analysis of the response of RD systems to perturbations suggested that there is a relationship between the feedback loops within the RD system, and the nature of the response of the system to perturbation. Specifically, whether a component increases or decreases its level in response to the inhibition of another component depends on whether the components are in a positive or negative feedback loop. As the feedback loops are encoded by the reaction terms of the system, we considered what role the reaction terms could play in this behaviour. Preliminary simulations suggested that the response of an established spatially periodic steady state solution of an RD system to perturbation is predicted by the response of the reaction term equilibrium, and whether it increases or decreases in response to equivalent perturbations. We therefore decided to investigate further the factors that govern the responses of the reaction terms.

2.1: Shape of N-component response

We considered the response of a system of N components, using a system of linear ODEs equivalent to that used for the full reaction-diffusion system we used before, where

$$\frac{du_i}{dt} = \sum_{j=1}^{N} a_{ij}u_j + b_i - c_iu_i.$$

The parameters a_{ij} represent the weight of the interaction from component u_j to component u_i, where a positive term indicates an activation and a negative term an inhibition, b_i is a background production term and c_i is the linear degradation rate of component u_i.

The position of the equilibrium is given by setting $\frac{du_i}{dt} = 0$ for all i, and as in (Desoer 1960), rearranging this gives

$$u_i = (\boldsymbol{A}^{-1}\boldsymbol{b})_i = -\frac{\sum_{j=1}^{N} \mathrm{C}[\boldsymbol{A}]_{ji}b_j}{\det[\boldsymbol{A}]},$$

where the matrix $\boldsymbol{A}$ is given by

Development • Supplementary information

$$\boldsymbol{A} = \begin{pmatrix} a_{11} - c_1 & a_{12} & \cdots & a_{1N} \\ a_{21} & a_{22} - c_2 & \cdots & a_{2N} \\ \vdots & \vdots & \ddots & \vdots \\ a_{N1} & a_{N2} & \cdots & a_{NN} - c_N \end{pmatrix},$$

and $\mathrm{C}[\boldsymbol{A}]_{ji}$ is the cofactor of the element of the jth row and i^{th} column of $\boldsymbol{A}$.

We considered the effect of inhibiting a component on the equilibrium levels of the system. Specifically, we looked at the effect of inhibiting component u_1 on both the levels of u_1 itself, and the levels of other components by looking at the effect on a component u_2. As for our simulations of full RD systems we performed two forms of inhibition, namely inhibiting the response of a component to the levels of u_1 (analogous to inhibiting a "u_1 receptor") and inhibiting the production of u_1.

2.1.1: Inhibition of response to u_1

We first consider the effect of perturbing the response to u_1 by multiplying the weight of all interactions from component u_1 (to itself and all other components) by a factor of $1 - \alpha$ (i.e. with no inhibition when $\alpha = 0$ and complete inhibition when $\alpha = 1$). At equilibrium, this gave a system of the form

$$\frac{du_1}{dt} = 0 = (1 - \alpha)a_{11}u_1 + \sum_{j=2}^{N} a_{1j}u_j + b_1 - c_1u_1$$

and

$$\frac{du_i}{dt} = 0 = (1 - \alpha)a_{i1}u_i + \sum_{j=2}^{N} a_{ij}u_j + b_i - c_iu_i, \qquad i = 2, \dots, N.$$

It should be noted that for the response of u_1 to its own inhibition, only the weight of a_{11} is reduced, as c_1 represents a linear degradation term and would therefore be unaffected directly by an inhibitor blocking a receptor.

Rearranging these equations gave equilibrium levels of u_i upon inhibition of strength α, where

Development • Supplementary information

$$u_1 = -\frac{\sum_{j=1}^{N} \mathrm{C}[\boldsymbol{A}]_{j1} b_j}{\det[\boldsymbol{A}] - \alpha \det[\boldsymbol{C}]} \qquad (2.1)$$

and

$$u_i = -\frac{\sum_{j=1}^{N} \mathrm{C}[\boldsymbol{A}]_{ji} b_j - \alpha \sum_{j=1}^{N} \mathrm{C}[\boldsymbol{C}]_{ji} b_j}{\det[\boldsymbol{A}] - \alpha \det[\boldsymbol{C}]} \qquad (2.2)$$

for i=2,...,N. Matrix **C**, which does not include the degradation term c_1, is given by

$$\boldsymbol{C} = \begin{pmatrix} a_{11} & a_{12} & \cdots & a_{1N} \\ a_{21} & a_{22} - c_2 & \cdots & a_{2N} \\ \vdots & \vdots & \ddots & \vdots \\ a_{N1} & a_{N2} & \cdots & a_{NN} - c_N \end{pmatrix},$$

and $\mathrm{C}[\boldsymbol{C}]_{ji}$ is the cofactor of the element of the jth row and i^{th} column of $\boldsymbol{C}$.

Response of u_1*:*

The response of u_1 to its own inhibition is governed by Equation (2.1), which is a function of α with horizontal and vertical asymptotes at

$u_1 = 0$

and

$$\alpha = \frac{\det[\boldsymbol{A}]}{\det[\boldsymbol{C}]},$$

respectively. The intercept of the u_1-axis lies at

$$u_1 = -\frac{\sum_{j=1}^{N} \mathrm{C}[\boldsymbol{C}]_{j1} b_j}{\det[\boldsymbol{A}]},$$

Development • Supplementary information

which is the unperturbed level of u_1 (i.e. when $\alpha = 0$). As the unperturbed level of u_1 has a positive value, the function in Equation (2.1) can take two forms, depending on whether the vertical asymptote corresponds to a positive or negative value of α (see Figure SN1 a).

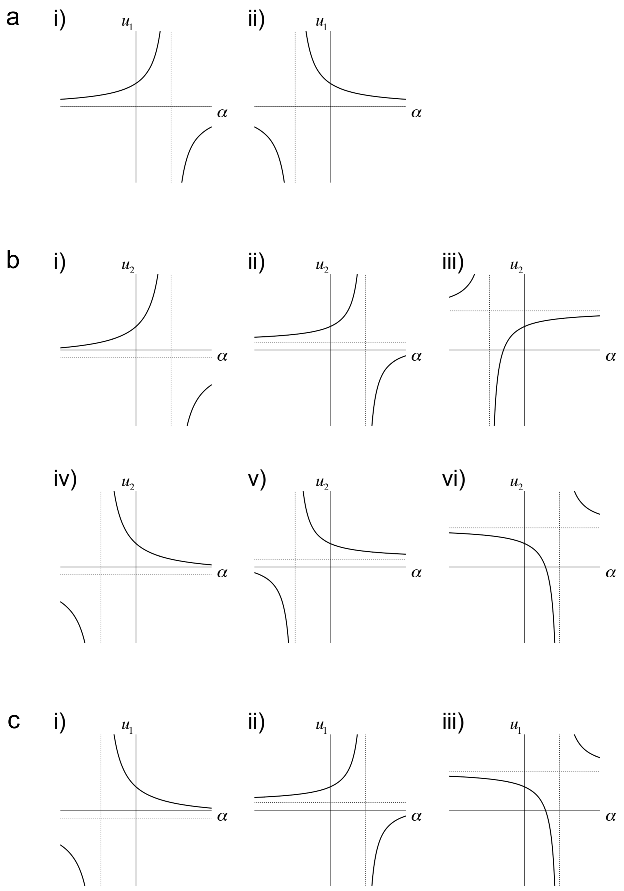

Figure SN1
a) Graphs showing the two possible responses of u_1 to the inhibition of u_1 by strength α. The vertical asymptote (dashed line) can lie at a positive (i) or negative (ii) value of α. The horizontal asymptote lies along the α-axis.
b) Graphs showing the six possible responses of u_2 to the inhibition of u_1 by strength α. The vertical asymptote (vertical dashed line) can lie at a positive (i, ii and vi) or negative (iii, iv and v) value of α. The horizontal asymptote (horizontal dashed line) can lie at a positive (ii, iii, v and vi) or negative (i and iv) value of u_2. The intercept of the α-axis can lie at a positive (ii, iv and vi) or negative (i, iii and v) value of α.
c) Graphs showing the three possible responses of $u_1$to the inhibition of u_1 by strength α. The vertical asymptote (vertical dashed line) can lie at a positive (ii and iii) or negative (i) value of α. The horizontal asymptote (horizontal dashed line) can lie at a positive (ii and iii) or negative (i) value of u_1. The intercept of the α-axis can lies at α=1.

Therefore, u_1 will increase with its own inhibition if

$$\frac{\det[\boldsymbol{A}]}{\det[\boldsymbol{C}]} > 0 \quad (2.3)$$

and will decrease if

$$\frac{\det[\boldsymbol{A}]}{\det[\boldsymbol{C}]} < 0. \quad (2.4)$$

Development • Supplementary information

Response of u_2:

The response of u_2 to inhibition of u_1 is governed by Equation (2.2), which is also a function of α with horizontal and vertical asymptotes, which lie at

$$u_2 = -\frac{\sum_{j=1}^{N} \mathrm{C}[\boldsymbol{C}]_{j2} b_j}{\det [\boldsymbol{C}]}$$

and

$$\alpha = \frac{\det [\boldsymbol{A}]}{\det [\boldsymbol{C}]},$$

respectively. The intercept of the u_2-axis lies at

$$u_2 = -\frac{\sum_{j=1}^{N} \mathrm{C}[\boldsymbol{A}]_{j2} b_j}{\det [\boldsymbol{A}]},$$

which is the unperturbed state (when $\alpha = 0$) and has a positive value, while the intercept of the α-axis is at

$$\alpha = \frac{\sum_{j=1}^{N} \mathrm{C}[\boldsymbol{A}]_{ji} b_j}{\sum_{j=1}^{N} \mathrm{C}[\boldsymbol{C}]_{ji} b_j}.$$

Depending on the relative values of the asymptotes and intercepts, this function can take six forms (Figure SN1 b), for three of which (Figure SN1 b i–iii), u_2 increases with inhibition of u_1. For the remaining three (Figure SN1 b iv–vi), u_2 decreases with the inhibition of u_1. From the positions of the horizontal asymptote relative to the unperturbed state at $\alpha = 0$, for conditions i, ii, iv and v

$$-\frac{\sum_{j=1}^{N} \mathrm{C}[\boldsymbol{A}]_{j2} b_j}{\det [\boldsymbol{A}]} > -\frac{\sum_{j=1}^{N} \mathrm{C}[\boldsymbol{C}]_{j2} b_j}{\det [\boldsymbol{C}]},$$

while for conditions iii and vi

$$-\frac{\sum_{j=1}^{N} \mathrm{C}[\boldsymbol{A}]_{j2} b_j}{\det[\boldsymbol{A}]} < -\frac{\sum_{j=1}^{N} \mathrm{C}[\boldsymbol{C}]_{j2} b_j}{\det\,[\boldsymbol{C}]}.$$

However, from the position of the vertical asymptote, for i, ii and vi

$$\frac{\det[\boldsymbol{A}]}{\det[\boldsymbol{C}]} > 0,$$

while for iii, iv and v

$$\frac{\det[\boldsymbol{A}]}{\det[\boldsymbol{C}]} < 0.$$

Therefore, in conditions i, ii and iii, where u_2 increases on the inhibition of u_1,

$$\det[\boldsymbol{C}] \sum_{j=1}^{N} \mathrm{C}[\boldsymbol{A}]_{j2} b_j < \det[\boldsymbol{A}] \sum_{j=1}^{N} \mathrm{C}[\boldsymbol{C}]_{j2} b_j, \quad (2.5)$$

while in conditions iv, v and vi, where u_2 decreases on the inhibition of u_1,

$$\det[\boldsymbol{C}] \sum_{j=1}^{N} \mathrm{C}[\boldsymbol{A}]_{j2} b_j > \det\,[\boldsymbol{A}] \sum_{j=1}^{N} \mathrm{C}[\boldsymbol{C}]_{j2} b_j. \quad (2.6)$$

<u>2.1.2: Inhibition of production of u_1</u>

We next considered the effect of inhibiting the production of u_1 by reducing the weights of all the coefficients affecting the production of u_1 (including the constant term, but excluding the degradation coefficient) by a factor of 1-α. At equilibrium, this gives a system of the form

$$\frac{du_1}{dt} = 0 = (1-\alpha)(\sum_{j=1}^{N} a_{1j} u_j + b_1) - c_1 u_1$$

and

Development • Supplementary information

$$\frac{du_i}{dt} = 0 = \sum_{j=1}^{N} a_{ij}u_j + b_i - c_i u_i$$

for i=2,...,N. Rearranging these equations gives the equilibrium levels of u_i upon inhibition of strength α, where

$$u_1 = -\frac{\sum_{j=1}^{N} \mathrm{C}[\boldsymbol{A}]_{j1} b_j (1-\alpha)}{\det[\boldsymbol{A}] - \alpha \det[\boldsymbol{C}]} \quad (2.9)$$

and

$$u_i = -\frac{\sum_{j=1}^{N} \mathrm{C}[\boldsymbol{A}]_{ji} b_j - \alpha \sum_{j=1}^{N} \mathrm{C}[\boldsymbol{C}]_{ji} b_j}{\det[\boldsymbol{A}] - \alpha \det[\boldsymbol{C}]}$$

for i=2,...,N. As the equation giving the equilibrium value of u_i where $i \neq 1$ is the same as that for the inhibition of the response (Equation (2.2)), the shape of the response of u_2 to an inhibition of strength α is the same for inhibiting either the production of, or the response to, u_1.

Behaviour of u_1*:*

The behaviour of the equilibrium value of u_1 is again governed by a function of α (Equation (2.9)) with horizontal and vertical asymptotes, which now lie at

$$u_1 = -\frac{\sum_{j=1}^{N} \mathrm{C}[\boldsymbol{A}]_{j1} b_j}{\det[\boldsymbol{C}]}$$

and

$$\alpha = \frac{\det[\boldsymbol{A}]}{\det[\boldsymbol{C}]},$$

respectively. The intercept of the u_1-axis lies at

Development • Supplementary information

$$u_1 = -\frac{\sum_{j=1}^{N} \mathrm{C}[\boldsymbol{A}]_{j1} b_j}{\det[\boldsymbol{A}]},$$

which is the unperturbed state and has a positive value, while the intercept of the α-axis is at $\alpha = 1$.

Depending on the relative positions of the asymptotes and intercepts, this function can take three forms (Figure SN1 c), for two of which (Figure SN1 c i, iii), the equilibrium value of u_1 decreases with its inhibition, while for the remaining one (Figure SN1 c ii), the equilibrium value of u_1 increases with its inhibition. Therefore, the equilibrium value of u_1 will increase on its inhibition if

$$\frac{\det[\boldsymbol{A}]}{\det[\boldsymbol{C}]} < 0 \quad (2.10)$$

or

$$\frac{\det[\boldsymbol{A}]}{\det[\boldsymbol{C}]} > 1. \quad (2.11)$$

While the equilibrium value of u_1 will decrease on its inhibition if

$$0 < \frac{\det[\boldsymbol{A}]}{\det[\boldsymbol{C}]} < 1. \quad (2.12)$$

2.2: Comparison with a numerically modelled RD system

Using this framework, we could compare the responses to inhibition for the full 2- and three-component RD systems with the behaviour of the reaction terms. We took the parameterisations of the reaction terms used in the full RD simulations (Figure 3 and Supplementary Figure S11) and inferred the direction of response by calculating the relative position of the asymptotes and intercepts. Comparison of the direction of the response to an inhibition for the reaction terms alone and for the full RD system (Figure SN2) showed that the pattern of the responses is very similar. This suggests that the response to an inhibition is predominantly dictated by the response of the reaction terms.

Development • Supplementary information

What about the small proportion of parameterisations for which the response of the equilibrium values differs between the full RD system and the analysis of the reaction terms alone? Interestingly, repeating the simulations, but reducing the strength of the inhibition by a factor of 0.25 or 0.25^2, successively increased the discrepancy (Figure SN2). As decreasing the strength of an inhibition decreases the magnitude of the response for the reaction terms, this suggests that the response of the reaction terms will dictate the response of the full RD system provided that the responses are sufficiently large. As the only difference between the reaction term systems and the full RD systems is diffusion, this suggests that if the response to an inhibition of the reaction terms is not sufficiently large, there can be a significant effect of diffusion, which in some cases can change the direction of response generated by the reaction terms alone.

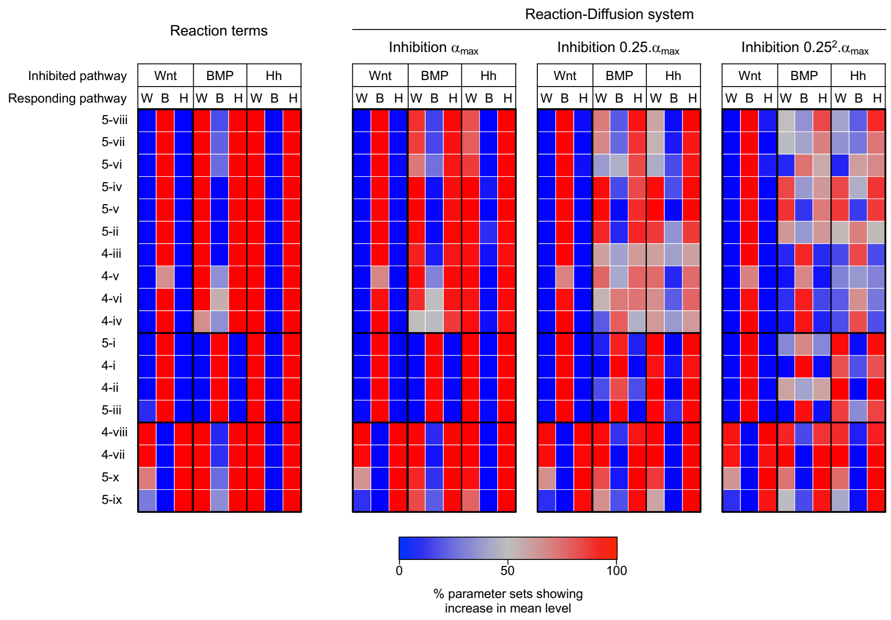

Figure SN2
Full maps showing the percentage of parameter sets where the level of each component increases in response to the inhibition of each component in the network in reaction term analysis and in RD simulations. The left hand heat map shows the response for reaction term analysis for the parameter sets used in three-component RD analysis, evaluated using criteria from Section 2. The second heat map, showing the Reaction-Diffusion system with inhibition strength α_{max}, is a reproduction of the heat map from Figure 3, and the heat maps with inhibition strength $0.25.\alpha_{max}$ and $0.25^2.\alpha_{max}$ are from reruns of the three-component RD simulations, replacing the inhibition parameter value α with values of $0.25.\alpha$ and $0.25^2.\alpha$ respectively. Pathway topology names are as in Figure 3.

This is likely to be generally true for RD systems based on the following argument. Large perturbations in reaction terms will produce large shifts in the mean value of the periodic pattern while changes in the diffusion terms will flatten or sharpen the peaks and troughs. For example, an increase in the reaction term will increase the value of u_i in both peaks and troughs.

Development • Supplementary information

For equilibrium to be restored, the diffusion term $D_i \frac{\partial^2 u_i}{\partial x^2}$ has to decrease. Since at the peaks, $D_i \frac{\partial^2 u_i}{\partial x^2}$ is negative, this means it has to become more negative, i.e. the peaks must become sharper. Conversely, the troughs must become flatter (the positive second derivative becomes less positive). These changes in the curvature of the peaks and troughs do not necessarily have a net effect on the average levels that are the same or opposite to the reaction term change. Our simulations, however, say that their effects do not increase at the same rate as those of the reaction terms. The simulations suggest that their role is more pronounced for small reaction term changes although we have not been able to demonstrate this analytically.

Development • Supplementary information

3: A graphical interpretation of the responses to perturbation

We wanted to understand how the position of a component in a network affects the response of the system to its inhibition. In particular, we wanted to understand how the response depends on the type of feedback loop the component is found in. In a recent study Marcon et al. (2016) used such a graphical approach to understand the conditions for DDI, in terms of stabilizing and destabilizing contributions of different feedback loops. This was based on an interpretation of the determinant as a combination of the cycles defined by the reaction matrix. As the conditions determining the response to perturbation (see Section 2) are in part dependent on the determinants of matrices $\boldsymbol{A}$ and $\boldsymbol{C}$, we took a similar approach.

In brief, a matrix $\boldsymbol{M}$ (such as the reaction matrices $\boldsymbol{A}$ and $\boldsymbol{C}$, or in the approach of Marcon et al. the Jacobian matrix $\boldsymbol{J}$) has associated graph Gr[$\boldsymbol{M}$] which is a labelled, weighted, directed graph of N nodes with edges from node j to node i of weight a_{ij}, where $a_{ij} \neq 0$ are the elements of the matrix $\boldsymbol{M}$. This is the graphical representation of the reaction network that we have been using throughout to depict the signs of the interactions as a network topology. Such a graph contains cycles, where a path can be traced from any node, through a series of other nodes, back to itself (or directly onto itself without passing through any other nodes). A cycle represents a positive feedback loop if the product of the weights of the edges is positive, and a negative feedback loop if the product is negative.

For any graph Gr[$\boldsymbol{M}$], a subgraph can be defined containing all the nodes but only a subset of the edges of Gr[$\boldsymbol{M}$], such that each node receives an input from only one other node and also outputs to only one other node (i.e. each node has both an in-degree and out-degree of 1). Such a subgraph is called a linear spanning subgraph (L-subgraph, l), and is made up of a set of 1 to N cycles through all nodes. The weight of an L-subgraph $w(l)$ is given by the product of the weights of these cycles, with the weight of each cycle itself being the product of its constituent edges. The determinant of the $N \times N$ matrix $\boldsymbol{M}$ is given by the sum of the weights of all the L-subgraphs l of the graph Gr[$\boldsymbol{M}$], with signs as below

$$\det[\boldsymbol{M}] = (-1)^N \sum_{l \subseteq Gr[\boldsymbol{M}]} (-1)^{\sigma(l)} w(l), \quad (3.1)$$

Development • Supplementary information

where $\sigma(l)$ is the number of cycles in the L-spanning subgraph l. Equation (3.1) is known as the Coates formula; for more details on the derivation, see Marcon et al. (2016) and references therein.

3.1: Response of u_1

As demonstrated in Section 2, the response of u_1 to its own inhibition depends on the sign and magnitude of the ratio of det[$\boldsymbol{C}$] to det[$\boldsymbol{A}$]. The sign is determined by the criteria for a DDI (see Section 1). To understand the response of u_1 to its own inhibition in graphical terms, we therefore considered these determinants in terms of the feedback loops (or cycles) through u_1 in matrix $\boldsymbol{C}$. From Equation (3.1), each summand in det [$\boldsymbol{C}$] can be written in the form $(-1)^N(-1)^{\sigma(l)}w(l)$, where l is a linear spanning subgraph of Gr[$\boldsymbol{C}$]. As all nodes in an l-graph have an in-degree and an out-degree of 1, for each summand in Equation (3.1) there is a single cycle containing node u_1, which comprises a set of n nodes, where $1 \leq n \leq N$, with κ_n a sequence of integers $\{i_1,i_2,\ldots,i_n\}$ which defines the sequence of nodes u_i through which the cycles passes, with each such sequence starting at i_1=1. If n=1, the cycle forms an interaction from u_1 directly onto itself, while if n=N, the cycle passes through all nodes. S_{κ_n} is the set of all possible κ_n, and $w(\kappa_n)$ is the weight of the cycle defined by κ_n (positive for a positive feedback loop, negative for a negative feedback loop). For each such cycle, where n<N, the nodes excluded from the cycle form an L-graph l_n consisting of 1 to N-n cycles between the remaining nodes, where $\sigma(l_n)$ is the number of remaining cycles (with $\sigma(l_n) = \sigma(l) - 1$ as one cycle has been removed). $w(l_n)$ is the product of the weights of the remaining cycles such that $w(l) = w(\kappa_n)w(l_n)$, and S_{l_n} is the set of all possible L-graphs l_n for a given κ_n.

Therefore, for any term in the determinant, the loop through u_1 can be factored out to give

$$(-1)^N(-1)^{\sigma(l)}w(l) = -(-1)^n w(\kappa_n)(-1)^{N-n}(-1)^{\sigma(l_n)}w(l_n).$$

Gathering together determinant terms containing the same cycle through u_1, the determinant can be rewritten

Development • Supplementary information

$$\det[\boldsymbol{C}] = -\sum_{\kappa_n \subseteq S_{\kappa_n}} (-1)^n w(\kappa_n)(-1)^{N-n} \sum_{l_n \subseteq S_{l_n}} (-1)^{\sigma(l_n)} w(l_n)$$

$$= -\sum_{\kappa_n \subseteq S_{\kappa_n}} (-1)^n w(\kappa_n) \det[\boldsymbol{C}(\tilde{\kappa}_n)], \quad (3.2)$$

where $\boldsymbol{C}(\tilde{\kappa}_n)$ is the submatrix of $\boldsymbol{C}$ excluding the columns and rows defined in κ_n (for n=N, $\det[\boldsymbol{C}(\tilde{\kappa}_n)] = 1$, as it is the determinant of a 0×0 matrix).

3.1.1: Inhibiting the response to u_1

As demonstrated in Section 2, the behaviour of u_1 on inhibiting the response to u_1 is dependent on whether the ratio $\frac{\det[\boldsymbol{A}]}{\det[\boldsymbol{C}]}$ is positive or negative (see Equations (2.3) and (2.4)). Therefore, u_1 will increase in response to its own inhibition if

$$\frac{\sum_{\kappa_n \subseteq S_{\kappa_n}} (-1)^n w(\kappa_n) \det[\boldsymbol{C}(\tilde{\kappa}_n)]}{\det[\boldsymbol{A}]} < 0 \quad (3.3)$$

and will decrease if

$$\frac{\sum_{\kappa_n \subseteq S_{\kappa_n}} (-1)^n w(\kappa_n) \det[\boldsymbol{C}(\tilde{\kappa}_n)]}{\det[\boldsymbol{A}]} > 0, \quad (3.4)$$

where each summand contains a different cycle through u_1, each multiplied by the determinant of the submatrix made of components excluded from the cycle. The response of u_1 to the inhibition of its response therefore depends on the natures of the feedback loops through u_1, and the stability of the components excluded from the loops.

3.1.2: Inhibiting the production of u_1

For inhibiting the production of u_1, we demonstrated in Section 2 that the response of u_1 again depends on $\det[\boldsymbol{A}]$ and $\det[\boldsymbol{C}]$ (see inequalities 2.10 – 2.12). First considering when N is even,

Development • Supplementary information

from Section 1 it follows that $\det[\boldsymbol{A}] > 0$. To satisfy the condition (2.10), $\det[\boldsymbol{C}] < 0$, and as a consequence, $\det[\boldsymbol{A}] > \det[\boldsymbol{C}]$. Otherwise, $\det[\boldsymbol{C}] > 0$, and in this case, to also satisfy condition (2.11), it follows that again $\det[\boldsymbol{A}] > \det[\boldsymbol{C}]$. However, when condition (2.12) holds, it follows that $\det[\boldsymbol{A}] < \det[\boldsymbol{C}]$. Therefore, when $\det[\boldsymbol{A}] < \det[\boldsymbol{C}]$, u_1 increases when its production is inhibited, while when $\det[\boldsymbol{A}] > \det[\boldsymbol{C}]$, u_1 decreases when its production is inhibited.

As matrices $\boldsymbol{A}$ and $\boldsymbol{C}$ only differ by the presence or absence of the $-c_1$ in the first matrix term, then

$$\det[\boldsymbol{A}] = \det[\boldsymbol{C}] - c_1 \det[\boldsymbol{C}(\tilde{\kappa}_1)],$$

where $\boldsymbol{C}(\tilde{\kappa}_1)$ is the submatrix of $\boldsymbol{C}$, excluding the first row and column. It therefore follows that u_1 will increase following its own inhibition if

$$c_1 \det[\boldsymbol{C}(\tilde{\kappa}_1)] > 0,$$

and u_1 will decrease following its own inhibition if

$$c_1 \det[\boldsymbol{C}(\tilde{\kappa}_1)] < 0.$$

Given that $c_1 > 0$, it follows that u_1 will increase when its production is inhibited if

$$\frac{\det[\boldsymbol{C}(\tilde{\kappa}_1)]}{\det[\boldsymbol{A}]} > 0, \quad (3.5)$$

and will decrease when

$$\frac{\det[\boldsymbol{C}(\tilde{\kappa}_1)]}{\det[\boldsymbol{A}]} < 0. \quad (3.6)$$

By the same reasoning, these conditions also hold if N is odd. The response of u_1 to the inhibition of its production therefore depends on the stability of the submatrix made from the remaining components.

Development • Supplementary information

3.2: Response of u_2:

As demonstrated in Section 2 (Equations (2.5) and (2.6)), the response of component u_2 in an N-component system to the inhibition of u_1 is in part determined by the relative signs and sizes of the determinants det[$\boldsymbol{A}$] and det[$\boldsymbol{C}$]. The response to inhibition also depends on the terms $\sum_{j=1}^{N} \mathrm{C}[\boldsymbol{A}]_{j2} b_j$ and $\sum_{j=1}^{N} \mathrm{C}[\boldsymbol{C}]_{j2} b_j$, which are of the same form as a determinant, but with the second column of matrix $\boldsymbol{A}$ and $\boldsymbol{C}$ respectively replaced by the vector of background production terms (b_1,b_2,…,b_N). Therefore, each term in these sums can similarly be interpreted graphically, as a path from an external node u_b to component u_2, with all components not involved in the path making up a set of cycles with each node having an in-degree and an out-degree of 1. Therefore, for each summand, every node has a single input and a single output, except u_b, which only has a single output and no input, and u_2, which only has a single input and no output.

Conditions (2.5) and (2.6) can be rearranged to give

$$c_1 \sum_{j=2}^{N} \mathrm{C}[\boldsymbol{C}(\tilde{\kappa}_1)]_{(j-1)1} a_{j1} \sum_{j=1}^{N} \mathrm{C}[\boldsymbol{A}]_{j1} b_j > 0 \qquad (3.7)$$

and

$$c_1 \sum_{j=2}^{N} \mathrm{C}[\boldsymbol{C}(\tilde{\kappa}_1)]_{(j-1)1} a_{j1} \sum_{j=1}^{N} \mathrm{C}[\boldsymbol{A}]_{j1} b_j < 0, \qquad (3.8)$$

respectively, where $\mathrm{C}[\boldsymbol{C}(\tilde{\kappa}_1)]_{(j-1)1}$ is the cofactor of the term a_{j2} from matrix $\boldsymbol{C}$ in the submatrix $\boldsymbol{C}(\tilde{\kappa}_1)$ (as this submatrix excludes the first row and column of $\boldsymbol{C}$, this will be the cofactor of the first column and the (j-1)th row).

The only term in conditions (3.7) and (3.8) whose sign is not constrained is $\sum_{j=2}^{N} \mathrm{C}[\boldsymbol{C}(\tilde{\kappa}_1)]_{(j-1)1} a_{j1}$. From Equations (2.1) and (2.9), which describe the levels of u_1 under inhibition, for the unperturbed state of u_1 to be positive, the term $\sum_{j=1}^{N} \mathrm{C}[\boldsymbol{A}]_{j1} b_j$ must have the same sign as the determinant det [$\boldsymbol{A}$] (positive if there are an even number of components and negative if there are an odd number). Also, $-c_1 < 0$ as it represents a degradation term. The form of the sum $\sum_{j=2}^{N} \mathrm{C}[\boldsymbol{C}(\tilde{\kappa}_1)]_{(j-1)1} a_{j1}$ is the same as that of $\sum_{j=1}^{N} \mathrm{C}[\boldsymbol{A}]_{j1} b_j$, and so it can be

Development • Supplementary information

interpreted as a sum where each term represents a path from component u_1 to component u_2. Specifically,

$$\sum_{j=2}^{N} \mathrm{C}[\boldsymbol{A}(\tilde{\kappa}_1)]_{(j-1)1} a_{j1} = -\sum_{p_n \subseteq S_{p_n}} (-1)^n w(p_n) \det[\boldsymbol{A}(\tilde{\kappa}_n)], \quad (3.9)$$

where p_n is a sequence of integers $\{i_1, i_2, \ldots, i_n\}$, where $2 \leq n \leq N$, starting with i_1=1 and ending with i_n=2, which defines the series of nodes u_i through which the path from u_1 to u_2 passes, S_{p_n} is the set of all possible such paths, and $w(p_n)$ the weight of the path p_n. The response of u_2 to the inhibition of u_1 therefore depends on the weight of the paths between the two nodes, and the stability of the components excluded from the paths.

4: Constraints on minimal topologies

Having established graphical conditions for the response of a system to inhibition of each component, we wanted to establish how the response of the system is related to network topology. As with our numerical investigation of three-component systems, we set out to understand the behavior of the subset of networks with the minimal requirements for patterning. We first establish the minimal requirements for a strongly-connected RD system, before considering how these networks respond to perturbation. We note that this subject has recently been dealt with extensively by Marcon et al and Diego et al, although as we assume all components degrade by default, we use a slightly different definition of minimal.

4.1: Identifying minimal topologies

4.1.1: Minimal conditions for a DDI

In order to establish the minimal requirements for DDI we asked what are the fewest interactions (excluding degradation terms) and feedback loops needed to satisfy the criteria for DDI. It should be noted that as we have been using a system of linear equations in our reaction-diffusion system, the reaction matrix $\boldsymbol{A}$ is the same as the Jacobian matrix $\boldsymbol{J}$, and the number of interactions is given by the number of non-zero terms a_{ij}. As described in Section 1, conditions for DDI that will always give rise to monotonically growing patterns require that $a_N(q^2) < 0$. Considering the terms that make up $a_N(q^2)$ (Equation (1.1)), the first term is made up solely of reaction terms and requires that $(-1)^N \det[\boldsymbol{J}]$ >0 in order to satisfy the criterion of stability in the absence of diffusion ($a_N(0) > 0$). The third term is made up solely of diffusion coefficients and the wavelength coefficient q^2, both of which are positive, meaning that $q^{2N} det[\boldsymbol{D}] > 0$. For the remaining terms, which contain both reaction and diffusion coefficients, as above for the diffusion and wavelength coefficients $q^{2(N-m)}\det[\boldsymbol{D}(\bar{\gamma}_m)] > 0$. Therefore, the conditions for DDI depend on the reaction terms and the signs of the various terms $(-1)^m \det[\boldsymbol{J}(\gamma_m)]$.

We first considered a system consisting of N components, without any interactions between them. These components will be only undergoing degradation (i.e. the only non-zero terms in $\boldsymbol{J}$ are on the diagonal, and are negative or in terms of reaction matrix $\boldsymbol{A}$, for all coefficients a_{ij}=0). From Equation (3.1), the only non-zero summands of any of the submatrix determinants

Development • Supplementary information

$\det[\boldsymbol{J}(\gamma_m)]$ in Equation (1.1) will be those consisting only of self-interactions (i.e. the degradation terms). It therefore follows that

$$(-1)^m \det[\boldsymbol{J}(\gamma_m)] = (-1)^m \prod_{i=1}^{m} J_{(\gamma_m)_i(\gamma_m)_i}, \quad (4.1)$$

which will always have a positive sign. Therefore, unsurprisingly, the condition for instability when there is diffusion cannot be satisfied in this simple system, since $a_N(q^2) < 0$.

In order to satisfy the conditions for DDI there must be at least one term in $a_N(q^2)$ with a negative sign. From Equation (4.1) this can be achieved by simply adding a single auto-activation term for a component. For example, if u_1 directly activates itself, providing that this auto-activation is strong enough to outweigh the degradation term for u_1 (in terms of matrix $\boldsymbol{A}$, $a_{11} - c_1 > 0$), a single term on the diagonal of the Jacobian matrix J_{11} will go from being negative to positive, resulting in the sign of any term $\det[\boldsymbol{J}(\gamma_m)]$ for which γ_m contains u_1 also changing sign. Alternatively, this can also be achieved by the addition of a single positive feedback loop through the set of r nodes defined by the sequence γ_r of weight $w(\gamma_r)$. For any $\det[\boldsymbol{J}(\gamma_m)]$ where γ_r is a subset of γ_m

$$\det[\boldsymbol{J}(\gamma_m)] = \left(\prod_{i=1}^{r} J_{(\gamma_r)_i(\gamma_r)_i} - (-1)^r w(\gamma_r)\right) \prod_{j=1}^{m-r} J_{(\tilde{\gamma}_r)_j(\tilde{\gamma}_r)_j},$$

where $\tilde{\gamma}_r$ is a complementary sequence of integers to γ_r such that $\gamma_r \cap \tilde{\gamma}_r = 0$ and $\gamma_r \cup \tilde{\gamma}_r = \gamma_m$. Again, provided that the weight of the positive feedback loop is greater than for the degradation terms, $\det[\boldsymbol{J}(\gamma_m)]$ will change sign.

However, as the sign of any submatrix containing the positive feedback loop will change sign, it follows that with the addition of a single destabilising positive feedback loop, $(-1)^N \det[\boldsymbol{J}] < 0$. This will no longer satisfy the condition for stability in the absence of diffusion $a_N(0) > 0$. An additional positive summand in $a_N(0)$ is therefore needed to stabilize the system. By the above reasoning, this can most simply be achieved by the addition of a single negative feedback between a set of components γ_s, where $\gamma_s \neq \gamma_r$. If this negative feedback loop takes the form of a path from a node γ_r, through the remaining nodes in the system and back into any node in γ_r, a single additional term will be added to any $\det[\boldsymbol{J}(\gamma_m)]$ where γ_s is a subset of γ_m (including

Development • Supplementary information

those for which γ_r is also a different subset). If the weight of this loop is sufficiently large, then it can overcome the effect of the positive feedback loop, and satisfy $a_N(0) > 0$.

Moreover, as there will still exist γ_m for which γ_r is a subset, but not γ_s, there will be terms $q^{2(k-m)}(-1)^m \det[\boldsymbol{J}(\gamma_m)] \det[\boldsymbol{D}(\tilde{\gamma}_m)]$ which will remain negative, even after the addition of a negative feedback loop to the system. Therefore, with the appropriate weighting of diffusion coefficients, there will exist conditions where $a_k(q^2) < 0$ when k=N, but not for other k, while maintaining $a_N(0) > 0$. As such $\tilde{\gamma}_m$ will by definition exclude components of the positive feedback loop through the nodes in γ_r, to achieve such a weighing, diffusion coefficients among components in the negative will be larger than those in the positive feedback loop. This gives an intuitive understanding for then general idea of local auto-activation and long-range inhibition in RD systems. In summary, a system containing single positive feedback loop coupled to a negative feedback loop is, therefore, sufficient to satisfy the conditions for a DDI for any size system, given appropriate strengths of interactions and diffusion coefficients.

Considering the minimal requirements for DDI in terms of the number of interactions between components in the interaction matrix $\boldsymbol{A}$ (including auto-activation terms a_{ii}, but not degradation terms c_i), in any such system a positive feedback loop through r components will add r interactions to the system, while the negative feedback loop though the remaining N-r components will add a further N-r+1 terms, resulting in a total of N+1 interactions. For any strongly-connected network, it is clear than fewer than N+1 interactions will not allow sufficient feedback loops to be made to satisfy the criteria for DDI, while other networks made of N+1 interactions (e.g. two positive or two negative feedback loops) cannot satisfy the conditions for DDI. While there are other ways of satisfying these conditions, they will require additional feedback loops, and therefore additional interaction. Therefore, in a system of N components, the combination of a single positive feedback loop through one to N-1 components, and a negative feedback loop through the remaining components is the minimal requirement for a DDI.

4.1.2: The phase of minimal DDI systems

Having established the architecture of minimal topologies, we next sought to establish the relative phase of the components for each of these topologies. This is dictated by the signs of

Development • Supplementary information

the components of the eigenvector corresponding to the largest positive eigenvalue of the Jacobian matrix: components with the same sign will be in the same phase. From the derivation of conditions for DDI in Section 1, we have the relationship

$$\begin{pmatrix} J_{11} - D_1 q^2 - \lambda & J_{12} & \cdots & J_{1N} \\ J_{21} & J_{22} - D_2 q^2 - \lambda & \cdots & J_{2N} \\ \vdots & \vdots & \ddots & \vdots \\ J_{N1} & J_{N2} & \cdots & J_{NN} - D_N q - \lambda \end{pmatrix} \begin{pmatrix} u_1 \\ u_2 \\ \vdots \\ u_N \end{pmatrix} = 0.$$

For all minimal architectures, there is only one component that receives more than one input (excluding negative self-interactions). For all remaining components, the rows in the matrix will consist of only two non-zero terms: the degradation term on the diagonal, J_{ii} which is therefore negative, and an additional input term from another component J_{ij}, whose sign depends on the nature of the input. Therefore, each of these rows will take the form

$$(J_{ii} - D_i q^2 - \lambda)u_i + J_{ij} u_j = 0.$$

By definition, for a DDI λ>0, meaning that $J_{ii} - D_i q^2 - \lambda < 0$. Consequently, u_i and u_j will have the same sign, and therefore be in-phase with each other if J_{ij} is positive, while they will have opposing signs and be out-of-phase for negative J_{ij}. Therefore, the relative phase of successive components in the positive and negative feedback loops can be inferred from the signs of interaction between them, with the exception of the interaction from a component in the negative feedback loop feeding into the positive feedback loop. Moreover, two components will be in-phase if the weight of the path between them is positive, and out-of-phase if negative, unless the path passes from the negative feedback loop into the positive feedback loop, in which case the relationship will be reversed. This is in keeping with recent finding on the relationship between topology and phase by Diego et al.

4.2: Responses to perturbation of minimal topologies

Having established minimal conditions for DDI, we next asked how these minimal systems respond to the inhibition of the different components. In particular, as these systems consist only of a single positive and a single negative feedback loop, we considered how the response to inhibition depends on the placement of a component relative to these two loops.

Development • Supplementary information

4.2.1: Inhibition of response

For the inhibition of response to u_1, in Section 3.1.1, conditions (3.3) and (3.4), it was shown that the response is dependent on the sign of

$$\frac{\sum_{\kappa_n \subseteq S_{\kappa_n}} (-1)^n w(\kappa_n) \det[\boldsymbol{C}(\tilde{\kappa}_n)]}{\det[\boldsymbol{A}]}, \quad (4.2)$$

where each summand corresponds to a different feedback loop through u_1. For minimal topologies, the sign of each summand depends on two features: whether the feedback loop supported by u_1 is a positive or negative feedback loop (given by $w(\kappa_n)$), and whether the nodes excluded from this loop support a positive feedback loop (given by $\det[\boldsymbol{C}(\tilde{\kappa}_n)]$). For each summand, $w(\kappa_n)$ will be negative if it represents a negative feedback loop and positive for a positive feedback loop. However, the relationship between the sign of $\det[\boldsymbol{C}(\tilde{\kappa}_n)]$, and the feedback loops contained depends on the number of nodes in the system described by submatrix $\boldsymbol{C}(\tilde{\kappa}_n)$ (see Section 1). Considering all possible combinations of odd and even numbers of components in matrix $\boldsymbol{A}$ and submatrix $\boldsymbol{C}(\tilde{\kappa}_n)$, it can be seen that $(-1)^n \det[\boldsymbol{C}(\tilde{\kappa}_n)] / \det[\boldsymbol{A}]$ will always be negative if submatrix $\boldsymbol{C}(\tilde{\kappa}_n)$ contains a single positive feedback loop, and will be positive if it only contains negative feedback loops.

As minimal systems only contain a single positive feedback loop and a single negative feedback loop, submatrix $\boldsymbol{C}(\tilde{\kappa}_n)$ will always contain only negative feedback loops as the two loops must share at least one component. Therefore, each summand in Equation (4.2) will be positive if it represents a positive feedback loop and negative if it represents a negative feedback loop. For certain placements in a minimal network, u_1 will only fall in one feedback loop, and there will be a single non-zero summand in Equation (4.2). Therefore, if u_1 is only found in the positive feedback loop it will decrease on its inhibition, and if it is found only in the negative feedback loop it will increase on its inhibition. However, for some placements in a minimal network u_1 will be in both positive and negative feedback loops. In these instances, there will be two summands in Equation (4.2), one positive and the other negative. The response of the system will depend on the relative magnitude of each term, with both an increase and a decrease in u_1 possible. These constraints are summarised in Figure 4b.

4.2.2: Inhibition of production

Development • Supplementary information

As shown in Section 3.1.2, conditions (3.5) and (3.6), the response of u_1 to the inhibition of its own production is determined by the sign of $\det[\boldsymbol{C}(\tilde{\kappa}_1)]$ relative to $\det[\boldsymbol{A}]$. This in turn, depends on whether the system described by the submatrix excluding u_1 contains the positive feedback loop of the minimal network or not. If u_1 is excluded from the positive feedback loop, the submatrix determinant $\det[\boldsymbol{C}(\tilde{\kappa}_1)]$ will therefore contain the single positive feedback loop in the system. As matrix $\boldsymbol{C}(\tilde{\kappa}_1)$ by definition has one fewer component than matrix $\boldsymbol{A}$, as detailed in equation 3.1, $\det[\boldsymbol{A}]$ and $\det[\boldsymbol{C}(\tilde{\kappa}_1)]$ will have the same sign. However, if u_1 is included from the positive feedback loop, regardless of whether it is also part of the negative feedback loop, $\det[\boldsymbol{C}(\tilde{\kappa}_1)]$ will only represents negative feedback loops, and $\det[\boldsymbol{A}]$ and $\det[\boldsymbol{C}(\tilde{\kappa}_1)]$ will have opposite signs. Therefore, if u_1 is excluded from the positive feedback loop, it will increase upon inhibition its production, while if it is part of the positive feedback loop (whether or not it is also in the negative feedback loop) it will decrease if its production is inhibited. These constraints are summarized in Figure 4b.

4.2.3: Response of u_2

Conditions for the response of component u_2 to inhibition of component u_1 were defined in supplemental note 3, conditions 3.7 and 3.8. Writing these in terms of paths between u_1 and u_2 (see equation 3.9) the response of u_2 depends on the sign of

$$-\sum_{p_n \subseteq S_{p_n}} (-1)^n w(p_n) \det[\boldsymbol{C}(\tilde{\kappa}_n)] \sum_{j=1}^{N} \mathrm{C}[\boldsymbol{A}]_{j1} b_j, \quad (4.3)$$

where each summand contains a different path from u_1 to u_2. Written in this form, it is apparent that the response of u_2 to the inhibition of u_1 is again dependent on two topological features of the minimal system: the weight of the path from component u_1 to u_2 (given by the term $w(p_n)$) and whether the nodes excluded from this path support a positive feedback loop (given by $\det[\boldsymbol{C}(\tilde{\kappa}_n)]$).

If there only exists a single path from u_1 to u_2, there will only be a single non-zero term in Equation (4.3). As shown in the above analysis of the phase relationship (Section 4.1.2), the sign of the path $w(p_k)$ will be positive if u_1 and u_2 are in-phase and negative if out-of-phase. The only exception is when u_1 is excluded from the positive feedback loop but the path to u_2 passes into

Development • Supplementary information

or through the positive feedback loop, in which case the relationship between the nodes is reversed. As detailed in Equation (3.1), the relationship between the sign of det $[\boldsymbol{C}(\tilde{\kappa}_n)]$ and the feedback loops contained depends on the number of nodes in the system described by submatrix $\boldsymbol{C}(\tilde{\kappa}_n)$ (see Section 1). Again, considering all possible combinations of odd and even numbers of components in submatrices $\boldsymbol{C}(\tilde{\kappa}_n)$ and path $w(p_k)$, and given that $\sum_{j=1}^{N} \mathrm{C}[\boldsymbol{A}]_{j1} b_j$ has the same sign as det $[\boldsymbol{A}]$ (see Section 2), the term $-(\sum_{j=1}^{N} \mathrm{C}[\boldsymbol{A}]_{j1} b_j)(-1)^n \det[\boldsymbol{C}(\tilde{\kappa}_n)]$ will be positive if det $[\boldsymbol{C}(\tilde{\kappa}_n)]$ contains the destabilising positive feedback loop, and negative otherwise.

Therefore, considering all possible placements of u_1 and u_2 relative to the two loops of the minimal system it can be seen that if u_1 is in the positive feedback loop (and therefore the positive feedback loop is not found in submatrix $\boldsymbol{C}(\tilde{\kappa}_n)$), u_2 will decrease if it is in-phase with u_1, and increase if out-of-phase. However, if u_1 is excluded from the positive feedback loop, u_2 will increase if it is in-phase with u_1, and decrease if out-of-phase, as either the path to u_2 will pass from the negative feedback loop into the positive feedback loop, or this path will exclude components from the positive feedback loop. These constraints are summarized in Figure 4b.

It should be noted that in certain instances when u_1 and u_2 lie in both the positive and negative feedback loop, two paths can be traced from u_1 to u_2: one consisting of interactions only found in the positive feedback loop, and the other comprising ones only found in the negative feedback loop. There will therefore be two summands in Equation (4.3), one associated with each path, and they will be of opposing signs. Therefore, the response of u_2 to the inhibition of u_1 will depend on the relative weight of each path, and it can either increase or decrease.

It should be noted that while the response of u_2 to the inhibition of u_1 is not constrained by topology in these instances, it is coupled with the responses of other components in the system. Factoring out all coefficients shared between the two summands leaves

$$(-1)^{w-v(w^{+})} w(p_{w^{+}}) \det[\boldsymbol{W}(\tilde{\kappa}_{w^{+}})] + (-1)^{w-v(w^{-})} w(p_{w^{-}}) \det[\boldsymbol{W}(\tilde{\kappa}_{w^{-}})], \quad (4.4)$$

where matrix $\boldsymbol{W}$ is the submatrix $\boldsymbol{A}(\gamma_w)$ comprising the w components supporting the two paths defined by γ_w. The terms $w(p_{w^{+}})$ and $w(p_{w^{-}})$ give the weights of the paths through the positive and negative loops, respectively, $v(w^{+})$ and $v(w^{-})$ the numbers of components in the respective paths, and $\boldsymbol{W}(\tilde{\kappa}_{w^{+}})$ and $\boldsymbol{W}(\tilde{\kappa}_{w^{+}})$ the submatrices of $\boldsymbol{W}$ comprising the components excluded from the respective paths. This same sum is also left when considering the response

Development • Supplementary information

of u_1 to its own inhibition if it falls in both feedback loops, and shared terms are factored out of Equation (4.2). In both instances, if the weight through the positive feedback loop is greater than that through the negative feedback loop, that is

$$\left|(-1)^{w-\nu(w^+)}w(p_{w^+})\det[\boldsymbol{W}(\tilde{\kappa}_{w^+})]\right| > \left|(-1)^{w-\nu(w^-)}w(p_{w^-})\det[\boldsymbol{W}(\tilde{\kappa}_{w^-})]\right|,$$

then u_1 will decrease on its own inhibition, as will any in-phase components, with out-of-phase components increasing. However, when the inequality is reversed and u_1 increases on its own inhibition, the response of any component to which the two paths can be traced will be coupled, increasing if it is in-phase, and decreasing if out-of-phase.

4.3: Adding nodes to minimal topologies

In the above analysis of the criteria for a minimal topology (Section 4.1.1) we only considered strongly-connected networks, where the two feedback loops comprise all N components. For systems where the two loops only pass through a subset of components, if the remaining components respond passively (i.e. do not feed back into the system) each component will only add a single interaction. Like the strongly-connected networks these networks also have two loops and N+1 interactions.

These passive components can be wired back into the minimal RD 'core' by adding interactions from the passive components back to the core. If the weights of any additional interactions, or the weights of additional loops generated by them, are not excessively large, the criteria for DDI will still be met, and the phase relationship of the related not strongly-connected minimal topology will also be maintained. While the resulting topologies are not strictly minimal in terms of the number of loops or interactions, they comprise additional strongly-connected networks which satisfy the conditions for DDI. Moreover, as the removal of an interaction will either result in the system either no longer satisfying the criteria for DDI or no longer being strongly-connected, we will also consider such topologies as an additional form of minimal topology.

We will now consider the effect of inhibition on networks with additional components wired in outside of the RD 'core'. We first will consider the case where there is a single component u_1, external to the core, and the effect of inhibiting this component on its own levels, before

Development • Supplementary information

considering the effect of inhibiting u_1 on core components. We then consider the effect of external interactions on the core.

4.3.1: Effect on self

The addition of a single component external to the RD core will generally result in the addition of a single positive feedback loop to the system (we will discuss below the subset of cases where the addition of a single component results in the addition of two loops).

Inhibition of response

As for the minimal topologies (Section 4.2.1) when the response to u_1 is inhibited, the behaviour of u_1 depends on the sign of the sum in Equation (4.2). This sum will consist of a single term, and as before, the sign of this term depends in part on whether or not the submatrix $\boldsymbol{C}(\tilde{\kappa}_n)$ contains the destabilising core positive feedback loop, and also whether this loop supports positive or negative feedback. We will ask how the wiring of this loop relative to the core positive feedback loop, along with the weighting of the loop, determines the response to inhibition.

We will first consider when the feedback loop through u_1 includes components from the core positive feedback loop. In this case submatrix $\boldsymbol{C}(\tilde{\kappa}_n)$ contains only negative feedback loops and $(-1)^n \det[\boldsymbol{C}(\tilde{\kappa}_n)] / \det[\boldsymbol{A}]$ will be positive. If u_1 itself supports a positive feedback loop, condition (3.4) will be satisfied and u_1 will decrease on its own inhibition. Conversely, if u_1 supports a negative feedback loop, condition (3.3) will be satisfied and u_1 will increase on its own inhibition. These relationships are reversed if the loop through u_1 does not pass through any components in the core positive feedback loop. If this is the case, submatrix $\boldsymbol{C}(\tilde{\kappa}_n)$ will contain the core positive feedback loops and $(-1)^n \det[\boldsymbol{C}(\tilde{\kappa}_n)] / \det[\boldsymbol{A}]$ will be negative (otherwise conditions for DDI cannot be met — see Section 1). In this instance, if u_1 supports a positive feedback loop, condition (3.3) will now be satisfied and u_1 will increase on its own inhibition, while if u_1 supports a negative feedback loop, condition (3.4) will be satisfied and u_1 will decrease on its own inhibition.

Interestingly, the response of u_1 to its inhibition does not correlate directly with the feedback loop u_1 supports, but rather with the net feedback provided to the core positive feedback loop

Development • Supplementary information

(i.e. the weight the path from u_1 to a core positive feedback component multiplied by the weight of the return path). If u_1 supports a net positive feedback, it will decrease on its own inhibition, while if it provides a net negative feedback, it will increase on its own inhibition. These constraints are summarized in Figure 4d.

Inhibition of production

As demonstrated in Section 3, the response to the inhibition of production is dependent on the sign of det $[\boldsymbol{C}(\tilde{\kappa}_1)]$ relative to det $[\boldsymbol{A}]$ (condition (3.5) or (3.6)). As det$[\boldsymbol{A}]$ and det$[\boldsymbol{C}]$ only differ by the presence of the degradation coefficient c_1 in the first matrix term it follows that

$$1 = \frac{-(-1)^n w(\kappa_n)\,\det[\boldsymbol{C}(\tilde{\kappa}_n)] - c_1 \det[\boldsymbol{C}(\tilde{\kappa}_1)]}{\det\,[\boldsymbol{A}]}.$$

In the above analysis of the inhibition of response, we demonstrated how network topology (i.e. the sign of the loop containing u_1, and how this feeds into the core RD network) determines the sign of $(-1)^n w(\kappa_n)$det $[\boldsymbol{C}(\tilde{\kappa}_n)]$ /det $[\boldsymbol{A}]$. From these arguments, it follows that if u_1 supports a net positive feedback, $-c_1$ det$[\boldsymbol{C}(\tilde{\kappa}_1)]$/ det $[\boldsymbol{A}]$ must be negative. As $-c_1$ is negative, condition (3.6) will be satisfied and u_1 will decrease. However, if u_1 supports a net negative feedback, the sign of $-c_1$ det$[\boldsymbol{C}(\tilde{\kappa}_1)]$/ det $[\boldsymbol{A}]$ is not constrained and therefore both conditions (3.5) and (3.6) can be satisfied. Therefore, u_1 will decrease on the inhibition of its production if it provides a net positive feedback to the core positive feedback loop, but depending on the strength of the different feedback loops, u_1 will either increase or decrease when its production is inhibited if it provides a net positive feedback. These constraints are summarized in Figure 4d.

It should be noted that in some instances where u_1 feeds into and out of the core RD network at components which are found in both core positive and negative feedback loops, it is possible that a second loop supported by u_1, through components only found in the core negative feedback loop, will also exist. In this case the two terms describing alternate paths through the positive feedback loop and negative loop (see Section 4.4) will again remain after shared components are factored out. As discussed above, in the cases where the magnitude of the path through the negative feedback loop is greater than the path through the positive feedback loop, the relationships between the response of u_1 to its own inhibition and the feedbacks it supports described here will also be inverted.

Development • Supplementary information

4.3.2: Effect on core components

We will now consider the how inhibiting the external component u_1 affects a core component u_2. We will consider the effect of whether u_1 feeds directly into the positive or negative feedback loop of the core RD network, and on whether u_2 itself lies in the core positive or negative feedback loop. As for minimal systems (Section 4.2.2), u_2 will decrease on the inhibition of u_1 if the sign of the path from u_1 to u_2 is positive, unless the submatrix determinant det $[\boldsymbol{C}(\tilde{\kappa}_n)]$ contains the positive feedback loop of the core RD network. Likewise, u_2 will increase if the path from u_1 to u_2 is negative, unless the path excludes the core RD network positive feedback loop.

If u_1 feeds into the core RD network through the positive feedback loop, det $[\boldsymbol{C}(\tilde{\kappa}_n)]$ will not contain the core positive feedback loop. The response will therefore depend on the sign of $w(p_k)$, such that if u_1 itself supports a positive feedback loop, the weight of the path from u_1 to u_2 will be positive if u_1 and u_2 are in-phase, and negative if they are out-of-phase. Therefore, u_2 will decrease if it is in-phase with u_1 and increase if it is out of phase. The opposite will occur if u_1 is in a negative feedback loop, with the level of u_2 increasing if it is in-phase with u_1 and decreasing if it is out-of-phase.

We next consider the case when u_1 feeds into the core through the negative feedback loop. If u_2 is also found in this loop, and the path from u_1 does not pass through the core positive feedback, the sign of the path will be positive if the components are in-phase and negative if they are out-of-phase, provided u_1 supports a positive feedback loop (and therefore provides a net negative feedback to the core positive feedback loop). The reverse is true if u_1 supports a negative feedback loop (and thus a net positive feedback). As the core positive feedback components are found in det $[\boldsymbol{C}(\tilde{\kappa}_n)]$, if u_2 is in-phase with u_1 it will decrease on inhibition if u_1 provides a net positive feedback, and out-of-phase components will increase, with the reverse true if u_1 provides a net negative feedback. The same is also true when u_1 feeds into the core negative feedback loop, but the path to u_2 passes into or through the core positive feedback loop. When the core positive feedback components are found no longer in submatrix $\boldsymbol{C}(\tilde{\kappa}_n)$ and therefore the sign of det $[\boldsymbol{C}(\tilde{\kappa}_n)]$ is reversed, the relationship between the signs of the path from u_1 to u_2 and the phase relationship are now inverted by passing back into the core positive feedback loop, maintaining the above relationship.

Development • Supplementary information

Therefore, regardless of where the path from u_1 feeds into the core RD network, if u_1 provides a net negative feedback loop, u_2 will increase if it is in-phase with u_1, and decrease if it is out-of-phase. The opposite will be true if u_2 supports a net positive feedback. These constraints are summarized in Figure 4d. Again, there can be instances where two paths of opposing sign can be traced from u_1 to u_2 if u_1 feeds into a component found in both core loops and u_2 is also found in both loops. The response is again dependent on the sign of Equation (4.4), with the above responses inverted if the magnitude of the term relating to the path through the negative feedback loop is greater than those relating to the positive feedback loop, with all components receiving input from the two paths responding in a coupled manner.

4.3.3: Effect of adding external components to an RD system

Finally, we considered the effect adding external components to existing constraints on the system. We asked whether the addition of an external component could change the behaviours within the minimal RD core. As shown above, a major feature determining the response of a component in a minimal RD network either to its own inhibition, or the inhibition of another component, is whether or not various submatrices contain the destabilizing positive feedback loop of the core RD network. In some instances, the addition of loops can change the stability of these submatrices, either making stable submatrices unstable by adding a positive feedback loop, or making unstable submatrices stable by the addition of further negative feedback. However, it should be noted that as the various loops and paths that determine the behaviours within the core will still be present, only certain placements of feedback loops have such effects, and that they only affect certain components under particular parameterisations. There will still exists parameterisations where the responses described above (summarized in Figure 4b) are maintained.

The constraints applying to the addition of one external component will, therefore, apply to any number of external components, provided that the magnitudes of additional interactions are not sufficiently large to change the stability of any submatrices. This also opens up the possibility of considering potential interactions between external components u_1 and u_2 (for simplicity we will only consider the cases where the path between u_1 and u_2 passes through the core). The path will contain the path from u_1 to a core component, plus an additional interaction to u_2. Therefore, the response of u_2 to the inhibition of u_1 will be the same as that of the preceding core component in the path if the final interaction is positive, and the opposite if it is

Development • Supplementary information

negative. The same is true of the response of external u_2 to a core u_1. Consequently, if u_1 is involved in positive feedback (either as the core loop or as a source of additional positive feedback), then u_2 will decrease if it is in-phase with u_1, and increase if it is out-of-phase, while if u_1 provides negative feedback the reverse is true. As has been discussed above, when two paths can be traced from u_2 to u_1, if the path through the negative feedback loop has a greater weighting these relationships will be inverted coupled to other such components.

Taken together, the effects of inhibiting u_1 on itself, and on u_2 primarily depend on whether u_1 falls in the core positive feedback loop, or provides an additional net positive feedback, or whether it provides negative feedback. While for some parameterisations of some topologies, certain components will behave differently, these constraints on the behavior of minimal systems provide a framework to consider the behavior of RD networks in response to inhibition experiments.

References

Desoer, C. A. (1960). "The Optimum Formula for the Gain of a Flow Graph or a Simple Derivation of Coates' Formula." Proceedings of the IRE **48**(5): 883-889.

Marcon, L., X. Diego, J. Sharpe and P. Muller (2016). "High-throughput mathematical analysis identifies Turing networks for patterning with equally diffusing signals." Elife **5**.

Murray, J. D. (2003). Mathematical Biology, Berlin: Springer-Verlag.

White, K. A. J. and C. A. Gilligan (1998). "Spatial heterogeneity in three species, plant–parasite–hyperparasite, systems." Philosophical Transactions of the Royal Society of London. Series B: Biological Sciences **353**(1368): 543-557.
