# Supplementary material for: Perturbation analysis of a multi-morphogen Turing reaction-diffusion stripe patterning system reveals key regulatory interactions
Source: Development. 2020 Oct 29;147(20):dev190553. doi: 10.1242/dev.190553 (PMC7648603; doi:10.1242/dev.190553)
Supplement: Reviewer comments [file develop-147-190553_review_history.pdf]

Perturbation analysis of a multi-morphogen Turing reaction-diffusion stripe patterning system reveals key regulatory interactions

Andrew D. Economou, Nicholas A.M. Monk and Jeremy B.A. Green
DOI: 10.1242/dev.190553

Editor: Paul Francois

Review timeline

Original submission:	12 March 2020
Editorial decision:	14 April 2020
First revision received:	14 July 2020
Editorial decision:	17 August 2020
Second revision received:	7 September 2020
Accepted:	11 September 2020

Original submission

First decision letter

MS ID#: DEVELOP/2020/190553

MS TITLE: Perturbation analysis of a multi-morphogen Turing Reaction-Diffusion stripe patterning system reveals key regulatory interactions

AUTHORS: Andrew D. Economou, Nicholas A.M. Monk, and Jeremy B.A. Green

I have now received all the referees' reports on the above manuscript, and have reached a decision. The referees' comments are appended below, or you can access them online: please go to BenchPress and click on the 'Manuscripts with Decisions' queue in the Author Area.

As you will see, the referees express considerable interest in your work, but have some significant criticisms and recommend a substantial revision of your manuscript before we can consider publication. If you are able to revise the manuscript along the lines suggested, which may involve further experiments, I will be happy receive a revised version of the manuscript. Your revised paper will be re-reviewed by one or more of the original referees, and acceptance of your manuscript will depend on your addressing satisfactorily the reviewers' major concerns. Please also note that Development will normally permit only one round of major revision.

We are aware that you may currently be unable to access the lab to undertake experimental revisions. If it would be helpful, we encourage you to contact us to discuss your revision in greater detail. Please send us a point-by-point response indicating where you are able to address concerns raised (either experimentally or by changes to the text) and where you will not be able to do so within the normal timeframe of a revision. We will then provide further guidance. Please also note that we are happy to extend revision timeframes as necessary.

Please attend to all of the reviewers' comments and ensure that you clearly highlight all changes made in the revised manuscript. Please avoid using 'Tracked changes' in Word files as these are lost in PDF conversion. I should be grateful if you would also provide a point-by-point response detailing how you have dealt with the points raised by the reviewers in the 'Response to Reviewers' box. If you do not agree with any of their criticisms or suggestions please explain clearly why this is so.

© 2020. Published by The Company of Biologists under the terms of the Creative Commons Attribution License (https://creativecommons.org/licenses/by/4.0/).

Reviewer 1

Advance summary and potential significance to field

SUMMARY

In this study, Economou et al. aim to understand the mechanism driving periodic patterning of rugae in the mammalian palate. Their overall strategy is to compare experimental data with results from simulations, and instead of suggesting just a single model, they show how they can constrain the space of possible models that are consistent with their data. I very much admire this approach.

However, I do have some concerns with how the comparison between data and models is performed, which I outline below.

Comments for the author

MAJOR COMMENTS

1. PHASE DIFFERENCES AMONGST PATHWAY COMPONENTS

The authors measure transcriptional readouts of relevant signalling pathways to reveal their phase-relationship to the Hh+ rugae. They then use this to constrain their model, which focuses on signalling pathways in aggregate rather than specifically modelling ligands / pathway activation separately.

Looking for phase relations between pathways is a good strategy and has been exploited with success in other systems (e.g. the limb, Raspopovic et al. 2014). However, in these studies, both experimental and modelling results revealed that multiple elements of the signalling pathway can be expressed out of phase with each other e.g. BMP2 and activated BMP signalling (pSmad) are out of phase with each other as digits are patterned. Therefore it is not obvious a priori that modelling each signalling pathway by a single variable is sufficient. Can the authors comment on this?

Two minor points:
a) is there data from the literature describing ligand / receptor expression in the rugae – this would be useful to summarize for the reader.

b) I do not necessarily agree with statements such as: “It may be argued that conceptually and pharmacologically, the most intelligible and accessible level of description is the signaling pathway”. What is the justification?

2. INTERPRETATION OF PATHWAY INHIBITION - EXPERIMENTAL DATA
The authors perform interesting perturbation experiments implicating BMP/HH/WNT/FGF pathways in rugae formation and maintenance. However I have the following concerns with these experiments:

a) To understand pattern formation (which is the focus of this study), it seems necessary to focus on yet-to-be-formed Hh+ stripes, since any Hh+ stripes that have already formed may be maintained by a different mechanism. If I understand correctly, the E13.5 explants will already have r1-5 & r8, and so shouldn’t the analysis only focus on r6/r7 whose formation occurs in the presence of drug? (This numbering is not my areas of expertise but it would be good to clarify this.) Narrowing in on these newly-forming rugae, it seems to me that the interpretation of the data may be somewhat different e.g. is cyclopamine significantly disrupting pattern formation? (similar to results in Economou et al. 2012)

b) The authors focus their interpretation on the levels of Hh following perturbation, and use this to constrain their mathematical models. However, it is unclear how the value of “mean Hh” is sufficient to capture the response of the system to the drug treatments. One could imagine that the spacing of the Hh+ stripes could change; there could be warping and misorientation of the Hh+ stripes; or there could be a complete failure to undergo pattern formation. Can the authors provide

© 2020. Published by The Company of Biologists under the terms of the Creative Commons Attribution License (https://creativecommons.org/licenses/by/4.0/).

more quantification of their expression data to show that changes in Hh patterning following drug treatment can be entirely captured by “mean Hh”, specifically for newly forming rugae as outlined above.

3. INTERPRETATION OF PATHWAY INHIBITION – MODELLING RESULTS

Related to the above point, the authors focus their attention on the “mean Hh” level following in silico perturbation. Can they provide evidence that their in silico perturbations are only affecting “mean Hh” level and not affecting other pattern parameters (e.g. wavelength, ability to break symmetry) - in other words, provide more evidence that the schematic in Figure 2b is representative of their modelling results. Here, it would be helpful not only to show when “mean Hh” does accurately capture their simulation results, but also provide clear examples / intuition of where this assumption fails.

E.g. I am somewhat surprised that their perturbations do not significantly affect pattern wavelength, since similar types of model (e.g. limb, Sheth et al. 2012, Raspopovic et al. 2014) show that changing reaction parameters naturally alters pattern wavelengths.

4. MODELLING TIMING OF GENE EXPRESSION DATA
In Figure 6, elegant experimental data suggests that some pathway elements develop periodic patterns later than others. This data is interesting and helps to inform what pathways are required for the initial periodic patterning.

However, a simple-minded interpretation of the data would be that BMP and mFGF are not required for the (initial) periodic patterning of rugae i.e. are not required for the DDI.
Perhaps they are required for later refinement/maintenance of the pattern. Is this interpretation correct?

If so, why do the authors consider BMP and mFGF in their models for DDI? (e.g. in Fig 3 BMP is included). Equally, can the authors provide evidence that their models in Figure 7 predict the observation “BMP and m-FGF responses trail Hh” and are not involved in DDI?

A minor comment: the kymograph in Figure 6 provides a nice view of the dynamics of the patterning process. What are the horizontal “bands” in the figure? Do they complicate the interpretation (I’m particularly looking at Gli1).

<u>Reviewer 2</u>

Advance summary and potential significance to field

In this excellent paper, Economou et al. provide a new framework to clarify how multiple signaling pathways together organize the formation of periodic rugae during mouse oral palate development. The authors first analyzed how rugae patterning was affected when four major signaling pathways were inhibited, and then carefully quantified the spatiotemporal responses to these signaling pathways. Perturbation of signaling using small molecule inhibitors implied Shh and BMP as inhibitors and FGF and Wnt as activators of rugae patterning. Shh and Wnt target genes spatially overlapped, whereas the expression of FGF and BMP targets was interdigitated with respect to Shh/Wnt responses. Furthermore, the authors found temporal differences in target gene expression patterns, and the response to these signaling pathways also varied in neighboring tissues suggesting the presence of mesenchymal and epithelial-specific FGF signaling pathways. The authors then set out to clarify how the four signaling pathways must be wired together to explain the experimental findings. Using different degrees of abstraction based on self-organizing reaction-diffusion systems, they identified an early interaction between Wnt, Shh and epithelial FGF with a later incorporation of BMP and mesenchymal FGF as the most parsimonious model. The paper is very well written and combines a good mix of new experimental findings Â– including beautiful and highly informative time course data Â– and in depth-mathematical analyses to suggest a new network of signaling interactions for rugae patterning.

© 2020. Published by The Company of Biologists under the terms of the Creative Commons Attribution License (https://creativecommons.org/licenses/by/4.0/).

Comments for the author

MAJOR POINTS
1) Data analysis and controls a) Please quantify the data in Fig. 1a. Are only the levels changed, or is the width of the peaks also different with inhibitor treatments? Are potential changes in stripe width consistent with the models?
b) Please show the efficacy of the drugs by staining for the respective target genes (as has already been done for SU-5402 and cyclopamine in Economou et al. 2012).
c) The manuscript and Supplementary Notes state “data not shown” in multiple instances. The authors should either show the data or remove the claims associated with these statements.

2) Perturbations and predictions
a) Please determine how each pathway (with the readout target genes shown in Fig. 1b) responds to inhibitor treatment. Is this consistent with the predictions of the models (e.g. Fig. S7)? In particular, according to Economou et al. 2012, cyclopamine treatment should broaden spry2 expression (i.e. Shh should normally inhibit FGF). This interaction is now very indirect (see Fig. 7c), and the authors should determine whether the corresponding expression changes in epithelial and mesenchymal FGF signaling targets are observed upon cyclopamine treatment.
b) The authors provide a strong new prediction by stating that the core 3-node network is later modified by BMP and mFGF: Transient application of a BMP inhibitor should have different effects on the patterning outcome at different developmental stages. Testing this prediction, if technically possible, would provide very strong support for the conclusions of the paper.

MINOR POINTS

1) Data analysis and presentation
a) Please describe how the normalization in Fig. 1b was performed.
b) Please clarify why networks that are not strongly connected are shown in Fig. 3a. On p. 11, it is stated that they had been filtered out.
c) Please show green/red data such as the one in Fig. 6a using a different color map that is suitable for a wider audience, and ideally show the channels separately.
d) I could not follow the argument “The two topologies cannot be combined into a three-component system […]”. For example, the network shown in the top middle of Fig. S5a is identical to the network iii shown in Fig. S8a. Please clarify.
e) Please correct “um” to “uM” in Fig. S2
f) In Fig. S8b, the positive feedback loops indicated in magenta are not clear (e.g. the one outlined for network iv has an overall negative weight). Please clarify.
g) Please provide the initial conditions and clarify how the system shown in Fig. 5a with purely inhibitory interactions can lead to stable spatiotemporal patterns. Intuitively, it seems that the concentration of all components should constantly decrease. Furthermore, “a_ii” in the Fig. 5 legend should be corrected to “a_ij”.
h) In the Methods section, please mention how the position of the arrowheads in Fig. 6a was determined. If it was done manually, please add an unbiased alternative method.

2) Manuscript organization
a) The authors state that “[…] FGF signaling must be functioning as effectively two different pathways”. However, an alternative explanation for the different expression patterns of Pea3 in epithelium and mesenchyme is a differential distribution of competence factors. To conclusively show that FGF signaling has different activity patterns in the two different tissues, the authors would need to directly analyze FGF signaling (e.g. using pERK stainings). Alternatively, they could soften their conclusion.
b) The argument that “[…] the peak-widths of Shh target gene expression were consistently somewhat larger than those of the other components and of its ligand (Fig.1b) making it likely that Shh was the fast-diffusing component compared to the other two” is not appropriate, since all components in reaction-diffusion systems have the same normalized range even if diffusion coefficients are different (i.e. there is only a single wave-length). Please adjust this argument accordingly.
c) Marcon et al. 2016 and Diego et al. 2018 have already made a number of observations that are in line with the conclusions presented here (stabilizing and destabilizing cycles, necessity of a

© 2020. Published by The Company of Biologists under the terms of the Creative Commons Attribution License (https://creativecommons.org/licenses/by/4.0/).

destabilizing module smaller than the size of the full system, minimal networks, pattern phases) that would be useful to discuss in the main text and Supplementary Notes.
d) Please mention as a caveat in the main text that only linear but not more realistic Hill-type interactions were considered.
e) The Supplementary Notes would benefit from extensive proofreading. It would also be good to number the supplementary figures and the figures in the Supplementary Notes in a unified manner.

3) Software and statistics a) The authors kindly provide the code for image and mathematical analysis. The utility of the scripts could be further increased by providing example data sets for the kymographs. Furthermore, it would be ideal to provide more information about the conditions needed to execute the R scripts (e.g. R version number appropriate for the dependent packages; mention that it only runs on Unix-based systems due the doMC package dependency).
b) In general, the manuscript is based on good statistics with four experimental repeats. However, the authors should also include a measure of the experimental variance and sample number in Fig. 1b. and Fig. S3. For Id1 in Fig. 1b, the traces in the lower panel do not seem to agree well with the in situ images shown in the panel above.
c) Please also add a measure of variance to the data in Fig. 6b if possible (e.g. by bootstrapping).

First revision

Author response to reviewers' comments

Economou ms Development reviews & responses
Responses to referees in blue

Reviewer 1 Advance Summary and Potential Significance to Field:
SUMMARYIn this study, Economou et al. aim to understand the mechanism driving periodic patterning of rugae in the mammalian palate. Their overall strategy is to compare experimental data with results from simulations, and instead of suggesting just a single model, they show how they can constrain the space of possible models that are consistent with their data. I very much admire this approach.

However, I do have some concerns with how the comparison between data and models is performed, which I outline below.

Reviewer 1 Comments for the Author:
MAJOR COMMENTS

1. PHASE DIFFERENCES AMONGST PATHWAY COMPONENTS

The authors measure transcriptional readouts of relevant signalling pathways to reveal their phase-relationship to the Hh+ rugae. They then use this to constrain their model, which focuses on signalling pathways in aggregate rather than specifically modelling ligands / pathway activation separately.
Looking for phase relations between pathways is a good strategy and has been exploited with success in other systems (e.g. the limb, Raspopovic et al. 2014). However, in these studies, both experimental and modelling results revealed that multiple elements of the signalling pathway can be expressed out of phase with each other e.g. BMP2 and activated BMP signalling (pSmad) are out of phase with each other as digits are patterned. Therefore it is not obvious a priori that modelling each signalling pathway by a single variable is sufficient. Can the authors comment on this?
We agree that there may be other inputs to each of our targets but nonetheless we feel that they represent practicable measurement points for the system. We have incorporated more explicit caveats and comments on the risks and limitations of this chosen simplification on p.3, paras 2 and 3 and on p.12, para 2

© 2020. Published by The Company of Biologists under the terms of the Creative Commons Attribution License (https://creativecommons.org/licenses/by/4.0/).

Two minor points: a) is there data from the literature describing ligand / receptor expression in the rugae – this would be useful to summarize for the reader.
We have amended the section on the mFGF versus eFGF pathways (p.5, para 2) to explain that expression data for FGF ligands and receptors identifies the most likely interactions in epithelium versus mesenchyme fairly cleanly since there is quite good in situ data for these. However, for canonical Wnts, the various Wnt receptors, and BMPs there is patchy data most of which does not distinguish rugal from non-rugal and/or epithelial from mesenchymal expression.

b) I do not necessarily agree with statements such as: "It may be argued that conceptually and pharmacologically, the most intelligible and accessible level of description is the signaling pathway". What is the justification?
As mentioned above, we have amended the text to more thoroughly justify, but also provide caveats for, our chosen simplification (p.3, paras 2 and 3 and on p.12, para 2).

2. INTERPRETATION OF PATHWAY INHIBITION - EXPERIMENTAL DATA
The authors perform interesting perturbation experiments implicating BMP/HH/WNT/FGF pathways in rugae formation and maintenance. However I have the following concerns with these experiments:

a) To understand pattern formation (which is the focus of this study), it seems necessary to focus on yet-to-be-formed Hh+ stripes, since any Hh+ stripes that have already formed may be maintained by a different mechanism. If I understand correctly, the E13.5 explants will already have r1-5 & r8, and so shouldn't the analysis only focus on r6/r7 whose formation occurs in the presence of drug? (This numbering is not my areas of expertise, but it would be good to clarify this.) Narrowing in on these newly-forming rugae, it seems to me that the interpretation of the data may be somewhat different e.g. is cyclopamine significantly disrupting pattern formation? (similar to results in Economou et al. 2012)
The fact that the inhibition has similar effects on both nascent and established stripes supports our assumption that the RD network is still operating to maintain the established Hh+ stripes and that as far as one can tell the network contains the same structure. Focusing on newly formed versus older rugae would, in practice, have made little difference to the analysis. (Our kinematic experiments show that the network for new rugae is indeed different despite all of the inhibiton and phase factors being the same.) Moreover, it is precisely the innovation of our approach that we are using perturbations to an established steady state rather than stripe formation ab initio to provide constraints to the allowable models.

b) The authors focus their interpretation on the levels of Hh following perturbation, and use this to constrain their mathematical models. However, it is unclear how the value of "mean Hh" is sufficient to capture the response of the system to the drug treatments. One could imagine that the spacing of the Hh+ stripes could change; there could be warping and misorientation of the Hh+ stripes; or there could be a complete failure to undergo pattern formation. Can the authors provide more quantification of their expression data to show that changes in Hh patterning following drug treatment can be entirely captured by "mean Hh", specifically for newly forming rugae as outlined above.
We have now measured the wavelengths for the inhibition experiments (supplementary figs s2 and s3, p.4 para 3 and p.13 paras 3 to p.14 para 2) and show quantitatively that wavelengths and stripe positions do not change with perturbation while stripe intensity and width do.

3. INTERPRETATION OF PATHWAY INHIBITION – MODELLING RESULTS

Related to the above point, the authors focus their attention on the "mean Hh" level following in silico perturbation. Can they provide evidence that their in silico perturbations are only affecting "mean Hh" level and not affecting other pattern parameters (e.g. wavelength, ability to break symmetry) - in other words, provide more evidence that the schematic in Figure 2b is representative of their modelling results. Here, it would be helpful not only to show when "mean Hh" does accurately capture their simulation results, but also provide clear examples / intuition of where this assumption fails.
E.g. I am somewhat surprised that their perturbations do not significantly affect pattern

© 2020. Published by The Company of Biologists under the terms of the Creative Commons Attribution License (https://creativecommons.org/licenses/by/4.0/).

wavelength, since similar types of model (e.g. limb, Sheth et al. 2012, Raspopovic et al. 2014) show that changing reaction parameters naturally alters pattern wavelengths.
We now include a detailed description of the types of response of the waves to inhibition (p.6 para 2 and 3, p.17 para 1) along with examples of perturbation simulation effects including both those where periodicity is lost and the vast majority where wavelength is unchanged (supplementary fig S8). We show how under these conditions the change in mean captures the simulation results and those changes in wave width and level seen in our experimental system (supplementary fig s8). We also report the proportion of parameter sets where the number of waves is maintained, and the proportion of parameter sets where waves appear to be behaving as seen in the experimental system (supplementary tables s1 and s2). The lack of wavelength effects is perhaps not too surprising given the smallness of the perturbations, the fact that we do not perturb diffusivity.

4. MODELLING TIMING OF GENE EXPRESSION DATA
In Figure 6, elegant experimental data suggests that some pathway elements develop periodic patterns later than others. This data is interesting and helps to inform what pathways are required for the initial periodic patterning.

However, a simple-minded interpretation of the data would be that BMP and mFGF are not required for the (initial) periodic patterning of rugae i.e. are not required for the DDI. Perhaps they are required for later refinement/maintenance of the pattern. Is this interpretation correct? If so, why do the authors consider BMP and mFGF in their models for DDI? (e.g. in Fig 3, BMP is included). Equally, can the authors provide evidence that their models in Figure 7 predict the observation “BMP and m-FGF responses trail Hh” and are not involved in DDI?
Yes, this interpretation is correct, but nevertheless (seemingly redundantly) BMP and mFGF fulfill all the criteria for being bona fide DDI morphogens. This goes back to the finding in our 2012 paper that established stripes are still under the control of a Turing system (they bifurcate when perturbed) as well as being perturbed by BMP and mFGF inhibition (this work). This is why we include them in the networks that we consider. Why they lag the other factors is something we are reluctant to speculate about but we have added a sentence in the final paragraph of the Discussion that opens this question for the reader's consideration.

A minor comment: the kymograph in Figure 6 provides a nice view of the dynamics of the patterning process. What are the horizontal “bands” in the figure? Do they complicate the interpretation (I’m particularly looking at Gli1).
The horizontal dark bands in the red channel are stages for which too few specimens were obtained to allow interpolation. We have added a sentence in the figure legend to explain this.

Reviewer 2 Advance Summary and Potential Significance to Field:
In this excellent paper, Economou et al. provide a new framework to clarify how multiple signaling pathways together organize the formation of periodic rugae during mouse oral palate development. The authors first analyzed how rugae patterning was affected when four major signaling pathways were inhibited, and then carefully quantified the spatiotemporal responses to these signaling pathways. Perturbation of signaling using small molecule inhibitors implied Shh and BMP as inhibitors and FGF and Wnt as activators of rugae patterning. Shh and Wnt target genes spatially overlapped, whereas the expression of FGF and BMP targets was interdigitated with respect to Shh/Wnt responses. Furthermore, the authors found temporal differences in target gene expression patterns, and the response to these signaling pathways also varied in neighboring tissues suggesting the presence of mesenchymal and epithelial-specific FGF signaling pathways. The authors then set out to clarify how the four signaling pathways must be wired together to explain the experimental findings. Using different degrees of abstraction based on self-organizing reaction-diffusion systems, they identified an early interaction between Wnt, Shh and epithelial FGF with a later incorporation of BMP and mesenchymal FGF as the most parsimonious model. The paper is very well written and combines a good mix of new experimental findings – including beautiful and highly informative time course data – and in depth-mathematical analyses to suggest a new network of signaling interactions for rugae patterning.

© 2020. Published by The Company of Biologists under the terms of the Creative Commons Attribution License (https://creativecommons.org/licenses/by/4.0/).

Reviewer 2 Comments for the Author:
MAJOR POINTS

1) Data analysis and controls a) Please quantify the data in Fig. 1a. Are only the levels changed, or is the width of the peaks also different with inhibitor treatments? Are potential changes in stripe width consistent with the models?
As per Reviewer 1, we have now added densitometry of the data in Fig.1A and quantified changes in stripe levels and width (supplementary figs s2 and s3), and shown graphically how inhibition can widen or narrow stripes (supplementary fig s8).

b) Please show the efficacy of the drugs by staining for the respective target genes (as has already been done for SU-5402 and cyclopamine in Economou et al. 2012).
We have now included our control data for inhibitor efficacy as requested (Fig. s1)

c) The manuscript and Supplementary Notes state “data not shown” in multiple instances. The authors should either show the data or remove the claims associated with these statements.
We have removed all instances of “data not shown” including additional supplementary figures to support the claims where necessary (supplementary figs s15 and s 16).

2) Perturbations and predictions
a) Please determine how each pathway (with the readout target genes shown in Fig. 1b) responds to inhibitor treatment. Is this consistent with the predictions of the models (e.g. Fig. S7)? In particular, according to Economou et al. 2012, cyclopamine treatment should broaden spry2 expression (i.e. Shh should normally inhibit FGF). This interaction is now very indirect (see Fig. 7c), and the authors should determine whether the corresponding expression changes in epithelial and mesenchymal FGF signaling targets are observed upon cyclopamine treatment.
Measuring all the outputs for all of the inhibitions is a significant technical and logistical challenge. This is not only because many of the probes give weak signal and often require many attempts to get specimens of sufficient quality but also, more immediately, we just do not currently have the ability to do all these experiments.

b) The authors provide a strong new prediction by stating that the core 3-node network is later modified by BMP and mFGF: Transient application of a BMP inhibitor should have different effects on the patterning outcome at different developmental stages. Testing this prediction, if technically possible, would provide very strong support for the conclusions of the paper.
We would love to do further experiments of several kinds on this system but we feel that this is beyond the scope of the current paper, especially considering that many controls for the transient-ness of an inhibitor would be required and may even be unfeasible (e.g. if wash-out is slow or multiphasic).

MINOR POINTS

1) Data analysis and presentation
a) Please describe how the normalization in Fig. 1b was performed.
We now describe how normalisation was performed (maximum signal set to 1) (p.14 para 2).
b) Please clarify why networks that are not strongly connected are shown in Fig. 3a. On p. 11, it is stated that they had been filtered out.
We now clarify in the figure legend that these are included in the figure for completeness.
c) Please show green/red data such as the one in Fig. 6a using a different color map that is suitable for a wider audience, and ideally show the channels separately.
We have changed the colour scheme to green/magenta. We also now include a kymograph of Shh alone, as well as the other targets alone and the composites.
d) I could not follow the argument “The two topologies cannot be combined into a three-component system […]”. For example, the network shown in the top middle of Fig. S5a is identical to the network iii shown in Fig. S8a. Please clarify.
We have rewritten this section and added a section and figure panel to explain in more detail what we mean by mediators and how topologies can be combined (p.5 bottom and supplementary fig s7a).

© 2020. Published by The Company of Biologists under the terms of the Creative Commons Attribution License (https://creativecommons.org/licenses/by/4.0/).

e) Please correct “um” to “uM” in Fig. S2
This has now been corrected (now Fig. S4) – thank you.
f) In Fig. S8b, the positive feedback loops indicated in magenta are not clear (e.g. the one outlined for network iv has an overall negative weight). Please clarify.
We have expanded the legend to this figure to clarify that in that instance the feedback loops in magenta refer to the net feedback between Wnt and BMP, which in all cases is a mutual inhibition and therefore a positive feedback.
g) Please provide the initial conditions and clarify how the system shown in Fig. 5a with purely inhibitory interactions can lead to stable spatiotemporal patterns. Intuitively, it seems that the concentration of all components should constantly decrease.
We have now included initial conditions for all our simulations in our methods (p.16 para 5) – we thank the reviewer for pointing out this oversight. We also mention in the Methods that all simulations have basal synthesis of components, which is why the depicted negative-only interactions do not deplete the system.
We have also included a paragraph (p.9 para 3) describing how the negative interactions in the specified topology produce the feedback loops required for a ddi. We also mark these feedbacks in fig 5a, as well as adding a schematic illustrating how combining them with the constraint table in fig 4 can be used to predict the response to perturbation.
Furthermore, “a_ii” in the Fig. 5 legend should be corrected to “a_ij”.
This has now been corrected – thank you.
h) In the Methods section, please mention how the position of the arrowheads in Fig. 6a was determined. If it was done manually, please add an unbiased alternative method.
We now state in the figure legend that the arrowheads indicate approximate positions and have expanded on our correlation analysis (p.11 top, p.14 top, fig 6 d,e, and supplementary fig s15) to provide an unbiased method for determining the timing of transcriptional events relative to the onset of Shh expression.

2) Manuscript organization
a) The authors state that “[...] FGF signaling must be functioning as effectively two different pathways”. However, an alternative explanation for the different expression patterns of Pea3 in epithelium and mesenchyme is a differential distribution of competence factors. To conclusively show that FGF signaling has different activity patterns in the two different tissues, the authors would need to directly analyze FGF signaling (e.g. using pERK stainings). Alternatively, they could soften their conclusion.
Since we include compentence factors such as receptors as part of the functioning of a pathway, we do not distinguish distribution of ligand from receptors, transducers, etc. We have clarified (p.5, para.1) that we define a pathway as its transcriptional read-out, which incorporates ligand, receptor and other competence factors.

b) The argument that “[...] the peak-widths of Shh target gene expression were consistently somewhat larger than those of the other components and of its ligand (Fig.1b) making it likely that Shh was the fast-diffusing component compared to the other two” is not appropriate, since all components in reaction-diffusion systems have the same normalized range even if diffusion coefficients are different (i.e. there is only a single wave-length). Please adjust this argument accordingly.
We thank the referee for pointing out the error in our reasoning. We have amended this section (p.8, para 2) to reflect that this was a simplifying initial assumption based in part on our previous results identifying Shh as a likely "inhibitor" in this system, which usually means it is the more diffusive morphogen.

c) Marcon et al. 2016 and Diego et al. 2018 have already made a number of observations that are in line with the conclusions presented here (stabilizing and destabilizing cycles, necessity of a destabilizing module smaller than the size of the full system, minimal networks, pattern phases) that would be useful to discuss in the main text and Supplementary Notes.
Were have now included additional mention, in both the main text and supplemental note, noting the many consistencies between our findings and those from the two papers.

d) Please mention as a caveat in the main text that only linear but not more realistic Hill-type interactions were considered.

© 2020. Published by The Company of Biologists under the terms of the Creative Commons Attribution License (https://creativecommons.org/licenses/by/4.0/).

We have mentioned in the main text (p.5, para 2) that linear equations were used, including the adjective "simple" to convey this point (which will be obvious to anyone who cares).

e) The Supplementary Notes would benefit from extensive proofreading. It would also be good to number the supplementary figures and the figures in the Supplementary Notes in a unified manner.
The Supplementary Notes have now undergone detailed proof reading. We have changed the numbering system so SN1, SN2 etc.

3) Software and statistics
a) The authors kindly provide the code for image and mathematical analysis. The utility of the scripts could be further increased by providing example data sets for the kymographs. Furthermore, it would be ideal to provide more information about the conditions needed to execute the R scripts (e.g. R version number appropriate for the dependent packages; mention that it only runs on Unix-based systems due the doMC package dependency).
We now provide the raw trace data from which the kymographs are constructed.
We have also annotated the code to include the version numbers used for the various packages as well as their dependencies.
We have also added to the Readme file that the simulations will only run on Unix-based systems due to the doMC package.

b) In general, the manuscript is based on good statistics with four experimental repeats. However, the authors should also include a measure of the experimental variance and sample number in Fig. 1b. and Fig. S3. For Id1 in Fig. 1b, the traces in the lower panel do not seem to agree well with the in situ images shown in the panel above.
We have included a measure of standard deviation on the traces in fig 1b and supplementary fig s6, along with sample numbers.
We also point out that while Id1 the pattern may only be seen weakly in the given image, the trace is averaged over a number of adjacent sections, and the same pattern is found repeatedly through our quantifications (see kymographs).

c) Please also add a measure of variance to the data in Fig. 6b if possible (e.g. by bootstrapping).
We have now added 95 % confidence intervals through bootstrapping.

<u>Second decision letter</u>

MS ID#: DEVELOP/2020/190553

MS TITLE: Perturbation analysis of a multi-morphogen Turing Reaction-Diffusion stripe patterning system reveals key regulatory interactions

AUTHORS: Andrew D. Economou, Nicholas A.M. Monk, and Jeremy B.A. Green

I have now received all the referees reports on the above manuscript, and have reached a decision. The referees' comments are appended below, or you can access them online: please go to BenchPress and click on the 'Manuscripts with Decisions' queue in the Author Area.

The overall evaluation is positive and we would like to publish a revised manuscript in Development, provided that the referees' comments can be satisfactorily addressed. Please attend to all of the reviewers' comments in your revised manuscript and detail them in your point-by-point response. If you do not agree with any of their criticisms or suggestions explain clearly why this is so.

We are aware that you may currently be unable to access the lab to undertake experimental revisions. If it would be helpful, we encourage you to contact us to discuss your revision in greater

© 2020. Published by The Company of Biologists under the terms of the Creative Commons Attribution License (https://creativecommons.org/licenses/by/4.0/).

detail. Please send us a point-by-point response indicating where you are able to address concerns raised (either experimentally or by changes to the text) and where you will not be able to do so within the normal timeframe of a revision. We will then provide further guidance. Please also note that we are happy to extend revision timeframes as necessary.

Reviewer 1

Advance summary and potential significance to field

SUMMARY

In this study, Economou et al. aim to understand the mechanism driving periodic patterning of rugae in the mammalian palate. Their overall strategy is to compare experimental data with results from simulations, and instead of suggesting just a single model, they show how they can constrain the space of possible models that are consistent with their data. I very much admire this approach.

During the initial submission, I had some concerns with how the comparison between data and models is performed. The authors' revisions have allayed these concerns and much improved the manuscript.

Comments for the author

MINOR COMMENT

I just have a minor comment aimed at clarifying one point:

In response to my comment #2 "INTERPRETATION OF PATHWAY INHIBITION - EXPERIMENTAL DATA", the authors provide new analysis of their data quantifying the effect of inhibitors on rugae patterns. I think Fig S3 is an excellent addition and nicely quantifies the pattern.

However, this new quantification does not change the main thrust behind my initial comment but rather highlights it - specifically that there appears to be a qualitatively different effect of HH & FGF inhibition on anterior/posterior i.e. already formed / newly forming stripes (apologies if i have anterior-posterior mixed up!) - and also that this difference is not expected/predicted/accounted for in their modelling.

(BTW, I think this could be an interesting observation by itself - and may well fit in with some of the temporal data the authors describe later.)

Can the authors add a sentence or two better describing why these more major perturbations to stripe patterns (stripe fusion/disappearance i.e. red points in Fig S3b) do not compromise their modelling approach. It would be fine to say that they focus their attention just on the weakly perturbed stripes (white points in Fig S3b), but speculate that exactly the same mechanism is involved in newly forming stripes but for some reason the effect of the perturbation is stronger in these newly forming stripes than on already formed stripes. [And also helpful to refer the reader to the 2012 paper showing the already formed stripes can bifurcate following perturbation suggesting maintenance involves turing mechanism]

Reviewer 2

Advance summary and potential significance to field

In their revised manuscript, the authors have appropriately addressed all reviewersÂ’ comments.

Comments for the author

A few very minor comments before publication (no re-review necessary):

© 2020. Published by The Company of Biologists under the terms of the Creative Commons Attribution License (https://creativecommons.org/licenses/by/4.0/).

- Abstract: The new abstract is more difficult to understand than the previous one. For example, the new wording “[…] identified the much-constrained number […]” is not entirely clear, and I suggest rewording that sentence. Also, a comma between “inhibitor” and “with” would be helpful for the readers.
- Fig. 5B: The labeling of the graphs differs from the previous version of the manuscript and seems to be wrong since the effect of inhibiting all FGF signaling should be “[…] neither net increase nor net decrease […]” but “[…] a flatter waveform”.
- Code: I could not find the link to the GitLab deposition mentioned in the main text.

Second revision

Author response to reviewers' comments

Response to Reviewers

Reviewer 1
I just have a minor comment aimed at clarifying one point:

In response to my comment #2 "INTERPRETATION OF PATHWAY INHIBITION - EXPERIMENTAL DATA", the authors provide new analysis of their data quantifying the effect of inhibitors on rugae patterns. I think Fig S3 is an excellent addition and nicely quantifies the pattern.However, this new quantification does not change the main thrust behind my initial comment but rather highlights it - specifically that there appears to be a qualitatively different effect of HH & FGF inhibition on anterior/posterior i.e. already formed / newly forming stripes (apologies if i have anterior-posterior mixed up!) - and also that this difference is not expected/predicted/accounted for in their modelling.
Can the authors add a sentence or two better describing why these more major perturbations to stripe patterns (stripe fusion/disappearance i.e. red points in Fig S3b) do not compromise their modelling approach. It would be fine to say that they focus their attention just on the weakly perturbed stripes (white points in Fig S3b), but speculate that exactly the same mechanism is involved in newly forming stripes but for some reason the effect of the perturbation is stronger in these newly forming stripes than on already formed stripes. [And also helpful to refer the reader to the 2012 paper showing the already formed stripes can bifurcate following perturbation suggesting maintenance involves turing mechanism]
--- We have added two new (long!) sentences (page 5, middle paragraph) conveying exactly the point that the reviewer makes, which we agree is an important consideration.

Reviewer 2 Comments for the author
A few very minor comments before publication (no re-review necessary):
Abstract: The new abstract is more difficult to understand than the previous one. For example, the new wording “[…] identified the much-constrained number […]” is not entirely clear, and I suggest rewording that sentence. Also, a comma between “inhibitor” and “with” would be helpful for the readers.
--- We have re-written the Abstract, including the sentence mentioned, and added the comma for clarity as suggested. We would be happy for the handling editor to suggest different or additional changes to make the Abstract better and clearer.

Fig. 5B: The labeling of the graphs differs from the previous version of the manuscript and seems to be wrong since the effect of inhibiting all FGF signaling should be “[…] neither net increase nor net decrease […]” but “[…] a flatter waveform”.
--- We have corrected Fig.5 in which the labelling had indeed become rearranged.

Code: I could not find the link to the GitLab deposition mentioned in the main text.
--- We have now included the GitLab link in the Materials & Methods (p.15)

© 2020. Published by The Company of Biologists under the terms of the Creative Commons Attribution License (https://creativecommons.org/licenses/by/4.0/).

--- We have uploaded a PDF version of the manuscript with tracked changes for your convenience.

Third decision letter

MS ID#: DEVELOP/2020/190553

MS TITLE: Perturbation analysis of a multi-morphogen Turing Reaction-Diffusion stripe patterning system reveals key regulatory interactions

AUTHORS: Andrew D. Economou, Nicholas A.M. Monk, and Jeremy B.A. Green
ARTICLE TYPE: Research Article

I am happy to tell you that your manuscript has been accepted for publication in Development, pending our standard ethics checks.

© 2020. Published by The Company of Biologists under the terms of the Creative Commons Attribution License (https://creativecommons.org/licenses/by/4.0/).
